# Effectiveness and Safety of a Novel Care Model for the Management of Type 2 Diabetes at 1 Year: An Open-Label, Non-Randomized, Controlled Study

**DOI:** 10.1007/s13300-018-0373-9

**Published:** 2018-02-07

**Authors:** Sarah J. Hallberg, Amy L. McKenzie, Paul T. Williams, Nasir H. Bhanpuri, Anne L. Peters, Wayne W. Campbell, Tamara L. Hazbun, Brittanie M. Volk, James P. McCarter, Stephen D. Phinney, Jeff S. Volek

**Affiliations:** 1 Medically Supervised Weight Loss, Indiana University Health Arnett, Lafayette, IN USA; 2 Virta Health, San Francisco, CA USA; 3 Independent Consultant, Lafayette, CA USA; 4 Keck School of Medicine, University of Southern California, Los Angeles, CA USA; 5 Department of Nutrition Science, Purdue University, West Lafayette, IN USA; 6 Department of Genetics, Washington University School of Medicine, St. Louis, MO USA; 7 Department of Human Sciences, The Ohio State University, Columbus, OH USA

**Keywords:** Beta-hydroxybutyrate, Carbohydrate restriction, HbA1c, Ketosis, Type 2 diabetes, Weight loss

## Abstract

**Introduction:**

Carbohydrate restriction markedly improves glycemic control in patients with type 2 diabetes (T2D) but necessitates prompt medication changes. Therefore, we assessed the effectiveness and safety of a novel care model providing continuous remote care with medication management based on biometric feedback combined with the metabolic approach of nutritional ketosis for T2D management.

**Methods:**

We conducted an open-label, non-randomized, controlled, before-and-after 1-year study of this continuous care intervention (CCI) and usual care (UC). Primary outcomes were glycosylated hemoglobin (HbA_1c_), weight, and medication use. Secondary outcomes included fasting serum glucose and insulin, HOMA-IR, blood lipids and lipoproteins, liver and kidney function markers, and high-sensitivity C-reactive protein (hsCRP).

**Results:**

349 adults with T2D enrolled: CCI: *n* = 262 [mean (SD); 54 (8) years, 116.5 (25.9) kg, 40.4 (8.8) kg m^2^, 92% obese, 88% prescribed T2D medication]; UC: *n* = 87 (52 (10) years, 105.6 (22.15) kg, 36.72 (7.26) kg m^2^, 82% obese, 87% prescribed T2D medication]. 218 participants (83%) remained enrolled in the CCI at 1 year. Intention-to-treat analysis of the CCI (mean ± SE) revealed HbA_1c_ declined from 59.6 ± 1.0 to 45.2 ± 0.8 mmol mol^−1^ (7.6 ± 0.09% to 6.3 ± 0.07%, *P* < 1.0 × 10^−16^), weight declined 13.8 ± 0.71 kg (*P* < 1.0 × 10^−16^), and T2D medication prescription other than metformin declined from 56.9 ± 3.1% to 29.7 ± 3.0% (*P* < 1.0 × 10^−16^). Insulin therapy was reduced or eliminated in 94% of users; sulfonylureas were entirely eliminated in the CCI. No adverse events were attributed to the CCI. Additional CCI 1-year effects were HOMA-IR − 55% (*P* = 3.2 × 10^−5^), hsCRP − 39% (*P* < 1.0 × 10^−16^), triglycerides − 24% (*P* < 1.0 × 10^−16^), HDL-cholesterol + 18% (*P* < 1.0 × 10^−16^), and LDL-cholesterol + 10% (*P* = 5.1 × 10^−5^); serum creatinine and liver enzymes (ALT, AST, and ALP) declined (*P* ≤ 0.0001), and apolipoprotein B was unchanged (*P* = 0.37). UC participants had no significant changes in biomarkers or T2D medication prescription at 1 year.

**Conclusions:**

These results demonstrate that a novel metabolic and continuous remote care model can support adults with T2D to safely improve HbA_1c_, weight, and other biomarkers while reducing diabetes medication use.

**ClinicalTrials.gov Identifier:**

NCT02519309.

**Funding:**

Virta Health Corp.

**Electronic supplementary material:**

The online version of this article (10.1007/s13300-018-0373-9) contains supplementary material, which is available to authorized users.

## Plain Language Summary

Treatments for type 2 diabetes (T2D) have improved, yet T2D and being overweight are still significant public health concerns. Blood sugar in patients with T2D can improve quickly when patients eat significantly fewer dietary carbohydrates. However, this demands careful medicine management by doctors, and patients need support and frequent contact with health providers to sustain this way of living. The purpose of this study was to evaluate if a new care model with very low dietary carbohydrate intake and continuous supervision by a health coach and doctor could safely lower HbA1c, weight and need for medicines after 1 year in adults with T2D. 262 adults with T2D volunteered to participate in this continuous care intervention (CCI) along with 87 adults with T2D receiving usual care (UC) from their doctors and diabetes education program. After 1 year, patients in the CCI, on average, lowered HbA1c from 7.6 to 6.3%, lost 12% of their body weight, and reduced diabetes medicine use. 94% of patients who were prescribed insulin reduced or stopped their insulin use, and sulfonylureas were eliminated in all patients. Participants in the UC group had no changes to HbA1c, weight or diabetes medicine use over the year. These changes in CCI participants happened safely while dyslipidemia and markers of inflammation and liver function improved. This suggests the novel care model studied here using dietary carbohydrate restriction and continuous remote care can safely support adults with T2D to lower HbA1c, weight, and medicine use.

**Electronic supplementary material:**

The online version of this article (10.1007/s13300-018-0373-9) contains supplementary material, which is available to authorized users.

## Introduction

The number of people living with diabetes worldwide nearly quadrupled since 1980, estimated at 422 million in 2014 [1]. In the USA, the Centers for Disease Control reports 30.3 million adults presently live with diabetes, and it is among the leading causes of death [2]. Treatment modalities for type 2 diabetes (T2D) have demonstrated varying success. Intensive lifestyle interventions are effective treatments for obese individuals with T2D when weight loss is achieved and sustained [3]. Evidence for improved cardiovascular outcomes in patients with T2D prescribed glucagon-like peptide 1 receptor agonists (GLP-1) and sodium glucose co-transporter 2 inhibitors (SGLT-2) is increasing [4, 5]. Forty percent of patients undergoing bariatric surgery demonstrate substantial improvements in glycemic control after 1 year and many achieve T2D remission [6]. Despite advancements in treatment options, cost, side effects, adherence, and disease progression remain barriers.

Guidelines for T2D management recommend lifestyle change and weight loss [7, 8]. However, a fraction of individuals are successful at long-term weight loss maintenance and true disease remission is uncommon [3, 9]. Mediterranean-style, DASH, and plant-based diets, sometimes with prescribed energy restriction, are recommended, but effectiveness data are limited [7] and low fat diets have not been shown to be superior for weight loss [10]. Commercially available weight loss programs have demonstrated short-term success in glycemic control, but continued success at 1 year is uncommon [11].

Glycemic control can be achieved quickly with carbohydrate restriction via very low energy diets (400–800 kcal day^−1^; VLCD) [12]. However, VLCD are necessarily temporary and outcomes often revert when patients resume former dietary patterns. Alternatively, nutritional ketosis, achieved by consuming moderate protein and greatly reduced carbohydrate, results in similarly increased serum beta-hydroxybutyrate (BHB) concentrations as observed during VLCD, which signifies a shift to using fat as the body’s primary fuel source [12]. This nutritional therapy may help patients achieve sustainable glycemic control without prescribed energy restriction. Benefit may accrue from decreased circulating glucose and insulin [13], ketone signaling [14, 15], or eventual weight loss. Studies utilizing carbohydrate restriction observed improved glycemic control and cardiometabolic markers, but were often short-term trials of small groups, excluded subjects prescribed insulin, or infrequently monitored or achieved ketosis [16–20].

The chronic nature of diabetes care presents an additional challenge requiring sustained behavioral change that is difficult to support with traditional medical care including infrequent provider contact [21]. Adherence to lifestyle changes may be poor in the absence of support from providers and peers. We therefore hypothesized that a comprehensive care model that supports patients to achieve sustained nutritional ketosis while eating to satiety may have robust benefits in T2D management. This intervention utilizes continuous care through intensive, digitally enabled support including telemedicine access to a medical provider (physician or nurse practitioner), health coaching, nutrition and behavior change education and individualized care plans, biometric feedback, and peer support via an online community. Thus, the purpose of this study was to assess the effectiveness and safety of a novel care model (Virta Clinic, Virta Health; San Francisco, CA, USA) for the management of T2D after 1 year. Secondary aims were (1) to determine if a difference in primary outcomes existed between participants who self-selected on-site versus web-based education delivery and (2) explore the time course of biomarker change at 70 days and 1 year into the CCI. Primary endpoints to assess effectiveness of the intervention were change in glycosylated hemoglobin (HbA_1c_), body weight, and medication prescription after 1 year. Secondary outcomes, including clinical biomarkers of associated physiological systems and adverse events, were assessed to determine safety of the intervention.

## Methods

We utilized an open-label, non-randomized, controlled, before-and-after study design with a cohort of patients who self-selected to participate in the metabolic and continuous care intervention (CCI) for T2D and a comparison group of patients who self-selected to participate while receiving their usual care (UC) from their own medical providers and diabetes education program (Clinicaltrials.gov Identifier NCT02519309). Adults diagnosed with T2D were recruited via clinical referrals, local advertisements, and word of mouth in Lafayette, Indiana, USA and surrounding region from August 2015 through March 2016. This study was approved by the Franciscan Health Lafayette Institutional Review Board. All procedures performed in studies involving human participants were in accordance with the ethical standards of the institutional and/or national research committee and with the 1964 Helsinki declaration and its later amendments or comparable ethical standards. Informed consent was obtained from all individual participants included in the study.

### Continuous Care Intervention

Participants in the CCI underwent history and physical exam followed by laboratory testing to ensure they met inclusion and exclusion criteria (Supplementary Materials A). Upon qualifying, CCI participants received biomarker tracking tools including a cellular-connected body weight scale (BT003, Body Trace; New York, NY, USA), a finger-stick blood glucose and ketone meter (Precision Xtra, Abbott; Alameda, CA, USA), and a blood pressure cuff if hypertension was diagnosed (BP742 N, Omron Healthcare, Inc.; Lake Forest, IL, USA). Access to a web-based software application (app) was provided for biomarker reporting and monitoring, education, and communication with remote care team (via telemedicine) consisting of a health coach and medical provider (physician or nurse practitioner) for advice and medication management. Social support was provided via an online peer community. Participants in the CCI retained their primary care provider (PCP) for conditions other than metabolic disease, and care coordination between the PCP and CCI provider occurred as needed. Frequency and type of biomarker tracking were individualized on the basis of care needs and recorded by participants in the app; initial participant instructions were to weigh and measure blood BHB concentration daily, and to measure blood glucose one to three times daily. The remote care team monitored this information; a medical provider made medication changes as indicated by the participant-reported biomarkers (Supplementary Materials B).

Participants were provided individualized nutrition recommendations that allowed them to achieve and sustain nutritional ketosis with a goal of 0.5–3.0 mmol L^−1^ blood BHB. Participants were encouraged to report daily hunger, cravings, energy, and mood on a four-point Likert scale. These ratings and BHB concentrations were utilized to adjust nutritional guidance. With the insulin resistance characteristic of T2D, patients typically require total dietary carbohydrates to be restricted to less than 30 g day^−1^ to achieve nutritional ketosis. Health coaches monitored blood BHB concentrations logged by participants and worked with participants individually to adjust dietary carbohydrate intake to a level that would allow them to achieve nutritional ketosis. Daily protein intake was initially targeted to a level of 1.5 g kg^−1^ of reference (i.e., medium-frame “ideal”) body weight and adjusted as necessary to aid participants in achieving nutritional ketosis based on participant-logged blood BHB concentrations. Participants were coached to incorporate dietary fats to satiety. Participants were advised to consume adequate intake of omega-3 (eicosapentaenoic acid and docosahexaenoic acid) and omega-6 (linoleic acid) polyunsaturated fats [22], while it was recommended that the remainder of their intake from fat come from both monounsaturated and saturated sources. Other aspects of the diet were individually prescribed to ensure safety, effectiveness, and satisfaction, including consumption of 3–5 servings of non-starchy vegetables and adequate mineral and fluid intake for the ketogenic state. At onset of dietary changes, participants were advised to consume a multivitamin, 1000–2000 IU vitamin D3, and up to 1000 mg omega-3 daily. If participants exhibited signs of magnesium depletion (e.g., muscle twitches or cramps), daily supplementation (500 mg magnesium oxide or 200 mg magnesium chloride) was suggested. If participants exhibited headaches, constipation, or lightheadedness, adequate sodium and fluid intake was recommended. BHB concentrations were also utilized as a marker of adherence to nutritional ketosis. Behavior change strategies were utilized by the remote care team and tailored to the needs of each participant to help achieve glycemic control. Examples of techniques utilized include education of natural consequences, shaping knowledge, goal setting, self-monitoring, feedback, monitoring and reinforcement from health coach and medical provider, self-belief, social support, relapse prevention, associations, and repetition.

Participants in the CCI self-selected how they would receive most of their education: (1) via on-site group education classes that met weekly for 12 weeks, bi-weekly for 12 weeks, and monthly for 6 months (*n* = 136; CCI-onsite) or (2) via web-based, recorded educational content viewed independently through the app (*n* = 126; CCI-web). Educational content was the same regardless of delivery method (Supplementary Materials C), and all other aspects of care were the same. During on-site classes, health coaches presented educational content and medical providers met with participants individually. Participants receiving web-based education could schedule visits with the CCI medical provider if desired. Apart from education delivery, both groups received remote care from health coaches.

### Usual Care

Participants in the UC group were patients with diagnosed T2D who were recently referred to the local diabetes education program by their primary care physician or endocrinologist where they were counseled by registered dietitians on diabetes self-management, nutrition, and lifestyle [7]. Medical care for their T2D was provided by their primary care physician or endocrinologist. No modification to the care that they received for their T2D was made by the study. This group was observed at baseline and 1 year as reference for typical disease treatment and progression over 1 year within the same geographical, health care, and laboratory locations. UC participants attended a separate information session and informed consent was obtained followed by laboratory testing to ensure they met all inclusion and exclusion criteria. Patients were informed that the trial also had an intervention arm and could participate in that group if they chose to do so.

### Outcome Measures

In-clinic vital signs and anthropometrics were obtained at baseline, 70-days (CCI only [23]), and 1-year follow-up. Height was assessed via stadiometer for calculation of body mass index. In-clinic weight for all participants was measured to the nearest 0.1 lb (Model 750, Detecto; Webb City, MO, USA) and converted to kg. In-clinic blood pressure was obtained manually by trained staff after participants rested in a seated position for 5 min. Adverse events were reported to the Principal Investigator and reviewed by the Institutional Review Board.

Fasted blood draws occurred at baseline, 70-days (CCI only [23]), and 1-year follow up. Blood analytes were determined via standard procedures at a Clinical Laboratory Improvement Amendment (CLIA) accredited laboratory on the day of sample collection or from stored serum (Supplementary Materials D).

### Statistical Analysis

Statistical analyses were performed using JMP software (version 5.1, SAS Institute; Cary, SC, USA) for all analyses except multiple imputation, for which we used Stata software (version 11, StataCorp; College Station, TX, USA). Multiple imputation was used to estimate means and standard errors that include the variability between imputations. Missing values were estimated from 700 imputations from multivariate normal regression. The number of missing data points for each measure can be determined from the difference between all participants and completers in Tables 1 and S1. Across all biomarkers, 4% of baseline values and 24% of 1-year values were missing (due to dropout, incalculable values, or inability to procure timely samples) and thus imputed to conduct the intention-to-treat analysis. Two-sample *t* tests were used to test whether baseline differences and differences between 1-year biomarker changes were significant. Within-group changes were tested using paired *t* test and analysis of covariance (ANCOVA) when adjusted for baseline covariates (sex, age, baseline BMI, insulin use versus non-use, and African-American race). Although tables present triglyceride and hsCRP summary statistics in clinical units, significance levels were obtained from log-transformed values to reduce skewness. For completer analysis, percent change was calculated as the mean difference (Table 2) divided by the mean baseline value (Table 1). Significant changes in medication use and the proportion of patients with HbA_1c_at least 48 mmol mol^−1^ (≥ 6.5%) were tested using McNemar test with continuity correction in completers, and linear regression of the changes in the dichotomous states when missing outcome data were imputed. Standard deviations are presented within parentheses and standard errors following “±”. Nominal significance levels (*P*) are presented in tables; however, a significance level of *P* < 0.0017 ensures simultaneous significance at *P* < 0.05 with Bonferroni adjustment for the 30 variables examined. Results presented are intention-to-treat analyses (all), where missing values were estimated by imputation, unless otherwise noted. Participants who withdrew or lacked biomarkers at 1 year were not included in the analyses of completers.

**Table 1:** Baseline characteristics of the recruited sample, completers, and participants with missing data by treatment arm

	All		Completers with data		Dropout or missing data		Completers-Dropouts
	*N*	Mean (SD) or ±SE	*N*	Mean (SD) or ±SE	*N*	Mean (SD) or ±SE	Mean ± SE
Age (years)							
CCI-all education^a^	262	53.75 (8.35)	218	54.09 (8.35)	44	52.09 (8.25)	2.0 ± 1.37
Usual care^a^	87	52.33 (9.52)	78	51.71 (9.62)	9	57.78 (6.85)	− 6.07 ± 2.53*
CCI-all vs. usual care^b^		1.42 ± 1.14		2.38 ± 1.23*		− 5.69 ± 2.6*	
Female (%)							
CCI-all education^a^	262	66.79 ± 2.91	218	65.14 ± 3.23	44	75.0 ± 6.53	− 9.86 ± 7.28
Usual care^a^	87	58.62 ± 5.28	78	60.26 ± 5.54	9	44.44 ± 16.56	15.81 ± 17.47
CCI-all vs. usual care^b^		8.17 ± 6.03		4.88 ± 6.41		30.56 ± 17.8	
African American (%)							
CCI-all education^a^	262	6.87 ± 1.56	218	5.96 ± 1.6	44	11.36 ± 4.78	− 5.4 ± 5.05
Usual care^a^	87	0.0 ± 0.0	78	0.0 ± 0.0	9	0.0 ± 0.0	0.0 ± 0.0
CCI-all vs. usual care^b^		6.87 ± 1.56§		5.96 ± 1.6‡		11.36 ± 4.78*	
Years with type 2 diabetes							
CCI-all education^a^	261	8.44 (7.22)	217	8.4 (7.28)	44	8.61 (6.97)	− 0.21 ± 1.16
Usual care^a^	71	7.85 (7.32)	71	7.85 (7.32)		Not collected	
CCI-all vs. usual care^b^		0.59 (0.9)		0.56 ± 1.0			
Beta-hydroxybutyrate (mmol L^−1^)							
CCI-all education^a^	248	0.17 (0.15)	186	0.17 (0.15)	62	0.19 (0.16)	− 0.02 ± 0.02
Usual care^a^	79	0.15 (0.13)	59	0.14 (0.12)	20	0.17 (0.15)	− 0.03 ± 0.03
CCI-all vs. usual care^b^		0.02 ± 0.02		0.02 ± 0.02		0.02 ± 0.04	
Hemoglobin A_1c_ (mmol mol^−1^)							
CCI-all education^a^	262	59.55 (16.4)	204	58.35 (15.3)	58	63.49 (19.57)	− 28.66 ± 2.73
Usual care^a^	87	59.99 (19.24)	72	61.08 (19.89)	15	54.52 (14.87)	− 16.97 ± 4.48
CCI-all vs. usual care^b^		− 0.44 ± 2.3		− 2.73 ± 2.62		8.96 ± 4.59*	
Hemoglobin A_1c_ (%)							
CCI-all education^a^	262	7.60 (1.50)	204	7.49 (1.4)	58	7.96 (1.79)	− 0.47 ± 0.25
Usual care^a^	87	7.64 (1.76)	72	7.74 (1.82)	15	7.14 (1.36)	0.60 ± 0.41
CCI-all vs. usual care^b^		− 0.04 ± 0.21		− 0.25 ± 0.24		0.82 ± 0.42*	
Fasting glucose (mmol L^−1^)							
CCI-all education^a^	258	8.92 (3.41)	202	8.8 (3.28)	56	9.36 (3.83)	− 0.55 ± 0.56
Usual care^a^	86	8.67 (4.03)	71	8.71 (3.96)	15	8.5 (4.5)	0.21 ± 1.25
CCI-all vs. usual care^b^		0.25 ± 0.48		0.1 ± 0.52		0.86 ± 1.27	
Insulin all (pmol L^−1^)							
CCI-all education^a^	248	198.35 (165.85)	186	197.65 (167.17)	62	200.5 (163.21)	− 2.85 ± 24.1
Usual care^a^	79	202.17 (172.58)	59	206.68 (187.93)	20	188.77 (119.18)	17.99 ± 36.18
CCI-all vs. usual care^b^		− 3.82 ± 22.09		− 9.1 ± 27.36		11.74 ± 33.75	
C-peptide (nmol L^−1^)							
CCI-all education^a^	247	1.45 (0.71)	185	1.47 (0.72)	62	1.39 (0.69)	0.07 ± 0.1
Usual care^a^	79	1.38 (0.82)	59	1.35 (0.82)	20	1.49 (0.84)	− 0.14 ± 0.22
CCI-all vs. usual care^b^		0.07 ± 0.1		0.12 ± 0.12		− 0.09 ± 0.21	
HOMA-IR (insulin derived), all							
CCI-all education^a^	244	11.8 (13.14)	179	11.19 (12.75)	65	13.48 (14.12)	− 2.3 ± 1.99
Usual care^a^	78	10.64 (9.12)	56	11.31 (10.05)	22	8.94 (6.03)	2.36 ± 1.86
CCI-all vs. usual care^b^		1.16 ± 1.33		− 0.12 ± 1.65		4.54 ± 2.17	
HOMA-IR (insulin derived), excluding exogenous users							
CCI-all education^a^	172	11.77 (13.87)	129	11.00 (13.53)	43	14.09 (14.76)	− 3.08 ± 2.55
Usual care^a^	43	9.40 (8.25)	25	9.36 (9.39)	18	9.45 (6.61)	− 0.09 ± 2.44
CCI-all vs. usual care^b^		2.37 ± 1.64		1.64 ± 2.22		4.63 ± 2.74	
HOMA-IR (C-peptide derived)							
CCI-all education^a^	239	11.52 (7.15)	170	11.44 (6.26)	69	11.72 (9.04)	− 0.28 ± 1.19
Usual care^a^	72	11.16 (7.26)	47	10.56 (7.70)	25	12.29 (6.33)	− 1.73 ± 1.69
CCI-all vs. usual care^b^		0.36 ± 0.97		0.88 ± 1.22		− 0.56 ± 1.67	
Weight-clinic (kg)							
CCI-all education^a^	257	116.51 (25.94)	184	115.42 (24.62)	73	119.25 (29.01)	− 3.83 ± 3.85
Usual care^a^	83	105.63 (22.15)	69	106.79 (22.18)	14	99.94 (21.86)	6.84 ± 6.42
CCI-all vs. usual care^b^		10.87 ± 2.92§		8.63 ± 3.23†		19.3 ± 6.76†	
BMI (kg m^−2^)							
CCI-all education^a^	257	40.43 (8.81)	184	39.87 (7.88)	73	41.82 (10.75)	− 1.94 ± 1.39
Usual care^a^	83	36.72 (7.26)	69	37.14 (7.62)	14	34.66 (4.8)	2.48 ± 1.58
CCI-all vs. usual care^b^		3.7 ± 0.97‡		2.73 ± 1.09†		7.15 ± 1.8§	
Systolic blood pressure (mmHg)							
CCI-all education^a^	260	131.94 (14.09)	187	132.51 (14.54)	73	130.47 (12.84)	2.05 ± 1.84
Usual care^a^	79	129.8 (13.61)	67	128.72 (12.65)	12	135.83 (17.49)	− 7.12 ± 5.28
CCI-all vs. usual care^b^		2.14 ± 1.76		3.8 ± 1.88*		− 5.37 ± 5.27	
Diastolic blood pressure (mmHg)							
CCI-all education^a^	260	82.09 (8.25)	187	81.59 (8.05)	73	83.37 (8.67)	− 1.78 ± 1.17
Usual care^a^	79	82.0 (8.93)	67	81.1 (8.07)	12	87.0 (11.95)	− 5.9 ± 3.59
CCI-all vs. usual care^b^		0.09 ± 1.13		0.49 ± 1.15		− 3.63 ± 3.6	
Total cholesterol (mmol L^−1^)							
CCI-all education^a^	247	4.76 (1.07)	186	4.68 (1.03)	61	4.99 (1.15)	− 0.31 ± 0.17
Usual care^a^	79	4.76 (1.19)	59	4.72 (1.26)	20	4.88 (0.93)	− 0.16 ± 0.27
CCI-all vs. usual care^b^		− 0.0 ± 0.15		− 0.04 ± 0.18		0.11 ± 0.26	
LDL-cholesterol (mmol L^−1^)							
CCI-all education^a^	232	102.51 (32.89)	172	100.08 (32.56)	60	109.47 (33.13)	− 9.39 ± 4.94
Usual care^a^	70	101.50 (36.16)	48	100.38 (37.93)	22	103.95 (32.67)	− 3.58 ± 8.86
CCI-all vs. usual care^b^		1.01 ± 4.83		− 0.29 ± 6.01		5.51 ± 8.17	
Apo B (g L^−1^)							
CCI-all education^a^	248	1.05 (0.29)	186	1.03 (0.28)	62	1.1 (0.31)	− 0.06 ± 0.04
Usual care^a^	79	1.07 (0.28)	59	1.06 (0.3)	20	1.11 (0.24)	− 0.05 ± 0.07
CCI-all vs. usual care^b^		− 0.02 ± 0.04		− 0.02 ± 0.04		− 0.01 ± 0.07	
HDL-C (mmol L^−1^)							
CCI-all education^a^	247	1.09 (0.35)	186	1.1 (0.36)	61	1.08 (0.32)	0.02 ± 0.05
Usual care^a^	79	0.97 (0.29)	59	0.96 (0.29)	20	1.02 (0.29)	− 0.06 ± 0.08
CCI-all vs. usual care^b^		0.12 ± 0.04†		0.14 ± 0.05†		0.06 ± 0.08	
Triglycerides (mmol L^−1^)							
CCI-all education^a^	247	2.23 (1.62)	186	2.27 (1.73)	61	2.11 (1.25)	0.15 ± 0.2
Usual care^a^	79	3.2 (4.53)	59	3.36 (5.17)	20	2.72 (1.56)	0.64 ± 0.76
CCI-all vs. usual care^b^		− 0.97 ± 0.52*		− 1.09 ± 0.68		− 0.61 ± 0.38*	
Total/HDL-cholesterol							
CCI-all education^a^	247	4.72 (1.7)	186	4.65 (1.72)	61	4.93 (1.65)	− 0.28 ± 0.25
Usual care^a^	79	5.37 (2.42)	59	5.44 (2.63)	20	5.17 (1.72)	0.27 ± 0.52
CCI-all vs. usual care^b^		− 0.65 ± 0.29*		− 0.79 ± 0.36*		− 0.24 ± 0.44	
hsC-reactive protein (nmol L^−1^)							
CCI-all education^a^	249	81.33 (138.0)	193	85.62 (153.05)	56	66.76 (62.1)	18.86 ± 13.81
Usual care^a^	85	84.67 (82.1)	70	86.95 (86.95)	15	73.81 (73.81)	13.14 ± 19.14
CCI-all vs. usual care^b^		− 3.24 ± 12.48		− 1.33 ± 15.05		− 7.05 ± 18.19	
ALT (µkat L^−1^)							
CCI-all education^a^	257	0.51 (0.38)	201	0.52 (0.41)	56	0.47 (0.27)	0.05 ± 0.05
Usual care^a^	86	0.46 (0.33)	71	0.45 (0.34)	15	0.51 (0.29)	− 0.05 ± 0.09
CCI-all vs. usual care^b^		0.05 ± 0.04		0.07 ± 0.05		− 0.04 ± 0.08	
AST (µkat L^−1^)							
CCI-all education^a^	257	0.4 (0.25)	201	0.41 (0.28)	56	0.36 (0.15)	0.04 ± 0.03
Usual care^a^	86	0.4 (0.32)	71	0.39 (0.35)	15	0.42 (0.16)	− 0.03 ± 0.06
CCI-all vs. usual care^b^		− 0.0 ± 0.04		0.01 ± 0.05		− 0.06 ± 0.05	
Alkaline phosphatase (µkat L^−1^)							
CCI-all education^a^	256	1.24 (0.37)	200	1.24 (0.37)	56	1.23 (0.36)	0.01 ± 0.05
Usual care^a^	86	1.29 (0.44)	71	1.31 (0.45)	15	1.22 (0.38)	0.09 ± 0.11
CCI-all vs. usual care^b^		− 0.05 ± 0.05		− 0.07 ± 0.06		0.01 ± 0.11	
Serum creatinine (µmol L^−1^)							
CCI-all education^a^	258	77.79 (21.22)	202	77.79 (20.33)	56	81.33 (24.75)	− 3.54 ± 3.54
Usual care^a^	86	80.44 (22.1)	71	78.68 (20.33)	15	86.63 (25.64)	− 7.07 ± 7.07
CCI-all vs. usual care^b^		− 1.77 ± 2.65		− 1.77 ± 2.65		− 5.3 ± 7.07	
BUN (mmol L^−1^)							
CCI-all education^a^	258	6.03 (2.34)	202	6.06 (2.15)	56	5.9 (2.96)	0.16 ± 0.42
Usual care^a^	86	5.73 (2.23)	71	5.59 (1.86)	15	6.38 (3.52)	− 0.79 ± 0.94
CCI-all vs. usual care^b^		0.3 ± 0.28		0.47 ± 0.27		− 0.47 ± 0.99	
eGFR (mL s^−1^ m^−2^)							
CCI-all education^a^	258	1.34 (0.23)	202	1.35 (0.22)	56	1.33 (0.25)	0.02 ± 0.04
Usual care^a^	86	1.32 (0.23)	71	1.34 (0.22)	15	1.26 (0.28)	0.08 ± 0.08
CCI-all vs. usual care^b^		0.02 ± 0.03		0.02 ± 0.03		0.03 ± 0.08	
Anion gap (mmol L^−1^)							
CCI-all education^a^	257	6.83 (1.67)	201	6.79 (1.7)	56	6.98 (1.53)	− 0.19 ± 0.24
Usual care^a^	86	6.93 (1.82)	71	6.92 (1.82)	15	7.0 (1.89)	− 0.08 ± 0.53
CCI-all vs. usual care^b^		− 0.1 ± 0.22		− 0.12 ± 0.25		− 0.02 ± 0.53	
Uric acid (µmo L^−1^)							
CCI-all education^a^	261	347.99 (86.85)	202	348.58 (86.25)	59	346.2 (89.82)	2.38 ± 13.09
Usual care^a^	85	333.12 (87.44)	71	330.74 (85.66)	14	345.01 (98.75)	− 14.28 ± 28.55
CCI-all vs. usual care^b^		14.87 ± 10.71		17.25 ± 11.9		1.19 ± 29.15	
TSH (mIU L^−1^)							
CCI-all education^a^	259	2.32 (1.74)	200	2.31 (1.79)	59	2.38 (1.55)	− 0.07 ± 0.24
Usual care^a^	85	1.97 (1.16)	70	2.09 (1.16)	15	1.38 (1.03)	0.71 ± 0.3*
CCI-all vs. usual care^b^		0.36 ± 0.17*		0.21 ± 0.19		1.0 ± 0.33†	
Free T4 (pmol L^−1^)							
CCI-all education^a^	260	11.84 (2.19)	202	11.84 (2.32)	58	11.58 (2.19)	0.26 ± 0.39
Usual care^a^	86	11.33 (3.73)	71	11.33 (3.86)	15	10.94 (2.32)	0.39 ± 0.77
CCI-all vs. usual care^b^		0.51 ± 0.39		0.51 ± 0.51		0.64 ± 0.64	
Any diabetes medication, excluding metformin (%)							
CCI-all education^a^	262	56.87 ± 3.06	218	55.50 ± 3.37	44	63.64 ± 7.25	− 8.13 ± 8.00
Usual care^a^	87	66.67 ± 5.05	73	68.49 ± 5.44	14	57.14 ± 13.23	11.35 ± 14.32
CCI-all vs. usual care^b^		− 9.80 ± 5.91		− 12.99± 6.39*		6.49 ± 15.08	
Sulfonylurea (%)							
CCI-all education^a^	262	23.66 ± 2.63	218	24.31 ± 2.91	44	20.45 ± 6.08	3.86 ± 6.74
Usual care^a^	87	24.14 ± 4.59	73	23.29 ± 4.95	14	28.57 ± 12.07	− 5.28 ± 13.05
CCI-all vs. usual care^b^		− 0.48 ± 5.29		1.02 ± 5.74		− 8.12± 13.52	
Insulin (%)							
CCI-all education^a^	262	29.77 ± 2.82	218	28.44 ± 3.06	44	36.36 ± 7.25	− 7.92 ± 7.87
Usual care^a^	87	45.98 ± 5.34	78	50.0 ± 5.66	9	11.11 ± 10.48	38.89 (1.91)‡
CCI-all vs. usual care^b^		− 16.21 ± 6.04†		− 21.56 ± 6.43‡		25.25 ± 12.74*	
Thiazolidinedione (%)							
CCI-all education^a^	262	1.53 ± 0.76	218	1.83 ± 0.91	44	0.0 ± 0.0	1.83 ± 0.91*
Usual care^a^	87	1.15 ± 1.14	73	1.37 ± 1.36	14	0.0 ± 0.0	1.37 ± 1.36
CCI-all vs. usual care^b^		0.38 ± 1.37		0.46 ± 1.64		0.0 ± 0.0	
SGLT-2 (%)							
CCI-all education^a^	262	10.31 ± 1.88	218	10.55 ± 2.08	44	9.09 ± 4.33	1.46 ± 4.81
Usual care^a^	87	13.79 ± 3.7	73	15.07 ± 4.19	14	7.14 ± 6.88	7.93 ± 8.06
CCI-all vs. usual care^b^		− 3.48 ± 4.15		− 4.52 ± 4.68		1.95 ± 8.13*	
DPP-4 (%)							
CCI-all education^a^	262	9.92 ± 1.85	218	10.09 ± 2.04	44	9.09 ± 4.33	1.0 ± 4.79
Usual care^a^	87	8.05 ± 2.92	73	8.22 ± 3.21	14	7.14 ± 6.88	1.08 ± 7.60
CCI-all vs. usual care^b^		1.87 ± 3.45		1.87 ± 3.81		1.95 ± 8.13	
GLP-1 (%)							
CCI-all education^a^	262	13.36 ± 2.1	218	12.84 ± 2.27	44	15.91 ± 5.51	− 3.07 ± 5.96
Usual care^a^	87	14.94 ± 3.82	73	16.44 ± 4.34	14	7.14 ± 6.88	9.30 ± 8.14
CCI-all vs. usual care^b^		− 1.58 ± 4.36		− 3.59 ± 4.89		8.77 ± 8.82	
Metformin (%)							
CCI-all education^a^	262	71.37 ± 2.79	218	71.56 ± 3.06	44	70.45 ± 6.88	1.11 ± 7.53
Usual care^a^	87	60.92 ± 5.23	73	61.64 ± 5.69	14	57.14 ± 13.23	4.50 ± 14.40
CCI-all vs. usual care^b^		10.45 ± 5.93		9.92 ± 6.46		13.31 ± 14.91	

See Table S1 (electronic supplemental material) for CCI-web, CCI-onsite, and additional comparisons

^a^Mean and standard deviations for continuous variables, percentages and standard errors for categorical variables

^b^Difference between means or percentages ± 1 standard error of the difference. Significant baseline difference between means or percentages at 0.05 > *P* ≥ 0.01 (*); 0.01 > *P* ≥ 0.001 (†); 0.001 > *P* ≥ 0.0001 (‡); and *P* < 0.0001 (§)

**Table 2:** Mean changes in biomarkers between baseline and 1-year for participants receiving the CCI and UC

	Completers						All starters (dropouts imputed)^d^		
	Unadjusted				Adjusted for baseline^c^		Unadjusted		
	N	Difference (SD) or ± SE	1 Year	Significance^e^	Difference ±SE	Signif-icance^e^	Difference ± SE	1 Year	Significance^e^
Beta-hydroxybutyrate (mmol L^−1^)									
CCI-all education^a^	186	0.14 (0.36)	0.31 (0.35)	2.2 × 10^−5^	0.13 ± 0.02	2.8 × 10^−7^	0.12 ± 0.02	0.3 ± 0.02	5.8 × 10^−7^
Usual care^a^	59	0.04 (0.23)	0.18 (0.21)	0.24	0.06 ± 0.05	0.18	0.03 ± 0.04	0.18 ± 0.03	0.38
CCI-all vs. usual care^b^		0.1 (0.0)		0.01	0.06 ± 0.05	0.24	0.09 ± 0.04		0.04
Hemoglobin A_1c_ (mmol mol^−1^)									
CCI-all education^a^	204	− 14.1 (14.43)	44.25 (10.28)	<10^−16^	− 14.43 ± 0.98	<10^−16^	− 14.21 ± 0.98	45.23 ± 0.77	<10^−16^
Usual care^a^	72	2.19 (14.76)	63.27 (19.89)	0.21	2.4 ± 1.75	0.17	2.19 ± 1.64	62.18 ± 2.08	0.18
CCI-all vs. usual care^b^		− 16.29 ± 1.97		4.4 × 10^−16^	− 16.83 ± 2.08	4.4 × 10^−16^	− 16.4 ± 1.86		<10^−16^
Hemoglobin A_1c_ (%)									
CCI-all education^a^	204	− 1.29 (1.32)	6.20 (0.94)	<10^−16^	− 1.32 ± 0.09	<10^−16^	− 1.30 ± 0.09	6.29 ± 0.07	< 10^−16^
Usual care^a^	72	0.20 (1.35)	7.94 (1.82)	0.21	0.22 ± 0.16	0.17	0.20 ± 0.15	7.84 ± 0.19	0.18
CCI-all vs. usual care^b^		− 1.49 ± 0.18		4.4 × 10^−16^	− 1.54 ± 0.19	4.4 × 10^−16^	−1.50 ± 0.17		< 10^−16^
Fasting glucose (mmol L^−1^)									
CCI-all education^a^	202	− 1.96 (3.2)	6.84 (1.87)	<10^−16^	− 2.02 ± 0.26	6.0 × 10^−15^	− 1.95 ± 0.23	6.98 ± 0.17	< 10^−16^
Usual care^a^	71	0.59 (4.59)	9.3 (4.74)	0.28	0.81 ± 0.45	0.07	0.63 ± 0.49	9.29 ± 0.49	0.2
CCI-all vs. usual care^b^		− 2.55 ± 0.59		1.5 × 10^−5^	− 2.83 ± 0.53	7.9 × 10^−8^	− 2.58 ± 0.54		2.1 × 10^−6^
Insulin, all (pmol L^−1^)									
CCI-all education^a^	186	− 75.01 (178.49)	122.58 (169.6)	9.9 × 10^−9^	− 91.4 ± 12.15	5.5 × 10^−14^	− 73.62 ± 12.5	126.26 ± 12.5	4.3 × 10^−9^
Usual care^a^	59	12.15 (210.23)	218.91 (239.46)	0.66	36.88 ± 29.66	0.21	5.97 ± 24.52	206.27 ± 26.11	0.81
CCI-all vs. usual care^b^		− 87.23 (29.86)		0.004	− 127.58 ± 32.43	0.0009	− 79.59 ± 27.5		0.004
C-peptide (nmol L^−1^)									
CCI-all education^a^	185	− 0.36 (0.57)	1.11 (0.59)	<10^−16^	− 0.34 ± 0.05	1.1 × 10^−13^	− 0.33 ± 0.04	1.11 ± 0.04	2.2 × 10^−16^
Usual care^a^	59	0.08 (0.77)	1.43 (0.92)	0.41	0.02 ± 0.09	0.79	0.06 ± 0.09	1.44 ± 0.1	0.5
CCI-all vs. usual care^b^		− 0.44 ± 0.11		5.4 × 10^−5^	− 0.37 ± 0.1	0.0004	− 0.4 ± 0.1		5.3 × 10^−5^
HOMA-IR (insulin derived), all									
CCI-all education^a^	179	− 5.54 (12.19)	5.65 (8.71)	1.2 × 10^−9^	− 5.87 ± 0.92	2.2 × 10^−10^	− 5.58 ± 0.86	6.16 ± 0.69	7.5 × 10^−11^
Usual care^a^	56	1.65 (12.46)	12.96 (12.9)	0.32	2.4 ± 1.76	0.17	1.82 ± 1.49	12.2 ± 1.42	0.22
CCI-all vs. usual care^b^		− 7.19 (1.9)		0.0002	− 8.27±2.04	4.9 × 10^−5^	− 7.4 ± 1.72		1.6 × 10^−5^
HOMA^-^IR (insulin derived), e × cluding e × ogenous insulin users									
CCI-all education^a^	129	− 6.03 (10.67)	4.98 (5.69)	1.4 × 10^−10^	− 6.13 ± 0.98	4.2 × 10^−10^	− 6.82 ± 0.9	5.61 ± 0.51	3.2 × 10^−5^
Usual care^a^	25	3.99 (12.76)	13.35 (14.71)	0.12	4.1 ± 2.34	0.08	1.84 ± 1.96	13.3 ± 1.56	0.35
CCI-all vs. usual care^b^		− 10.01 ± 2.72		0.0002	− 10.23 ± 2.56	6.3 × 10^−5^	− 8.65 ± 2.16		6.0 × 10^−5^
HOMA-IR (C-peptide derived)									
CCI-all education^a^	170	− 3.53 (5.59)	7.9 (3.89)	2.2 × 10^−16^	− 3.53 ± 0.55	1.2 × 10^−10^	− 3.45 ± 0.46	8.25 ± 0.4	1.0 × 10^−13^
Usual care^a^	47	1.94 (10.54)	12.49 (10.46)	0.21	1.77 ± 1.12	0.11	1.65 ± 1.13	12.6 ± 1.11	0.14
CCI-all vs. usual care^b^		− 5.47 (1.6)		0.0006	− 5.29 ± 1.28	3.3 × 10^−5^	− 5.11 ± 1.22		3.0 × 10^−5^
Weight-clinic (kg)									
CCI-all education^a^	184	− 14.24 (10.29)	101.17 (22.06)	<10^−16^	− 13.81 ± 0.63	<10^−16^	− 13.8 ± 0.71	102.72 ± 1.5	< 10^−16^
Usual care^a^	69	0.04 (5.94)	106.82 (22.52)	0.95	− 1.11 ± 1.06	0.29	− 0.16 ± 0.84	107.31 ± 2.55	0.85
CCI-all vs. usual care^b^		− 14.29 ± 1.04		<10^−16^	− 12.7 ± 1.26	<10^−16^	− 13.65 ± 1.1		< 10^−16^
Systolic blood pressure (mmHg)									
CCI-all education^a^	187	− 6.77 (16.3)	125.84 (13.22)	1.3 × 10^−8^	− 6.52 ± 1.24	1.6 × 10^−7^	− 6.36 ± 1.12	125.57 ± 0.91	1.3 × 10^−8^
Usual care^a^	67	0.25 (17.8)	128.57 (11.82)	0.91	− 0.45 ± 2.15	0.83	− 0.9 ± 2.07	129.01 ± 1.72	0.67
CCI-all vs. usual care^b^		− 7.02 (2.4)		0.005	− 6.07 ± 2.55	0.02	− 5.46 ± 2.36		0.02
Diastolic blood pressure (mmHg)									
CCI-all education^a^	187	− 3.59 (9.33)	78.0 (7.55)	1.4 × 10^−7^	− 3.5 ± 0.7	6.2 × 10^−7^	− 3.51 ± 0.65	78.58 ± 0.56	7.2 × 10^−8^
Usual care^a^	67	− 0.12 (10.15)	80.99 (9.59)	0.92	− 0.39 ± 1.21	0.75	− 0.9 ± 1.2	81.12 ± 1.08	0.45
CCI-all vs. usual care^b^		− 3.47 ± 1.42		0.01	− 3.10±1.44	0.03	− 2.61 ± 1.37		0.06
Total cholesterol (mmol L^−1^)									
CCI-all education^a^	186	0.24 (0.93)	4.92 (1.18)	0.0004	0.24 ± 0.08	0.004	0.21 ± 0.07	4.97 ± 0.08	0.006
Usual care^a^	59	0.00 (1.60)	4.72 (1.62)	0.99	0.0 ± 0.16	0.98	− 0.04 ± 0.18	4.69 ± 0.18	0.83
CCI-all vs. usual care^b^		0.25 ± 0.22		0.26	0.25 ± 0.18	0.17	0.24 ± 0.19		0.2
LDL-C (mmol^−1^)									
CCI-all education^a^	172	0.28 (0.83)	2.87 (0.98)	7.7 × 10^−6^	0.28 ± 0.07	2.6 × 10^−5^	0.26 ± 0.06	2.94 ± 0.07	5.1 × 10^−5^
Usual care^a^	48	− 0.28 (0.97)	2.32 (0.8)	0.05	−0.28 ± 0.13	0.03	− 0.28 ± 0.12	2.32 ± 0.12	0.02
CCI-all vs. usual care^b^		0.56 ± 0.15		0.0003	0.56 ± 0.15	0.0002	0.54 ± 0.14		0.0001
Apo B (g L^−1^)									
CCI-all education^a^	186	− 0.01 (0.24)	1.03 (0.29)	0.69	− 0.0 ± 0.02	0.82	− 0.02 ± 0.02	1.04 ± 0.02	0.37
Usual care^a^	59	0.02 (0.37)	1.07 (0.39)	0.75	0.0 ± 0.04	0.9	0.0 ± 0.04	1.06 ± 0.04	0.95
CCI-all vs. usual care^b^		− 0.02 (0.05)		0.66	− 0.01 ± 0.05	0.83	− 0.02 ± 0.05		0.67
HDL-C (mmol L^−1^)									
CCI-all education^a^	186	0.20 (0.31)	1.29 (0.41)	<10^−16^	0.19 ± 0.02	<10^−16^	0.2 ± 0.02	1.29 ± 0.03	< 10^−16^
Usual care^a^	59	− 0.04 (0.23)	0.92 (0.32)	0.15	− 0.02 ± 0.04	0.69	− 0.03 ± 0.03	0.95 ± 0.04	0.41
CCI-all vs. usual care^b^		0.24 ± 0.04		1.7 × 10^−10^	0.2 ± 0.05	9.9 × 10^−6^	0.23 ± 0.04		1.3 × 10^−8^
Triglycerides (mmol L^−1^)									
CCI-all education^a^	186	− 0.56 (1.9)	1.71 (1.64)	<10^−16^	− 0.56 ± 0.18	9.3 × 10^−15^	− 0.54 ± 0.14	1.67 ± 0.13	< 10^−16^
Usual care^a^	59	0.34 (3.40)	3.7 (5.67)	0.22	− 0.35 ± 0.32	0.48	0.32 ± 0.37	3.45 ± 0.55	0.43
CCI-all vs. usual care^b^		− 0.9 ± 0.46		1.4 × 10^−7^	− 0.92 ± 0.38	7.5 × 10^−6^	− 0.86 ± 0.39		9.9 × 10^−7^
Total/HDL-cholesterol									
CCI-all education^a^	186	− 0.47 (1.41)	4.18 (1.71)	4.1 × 10^−6^	− 0.45 ± 0.16	0.005	− 0.53 ± 0.12	4.19 ± 0.13	1.7 × 10^−5^
Usual care^a^	59	0.52 (3.45)	5.96 (4.27)	0.24	0.44 ± 0.29	0.13	0.42 ± 0.36	5.73 ± 0.43	0.24
CCI-all vs. usual care^b^		− 1.00 ± 0.46		0.03	− 0.89 ± 0.33	0.008	− 0.95 ± 0.38		0.01
hsC^−^reactive protein (nmol L^−1^)									
CCI-all education^a^	193	− 31.71 (127.15)	53.81 (67.24)	<10^−8^	− 29.43 ± 9.14	<10^−16^	− 34.29 ± 10.0	52.86 ± 5.24	< 10^−16^
Usual care^a^	70	12.48 (126.86)	99.43 (139.91)	0.94	8.48 ± 16.1	0.88	12.48 ± 14.1	97.72 ± 14.76	0.93
CCI-all vs. usual care^b^		− 44.29 (17.14)		1.2 × 10^−6^	− 37.91 ± 18.55	3.0 × 10^−5^	− 46.76 ± 17.24		9.3 × 10^−7^
ALT (µkat L^−1^)									
CCI-all education^a^	201	− 0.16 (0.4)	0.36 (0.19)	9.5 × 10^−9^	− 0.16 ± 0.03	9.4 × 10^−10^	− 0.15 ± 0.02	0.36 ± 0.01	2.4 × 10^−10^
Usual care^a^	71	0.01 (0.28)	0.47 (0.34)	0.67	0.02 ± 0.05	0.67	0.01 ± 0.03	0.47 ± 0.04	0.77
CCI-all vs. usual care^b^		− 0.18 (0.04)		5.1 × 10^−5^	− 0.18 ± 0.05	0.0009	− 0.16 ± 0.04		4.6 × 10^−5^
AST (µkat L^−1^)									
CCI-all education^a^	201	− 0.09 (0.27)	0.32 (0.11)	2.8 × 10^−6^	− 0.09 ± 0.02	1.3 × 10^−5^	− 0.08 ± 0.02	0.32 ± 0.01	5.1 × 10^−7^
Usual care^a^	71	0.01 (0.32)	0.4 (0.27)	0.79	0.01 ± 0.04	0.69	0.01 ± 0.03	0.41 ± 0.03	0.72
CCI-all vs. usual care^b^		− 0.1 (0.04)		0.02	− 0.1 ± 0.04	0.02	− 0.09 ± 0.04		0.01
Alkaline phosphatase (µkat L^−1^)									
CCI-all education^a^	200	− 0.16 (0.24)	1.07 (0.35)	<10^−8^	− 0.17 ± 0.02	<10^−16^	− 0.16 ± 0.02	1.08 ± 0.02	< 10^−16^
Usual care^a^	71	0.0 (0.22)	1.31 (0.45)	0.94	0.02 ± 0.03	0.61	0.01 ± 0.03	1.3 ± 0.05	0.67
CCI-all vs. usual care^b^		− 0.17 (0.03)		6.3 × 10^−8^	− 0.18 ± 0.03	1.4 × 10^−7^	− 0.17 ± 0.03		3.1 × 10^−8^
Serum creatinine (µmol L^−1^)									
CCI-all education^a^	202	− 3.54 (14.14)	73.37 (18.56)	0.0001	− 3.54 ± 0.88	0.001	− 3.54 ± 0.88	74.26 ± 0.88	0.0001
Usual care^a^	71	− 0.88 (18.56)	77.79 (18.56)	0.56	− 2.65 ± 1.77	0.15	− 1.77 ± 1.77	78.68 ± 1.77	0.29
CCI-all vs. usual care^b^		− 2.65 ± 2.65		0.32	− 0.88 ± 2.65	0.73	− 1.77 ± 2.65		0.49
BUN (mmol L^−1^)									
CCI-all education^a^	202	0.76 (2.49)	6.82 (2.78)	1.5 × 10^−5^	0.75 ± 0.17	1.6 × 10^−5^	0.79 ± 0.17	6.81 ± 0.19	5.5 × 10^−6^
Usual care^a^	71	0.07 (2.15)	5.66 (2.11)	0.78	0.06 ± 0.3	0.85	− 0.01 ± 0.27	5.72 ± 0.26	0.97
CCI-all vs. usual care^b^		0.69 (0.29)		0.03	0.69 (0.32)	0.05	0.8 ± 0.32		0.01
eGFR (mL s^−1^ m^−2^)									
CCI-all education^a^	202	0.03 (0.15)	1.38 (0.2)	0.003	0.03 ± 0.01	0.009	0.03 ± 0.01	1.38 ± 0.01	0.005
Usual care^a^	71	0.01 (0.19)	1.34 (0.22)	0.51	0.02 ± 0.02	0.36	0.01 ± 0.02	1.34 ± 0.02	0.48
CCI-all vs. usual care^b^		0.03 ± 0.02		0.28	0.01 ± 0.02	0.61	0.02 ± 0.02		0.51
Anion gap (mmol L^−1^)									
CCI-all education^a^	201	0.29 (2.02)	7.08 (1.75)	0.04	0.28 ± 0.15	0.06	0.28 ± 0.14	7.13 ± 0.12	0.04
Usual care^a^	71	0.83 (2.44)	7.75 (1.97)	0.004	0.84 ± 0.26	0.001	0.81 ± 0.26	7.75 ± 0.22	0.002
CCI-all vs. usual care^b^		− 0.54 (0.3)		0.09	− 0.56 ± 0.31	0.07	− 0.53 ± 0.3		0.08
CO_2_ (mmol L^−1^)									
CCI-all education^a^	202	0.12 (2.26)	28.06 (2.29)	0.45	0.16 ± 0.18	0.38	0.18 ± 0.16	27.94 ± 0.16	0.27
Usual care^a^	71	0.17 (2.97)	28.23 (2.58)	0.63	− 0.2 ± 0.3	0.51	0.04 ± 0.33	28.12 ± 0.29	0.9
CCI-all vs. usual care^b^		− 0.05 (0.3)		0.9	0.36 ± 0.36	0.32	0.14 ± 0.37		0.71
Uric acid (µmol L^−1^)									
CCI-all education^a^	202	0.59 (70.79)	349.18 (91.01)	0.91	1.78 ± 4.76	0.72	1.78 ± 4.76	349.77 ± 5.95	0.67
Usual care^a^	71	− 10.71 (64.24)	320.03 (85.06)	0.15	− 16.66 ± 8.92	0.06	− 11.9 ± 7.73	322.41 ± 10.11	0.13
CCI-all vs. usual care^b^		11.3 ± 8.92		0.21	18.44 ± 10.11	0.07	13.68 ± 8.92		0.13
TSH (mIU L^−1^)									
CCI-all education^a^	200	− 0.41 (1.62)	1.9 (1.1)	0.0004	− 0.4 ± 0.11	0.0002	− 0.42 ± 0.1	1.91 ± 0.08	5.3 × 10^−5^
Usual care^a^	70	− 0.09 (0.99)	2.01 (0.99)	0.47	− 0.09 ± 0.19	0.64	0.0 ± 0.12	1.96 ± 0.12	0.98
CCI-all vs. usual care^b^		− 0.33 ± 0.16		0.05	− 0.31 ± 0.22	0.15	− 0.42 ± 0.16		0.01
Free T4 (pmol L^−1^)									
CCI-all education^a^	202	0.13 (2.32)	11.97 (2.45)	0.7	0.0 ± 0.26	0.83	0.13 ± 0.13	11.97 ± 0.13	0.58
Usual care^a^	71	0.26 (4.25)	11.58 (2.83)	0.7	0.26 ± 0.39	0.48	0.26 ± 0.39	11.46 ± 0.26	0.61
CCI-all vs. usual care^b^		− 0.13 (0.0)		0.8	− 0.26 ± 0.39	0.43	− 0.13 ± 0.51		0.78
Any diabetes medication, e × cluding metformin (%)									
CCI-all education^a^	218	− 27.52 (49.65)	27.98 ± 3.05	2.2 × 10^−16^	− 27.66 ± 3.21	<10^−16^	− 27.19 ± 3.14	29.68 ± 2.94	< 10^−16^
Usual care^a^	78	6.85 (34.68)	75.34 ± 5.08	0.09	7.54 ± 5.87	0.2	5.99 ± 4.31	72.66 ± 5.0	0.09
CCI-all vs. usual care^b^		− 34.37 ± 5.27		7.0 × 10^−11^	^−^ 35.36 ± 6.83	2.3 × 10^−7^	− 33.19 ± 5.34		9.0 × 10^−9^
Sulfonylurea (%)									
CCI-all education^a^	218	− 24.31 (43.0)	0.0 ± 0.0	<10^−16^	− 24.23 ± 2.86	<10^−16^	− 23.67 ± 2.7	0.0 ± 0.0	< 10^−16^
Usual care^a^	78	2.74 (37.17)	26.02 ± 5.17	0.53	2.56 ± 5.24	0.63	1.91 ± 4.23	26.02 ± 5.17	0.65
CCI-all vs. usual care^b^		− 27.05 ± 5.23		2.4 × 10^−7^	− 26.85 ± 6.07	9.7 × 10^−6^	− 25.58 ± 5.02		3.3 × 10^−7^
Insulin (%)									
CCI-all education^a^	218	− 13.3 (35.37)	15.14 ± 2.43	2.8 × 10^−8^	− 15.5 ± 2.0	9.3 × 10^−15^	− 13.03 ± 2.22	16.74 ± 2.4	4.3 × 10^−9^
Usual care^a^	78	1.37 (31.15)	52.05 ± 5.89	0.71	8.46 ± 3.65	0.02	3.17 ± 3.68	49.18 ± 5.45	0.39
CCI-all vs. usual care^b^		− 14.67 ± 4.36		0.0008	− 23.89 ± 4.24	1.8 × 10^−8^	− 16.19 ± 4.3		0.0002
Thiazolidinedione (%)									
CCI-all education^a^	218	− 1.38 (15.12)	0.46 ± 0.46	0.18	− 1.47 ± 0.9	0.1	− 1.1 ± 0.91	0.42 ± 0.49	0.23
Usual care^a^	78	0.0 (0.0)	1.37 ± 1.37		0.26 ± 1.64	0.87	0.22 ± 0.51	1.27 ± 1.26	0.67
CCI-all vs. usual care^b^		− 1.38 ± 1.02		0.18	− 1.78 ± 1.91	0.35	− 1.31 ± 1.04		0.21
SGLT^−^2 (%)									
CCI-all education^a^	218	− 9.63 (29.57)	0.92 ± 0.65	1.5 × 10^-6^	− 9.96 ± 2.04	1.1 × 10^−6^	− 9.26 ± 1.88	0.92 ± 1.88	9.0 × 10^−7^
Usual care^a^	78	0.0 (28.87)	15.07 ± 4.22	1	1.13 ± 3.72	0.76	0.87 ± 3.17	15.07 ± 4.21	0.78
CCI-all vs. usual care^b^		− 9.63 ± 3.93		0.01	− 11.0 ± 4.32	0.01	− 10.13 ± 3.69		0.006
DPP^−^4 (%)									
CCI-all education^a^	218	− 3.67 (34.42)	6.42 ± 1.66	0.12	− 3.69 ± 2.21	0.09	− 3.52 ± 2.21	6.29 ± 1.66	0.11
Usual care^a^	78	2.74 (23.41)	10.96 ± 3.68	0.32	2.97 ± 4.05	0.46	2.64 ± 2.92	10.74 ± 3.51	0.37
CCI-all vs. usual care^b^		− 6.41 ± 3.6		0.07	− 6.64 ± 4.7	0.16	− 6.16 ± 3.66		0.09
GLP^−^1 (%)									
CCI-all education^a^	218	0.92 (34.6)	13.76 ± 2.34	0.7	1.15 ± 2.31	0.62	0.98 ± 2.3	14.4 ± 2.29	0.67
Usual care^a^	78	2.74 (33.22)	19.18 ± 4.64	0.48	2.09 ± 4.23	0.62	2.94 ± 3.84	17.02 ± 4.39	0.44
CCI-all vs. usual care^b^		− 1.82 ± 4.54		0.69	− 0.99 ± 4.91	0.84	− 1.96 ± 4.48		0.66
Metformin (%)									
CCI-all education^a^	218	− 7.34 (46.45)	64.22 ± 3.25	0.02	− 7.14 ± 3.0	0.02	− 6.34 ± 3.06	65.18 ± 3.14	0.04
Usual care^a^	78	0.0 (37.27)	61.64 ± 5.73	1	0.83 ± 5.5	0.88	− 0.08 ± 4.55	60.67 ± 5.61	0.99
CCI-all vs. usual care^b^		− 7.34 ± 5.38		0.17	− 7.95 ± 6.38	0.21	− 6.26 ± 5.48		0.25

See Table S2 (electronic supplemental material) for CCI-web, CCI-onsite, and additional comparisons

^a^Imputed values based on 700 iterations from multivariate normal regression

^b^Adjusted for sex, age, baseline BMI, baseline insulin use (user vs. non-user), and African-American race

^c^A significance level of *P* < 0.0017 ensures overall simultaneous significance of *P* ≤ 0.05 over the 30 variables using Bonferroni correction

^d^Means (standard deviations) are presented. Sample sizes, means, and significance levels refer to subjects with baseline and 1-year measurements for completers, and to 349 subjects (262 intervention and 87 usual care) for all starters. Significance levels for completers refer to one-sample* t* test with or without adjustment. Untransformed triglyceride and hsC-reactive protein values are presented; however, their statistical significances were based on their log-transformed values. CCI-all refers to the CCI-web and CCI-onsite combined

^e^Mean differences ± one standard error. Significance levels refer to two-sample* t* test or analysis of covariance for the differences

## Results

### Participant Characteristics

Table 1 presents baseline characteristics of the 262 CCI and 87 UC participants. At baseline, 88% of CCI participants were prescribed diabetes medication (57% were prescribed a diabetes medication other than metformin, 30% prescribed insulin) and 93% were obese. Eighty-seven percent of participants in UC at baseline were prescribed diabetes medication (46% prescribed insulin), and 82% were obese. Forty-four participants (16.8%) withdrew from the CCI, 22 from each education delivery mode. Baseline characteristics of CCI dropouts did not differ significantly from the 218 completers except none of the five thiazolidinedione users were dropouts (Table 1). At baseline, characteristics of CCI participants who self-selected web-based versus on-site education were not significantly different after accounting for multiple comparisons (see Table S1 in the electronic supplementary material). Compared to the 78 UC participants who completed the study, the nine that withdrew tended to be older (58 versus 52 years old), had lower TSH, and fewer were prescribed insulin, SGLT-2, DPP-4, GLP-1, or blood pressure medications (Table 1).

### Effectiveness

**Fig. 1:**
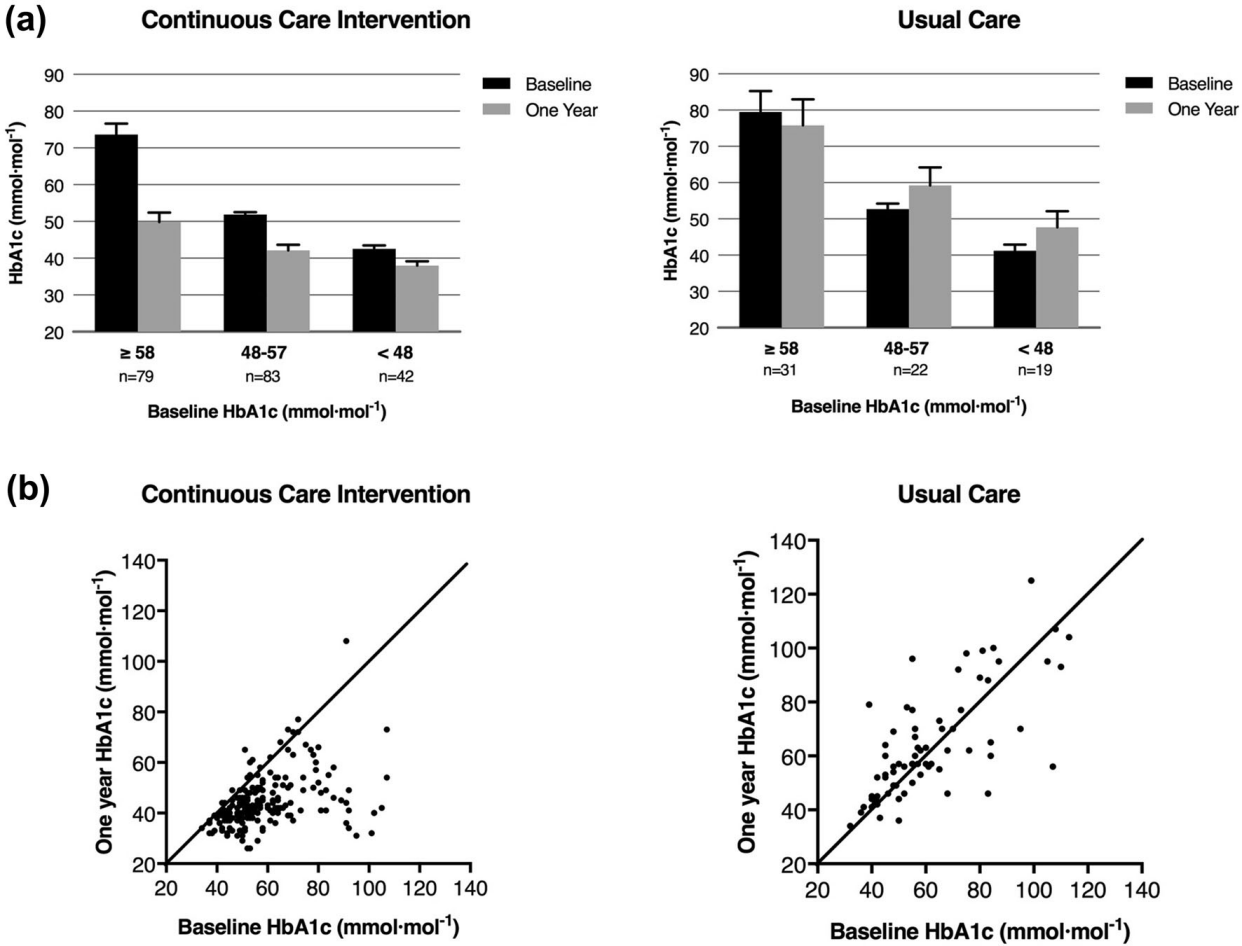
Change in HbA_1c_ over the course of 1 year for CCI and UC groups. **a** Mean (95% CI) in HbA_1c_ based on starting value at baseline and 1 year for completers in both groups. **b** Individual changes in HbA_1c_ over 1 year for completers in both groups

**Fig. 2:**
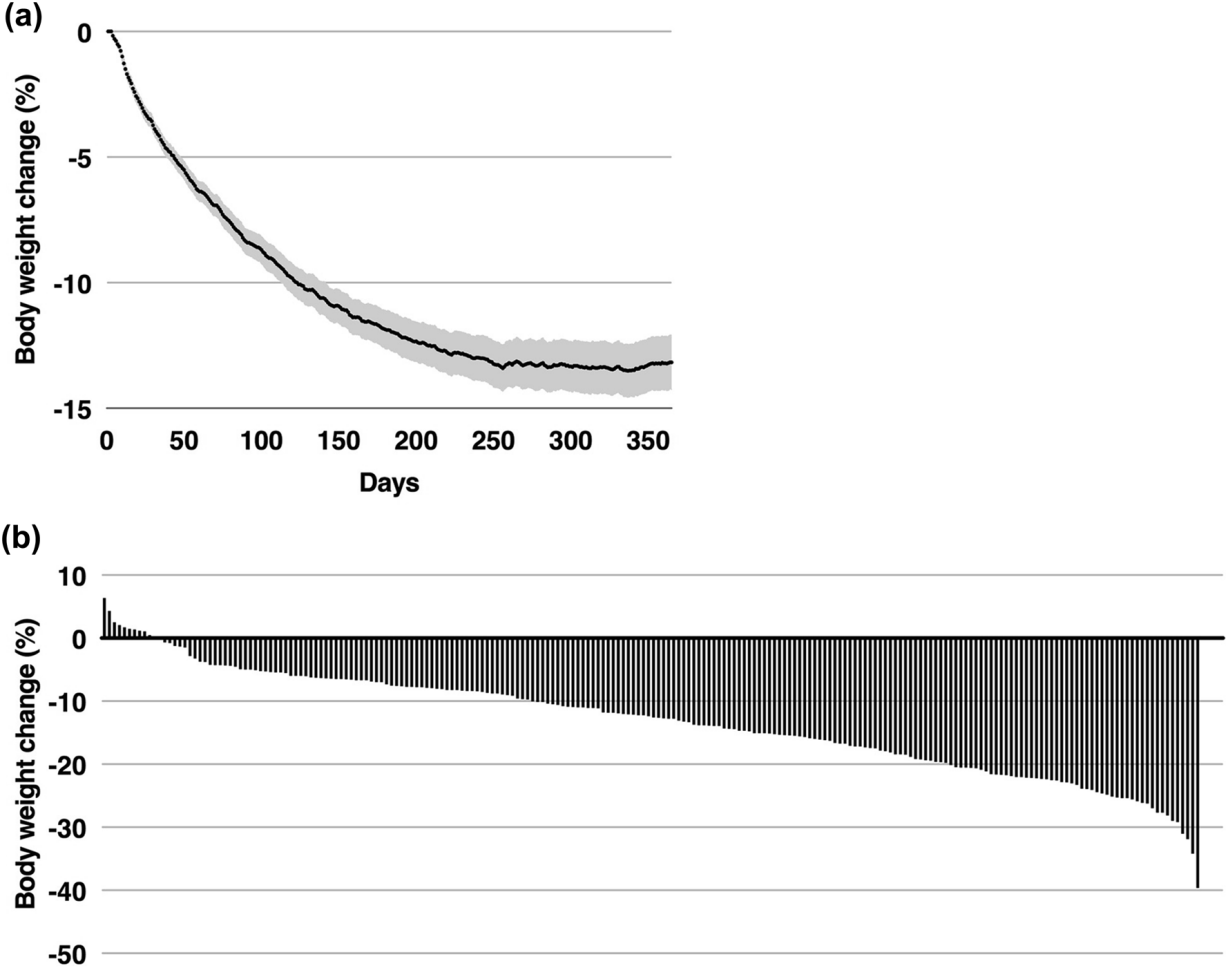
Body weight change over the course of 1 year in CCI completers. **a** Mean (95% CI) change in body weight for completers over the course of 1 year. For each individual, weight on a given day was computed as the 3-day trailing mean (to reduce day-to-day variation). On dates where no weights were recorded during the 3-day time window for a given participant, the most recent 3-day mean preceding the date was used. **b** Histogram depicting individual body weight changes at 1 year

Table 2 presents mean 1-year changes in biomarkers. In the CCI, HbA_1c_ was significantly reduced 17%, from 60 ± 1.0 mmol mol^−1^ (7.6 ± 0.09%) at baseline to 45 ± 0.8 mmol mol^−1^ (6.3 ± 0.07%) after 1 year (nominal significance *P* < 1.0 × 10^−16^; Fig. 1). Eighty-five percent (174/204) of CCI participants completing 1-year HbA_1c_ testing observed a decline greater than 2.2 mmol mol^−1^ (> 0.2%) in the measure. When adjusted for multiple comparisons, significant within-CCI reductions were observed in fasting glucose (− 22%, *P* < 1.0 × 10^−16^), fasting insulin (− 43%, *P* = 6.7 × 10^−16^), C-peptide (− 23%, *P* = 2.2 × 10^−16^), HOMA-IR derived from fasting insulin excluding exogenous users (− 55%, *P* = 3.2 × 10^−5^), HOMA-IR derived from C-peptide (− 29%, *P* = 1.0 × 10^−13^), weight from clinic measurements (− 12%, *P* < 1.0 × 10^−16^), weight from home scales (− 13%, *P* < 1.0 × 10^−16^, Fig. 2), triglycerides (− 24%, *P* < 1.0 × 10^−16^), high-sensitivity C-reactive protein (− 39%, *P* < 1.0 × 10^−16^), ALT (− 30%, *P* = 2.4 × 10^−10^), AST (− 21%, *P* = 5.1 × 10^−7^), and alkaline phosphatase (− 13%, *P* < 1.0 × 10^−16^). HDL-cholesterol increased 18% (*P* < 1.0 × 10^−16^) and calculated LDL-cholesterol increased 10% (*P* = 5.1 × 10^−5^) while apolipoprotein B (ApoB) concentration was unchanged (*P* = 0.37) for participants in the CCI. There were no significant differences in mean biomarker changes between CCI-web and CCI-onsite (see Table S2 in the electronic supplementary material). In contrast to the CCI, patients enrolled in UC for 1 year showed no Bonferroni-adjusted significant change for any of the biomarkers measured (Table 2).

**Fig. 3:**
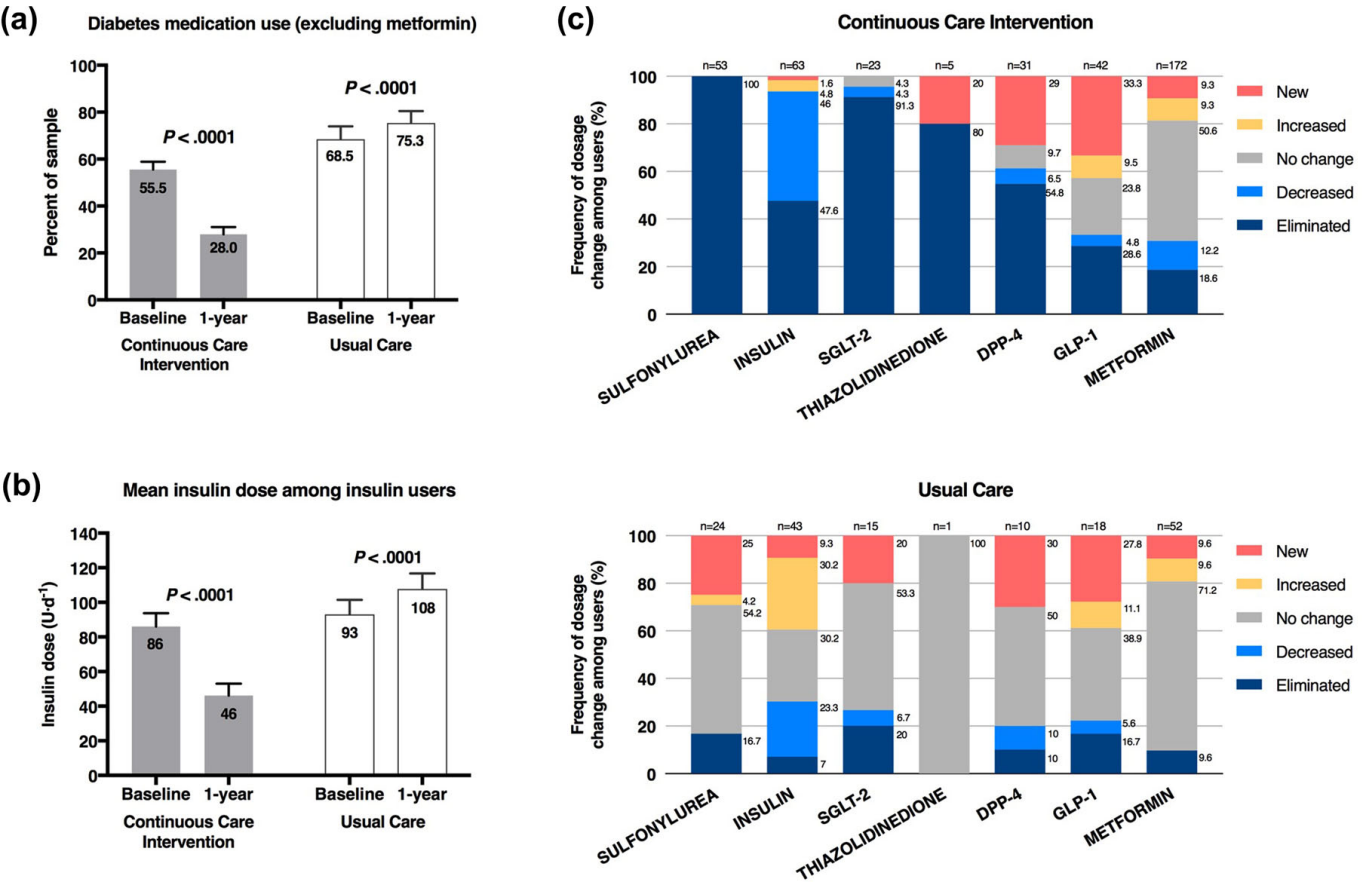
Medication changes over the course of 1 year in completers of the CCI and UC groups. **a** Proportion of completers prescribed diabetes medications other than metformin. **b** Mean ± SE prescribed dose among insulin users. **c** Frequency in change of medication dosage among prescribed users by diabetes medication class in both groups

Following 1 year of CCI, usage of all diabetes medications combined (excluding metformin) was reduced significantly (56.9 ± 3.1% to 29.7 ± 3.0%, *P* < 1.0 × 10^−16^) through decreased prescriptions for DPP-4 (9.9–6.3%, *P* = 0.11), insulin (29.8–16.7%, *P* = 4.3 × 10^−9^), SGLT-2 inhibitors (10.3–0.9%, *P* = 9 × 10^−7^), sulfonylureas (23.7–0%, *P* < 1.0 × 10^−16^), and thiazolidinediones (1.5–0.4%, *P* = 0.23) (Fig. 3). GLP-1 prescriptions were statistically unchanged (13.4% at baseline to 14.4% at 1 year, *P* = 0.67), and metformin decreased slightly (71.4–65.0%, *P* = 0.04) for CCI participants. Forty percent (31/78) of CCI participants who began the study with insulin prescriptions (average dose of 64.2 units) eliminated the medication, while the remaining 60% (47/78) of insulin users reduced daily dosage from 105.2 to 53.8 units (*P* < 0.0001). Patients enrolled in UC for 1 year showed no Bonferroni-adjusted significant change for prescription of medication. For the 34 UC participants that continued using insulin, the average daily dose increased from 96.0 to 111.9 units.

The proportion of participants in the total imputed CCI group with HbA_1c_below 48 mmol mol^−1^ (< 6.5%) increased from 19.5 ± 2.4% to 69.8 ± 3.1%. Of those in the CCI with HbA_1c_ reported at 1 year, 72% (147/204) achieved HbA_1c_ below 48 mmol mol^−1^ (6.5%) and 60.3% (123/204) of participants achieved HbA_1c_ below 48 mmol mol^−1^ (< 6.5%) while taking no diabetes medication or only metformin. Of those in the CCI with HbA_1c_ below 48 mmol mol^−1^ (< 6.5%) at 1 year, 42.3% (52/123) were prescribed no diabetes medication and 57.7% (71/123) were prescribed metformin only. The proportion of the total imputed CCI group with fasting glucose below 6.99 mmol L^−1^ at 1 year increased from 34.9 ± 3.3% to 58.4 ± 3.9%, and the proportion with class III obesity decreased from 45.5 ± 3.1% to 19.6 ± 2.8%.

Compared to UC, the CCI showed significant Bonferroni-adjusted (*P* < 0.0017) net reductions in HbA_1c_ (nominal significance for the two-group comparison, *P* < 10^−16^; Fig. 1), fasting glucose (*P* = 2.1 × 10^−6^), fasting insulin excluding exogenous users (*P* = 4.6 × 10^−5^), C-peptide (*P* = 5.3 × 10^−5^), HOMA-IR derived from insulin excluding exogenous users (*P* = 6.0 × 10^−5^) or derived from C-peptide (*P* = 3.0 × 10^−5^), weight (*P* < 10^−16^), triglycerides (*P* = 1.0 × 10^−6^), hsCRP (*P* = 9.3 × 10^−7^), ALT (*P* = 4.6 × 10^−5^), and alkaline phosphatase (*P* = 3.1 × 10^−8^). All of these group differences remained significant when adjusted for the baseline age, sex, insulin medication use, and body mass index (Table 2). The CCI decrease in diabetes medication use was significantly greater than the changes in the UC group for all diabetes medications (*P* < 10^−16^) and all diabetes medications excluding metformin (*P* = 9.0 × 10^−9^), including sulfonylurea (*P* = 3.3 × 10^−7^) and insulin (*P* = 0.0002) (Fig. 3).

The CCI-web and CC-onsite sub-cohorts provide replication of the above results. Specifically, Table S2 (see electronic supplementary material) shows that within-group Bonferroni significance was achieved separately for the mean 1-year reductions in HbA_1c_, fasting glucose, fasting insulin, C-peptide, HOMA-IR, triglycerides, and hsCRP, and the significant increases in HDL-cholesterol and LDL-cholesterol. The Bonferroni-adjusted significant differences from the UC cohort were also replicated by the two educational sub-cohorts for HbA_1c_, fasting glucose, insulin-derived HOMA-IR, weight, HDL-cholesterol, LDL-cholesterol, triglycerides, hsCRP, and alkaline phosphatase, with or without adjustment for baseline covariates.

### Time Course of Biomarker Change in CCI

Over the course of the intervention at baseline, 70 days [23], and 1 year, the proportion of participants in the total imputed CCI with HbA_1c_ below 48 mmol mol^−1^ (< 6.5%) increased from 19.5 ± 2.4 to 60.7 ± 3.1 to 69.8 ± 3.1%; the proportion with fasting glucose below 6.99 mmol L^−1^ (< 126 mg dL^−1^) increased from 34.9 ± 3.3 to 55.5 ± 3.3 to 58.4 ± 3.9%, and the proportion with class III obesity decreased from 45.5 ± 3.1, to 30.2 ± 3.1, to 19.6 ± 2.8%.

The time course of biomarker changes also differed by variable (see Table S3 in the electronic supplementary material). Most of the 1-year improvements in diabetes risk factors were achieved during the first 70 days of the intervention including 84% of the HbA_1c_ decrease, 90% of the fasting glucose decrease, 73% of the fasting insulin decrease, 64% of the C-peptide decrease, and 87% and 74% of the decreases in HOMA-IR as estimated from fasting insulin and C-peptide concentrations, respectively. Improvements in blood pressure also mostly occurred in the initial 70 days, as did reductions in alkaline phosphatase, serum creatinine, and eGFR. Most of the plasma triglyceride decrease occurred during the first 70 days (87%), whereas essentially all the substantial increase in HDL-cholesterol occurred between the initial 70 days of the intervention and 1 year (99%). About 60% of weight loss occurred in the first 70 days.

### Retention and Adherence in CCI

**Fig. 4:**
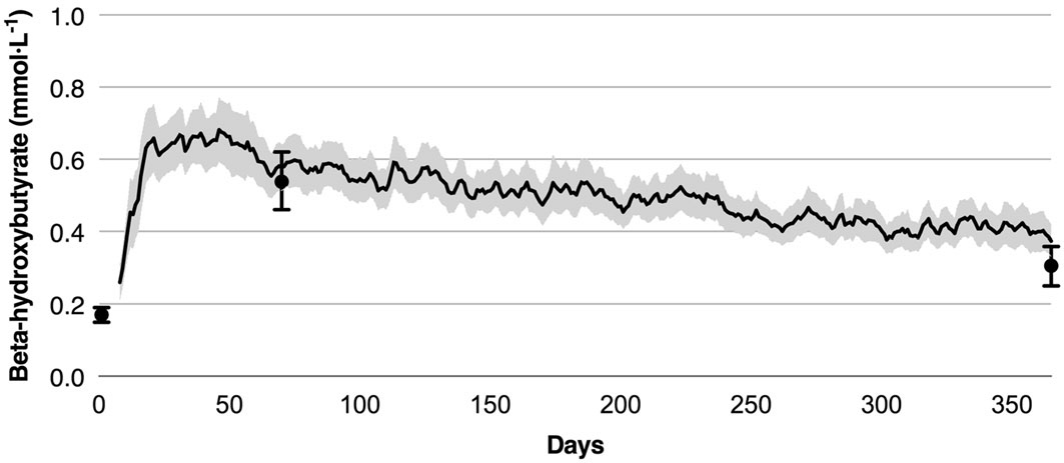
Beta-hydroxybutyrate concentrations of CCI completers. Note: For each individual in the graph, the BHB concentration on a given day was computed as the 3-day trailing mean (to reduce day-to-day variation). On dates where no BHB concentrations were recorded during the 3-day time window for a given participant, the most recent 3-day mean preceding the date was used. Line graph depicts mean (95% CI) over time for BHB measured at home and reported via the app. Dots and error bars represent the mean ± SE from laboratory measured BHB at baseline, 70 days, and 1 year

Eighty-three percent of participants remained enrolled in the CCI at 1 year. Nearly all CCI participants (96%) reported at least one BHB reading of 0.5 mmol L^−1^ or more by handheld measure, and among completers, the group mean at 70 days by laboratory measure was over threefold the baseline (0.54 ± 0.04 versus 0.17 ± 0.01 mmol L^−1^). Laboratory-measured BHB at 1 year (0.31 ± 0.03 mmol L^−1^) was nearly double the baseline value (Fig. 4). The intention-to-treat analysis yielded similar results, with an increase in average from baseline (0.17 ± 0.01 mmol L^−1^) to 70 days (0.54 ± 0.04 mmol L^−1^), followed by a decrease at 1 year (0.30 ± 0.02 mmol L^−1^), though still nearly twofold the baseline concentrations.

### Safety and Adverse Events

For CCI participants, acid–base physiology was normal; no cases of metabolic acidosis were observed. One CCI patient (0.38% of starters) had a clinically significant rise in serum creatinine, but group mean declined at 1 year. Mean blood urea nitrogen increased significantly in the CCI group, possibly indicating increased dietary protein consumption although high protein intake was not recommended. Mean uric acid in the CCI rose transiently at 70 days, but was unchanged at 1 year; no new cases of gout were diagnosed. Mean free T4 level was unchanged, and TSH was significantly lower at 1 year; two new cases of subclinical hypothyroidism were observed (0.76% of starters) in the CCI [24].

Adverse events occurred in 6/262 CCI participants including one non-ST-segment myocardial infarction, one inferior myocardial ischemia by electrocardiogram, one metastatic neuroendocrine carcinoma, one malignant cancer with multiple brain lesions and lung tumor, and death from renal hemorrhage and failure and hyperkalemia. Also, one episode of hypoglycemia occurred following a motor vehicle accident and medical records indicated the patient was not taking insulin as prescribed; no other episodes of symptomatic hypoglycemia requiring assistance were reported. None of the adverse events were attributed to the intervention.

Adverse events were reported in 6/87 UC participants, including one percutaneous coronary intervention (PCI) to left anterior descending stenosis, one PCI to right coronary artery, two carotid endarterectomies (one of which was successful), multifactorial encephalopathy, and diabetic ketoacidosis with pulmonary emboli.

## Discussion

This study evaluated the effectiveness and safety of an alternative treatment model for T2D that utilized continuous remote care to provide a high level of outpatient support combined with individualized nutrition enabling long-term maintenance of behavioral and metabolic change via nutritional ketosis. This trial prospectively observed adults with T2D undergoing treatment via this novel care model and a comparison group of adults with T2D undergoing usual care treatment. Following 1 year of CCI, participants achieved a 14 mmol mol^−1^ (1.3 ± 0.1%) decline in HbA_1c_ concurrent with 12% weight loss and reduction in medication use. Consistent conclusions were reached with intention-to-treat analysis and analysis of completers. A usual care group showed no change in diabetes status or related biomarkers over the year.

### Effectiveness

The CCI reduced HbA_1c_ by 14 mmol mol^−1^ (1.3%) at 1 year. HbA_1c_ reductions up to 7 mmol mol^−1^ (0.6%) via intensive lifestyle intervention [25] and 11 mmol mol^−1^ (1.0%) via an energy-restricted low-carbohydrate diet with partial food provision delivered via an outpatient setting [26] were previously reported. The present intervention achieved 12% weight loss at 1 year; previously studied interventions elicited 4–9% weight loss in patients with T2D [25, 26]. The regular monitoring of weight, glucose, and BHB as biometric feedback for participant, health coach, and medical provider may have provided behavior reinforcement. Further, it seems plausible that this multicomponent care model allowed for greater improvements compared to interventions that provided a subset of components. A recent primary care-led weight management intervention utilizing a 3–5 month VLCD resulted in a 10 mmol mol^−1^ (0.9%) reduction in HbA_1c_ and 10% weight loss at 1 year; 46% of participants achieved HbA_1c_ below 48 mmol mol^−1^ (< 6.5%) while taking no medications [27]. While only 25% of participants in the present investigation achieved this measure of diabetes remission, the protocol for the present investigation discontinued metformin prescription only because of contraindication, intolerance, or patient request given its efficacy for T2D prevention and recommended use in certain populations [7]. An additional 35% of participants in the present investigation were able to attain HbA_1c_ below 48 mmol mol^−1^ (< 6.5%) while taking only metformin. The longer duration of T2D and baseline insulin prescription to 30% of participants might be factors influencing the proportion of participants in which glycemic control medications could be discontinued in this investigation.

HbA_1c_ improved concurrent with medication reductions prescribed for blood glucose-lowering. For each medication class, the sum percentage of eliminations and reductions of prescriptions at 1 year exceeded that observed at 70 days [23]. Improved glycemic control via a predominantly pharmaceutical approach has demonstrated paradoxical increased cardiovascular risk [28]. Tight glycemic control can elicit symptomatic hypoglycemia [29] or weight gain [30], neither of which was observed in CCI. Thus, it is likely the treatment method by which glycemic control is achieved (e.g., pharmacological, surgery, lifestyle intervention) is important to health outcomes and risk.

Most changes in HbA_1c_, glucose, insulin, C-peptide, and HOMA-IR occurred in the first 70 days with further improvement observed at 1 year. While the mechanism for improved insulin sensitivity in ketosis is not fully understood, early improvements in HbA_1c_ and HOMA-IR indicate rapid restoration of liver and peripheral insulin sensitivity and are consistent with improvements observed within 2 weeks of ketosis when measured by euglycemic hyperinsulinemic clamp [13]. Utilization of blood BHB for self-monitoring with reinforcement by clinicians may have contributed to sustained HbA_1c_ improvement. Further, BHB acts as a signaling molecule, reducing inflammation and oxidative stress [14, 15]; therefore, mild ketonemia may benefit multiple organs and systems. With appropriate dietary formulation, benefits of nutritional ketosis are observed in mouse models of longevity and health span [31, 32]. Participant mean BHB levels are of similar magnitude to those observed with SGLT-2 inhibitor treatment (~ 0.5 mmol L^−1^) [33]. Recent trials [5, 34] demonstrate cardiovascular benefits to two SGLT-2 inhibitors; mild ketosis was postulated as a mechanism [33]. Nutritionally achieved ketosis may have long-term cardiovascular benefits without the pharmaceutical risk profile [34]. Further, presence of glucose and palmitate has been associated with beta cell apoptosis [35]. Given the reduced levels of glucose and palmitate observed during nutritional ketosis [36], it is plausible that ketosis might play a role in attenuating glucolipotoxicity-induced beta cell death.

Beyond achieving improved glycemic control concurrent with medication and weight reductions, the CCI had broad positive impact on blood pressure, liver enzymes, hsCRP, triglycerides, and HDL-C. Elevated ALT, AST, and ALP are associated with non-alcoholic fatty liver disease and non-alcoholic steatohepatitis [37]; these enzymes were significantly reduced with intervention. Rapid reduction in triglycerides and gradual rise in HDL-C observed following CCI are consistent with previously studied carbohydrate-restricted interventions and carbohydrates are well known to increase triglycerides [38]. Of the 108 CCI completers with elevated baseline triglycerides (≥ 1.69 mmol L^−1^), 54% were in normal range at 1 year. Rise in LDL-C at 1 year, occurring with significant triglyceride decrease, was expected as there is less exchange via cholesteryl ester transfer protein [39]. However, this exchange would not affect particle number and ApoB was unchanged, suggesting an overall neutral impact on LDL lipoprotein-associated cardiovascular risk. In epidemiological studies, utilization of dietary saturated fat in place of carbohydrate was associated with beneficial impact on lipid profile, cardiovascular outcomes, and mortality despite higher LDL-C [40, 41]. Transiently increased total and LDL cholesterol were also associated with mobilization of adipose cholesterol stores during major weight loss [42].

Consistent with population-level studies that observed very low rates of diabetes remission [43], the UC group had no change in HbA_1c_ and other indicators of glycemic status and insulin resistance but a net increase in diabetes medication use. Laboratory tests were generally unremarkable with biomarkers not changing significantly. The same facilities and methodologies were used for both the CCI and UC participants indicating that the changes observed in CCI participants not observed in the UC participants are unlikely to be due to methodological changes in clinical or laboratory data capture.

Despite independent recruitment of the CCI and UC groups, most of their baseline characteristics including HbA_1c_ and years since diabetes diagnosis were not significantly different. To enable a comparison between the CCI and UC groups, covariate adjustment was utilized to adjust for differences in baseline characteristics including sex, age, baseline BMI, baseline insulin use (user vs. non-user), and African-American race. With or without baseline adjustment, the change over 1 year elicited in the CC and UC groups differ in all primary outcomes—HbA_1c_, medication use, and weight—and most secondary outcomes including lipid profile, inflammation, and liver function. In general, the favorable changes observed in the CCI were not observed in the UC cohort. For example, of patients who obtained HbA_1c_ measurements at 1 year, 60% of CCI participants achieved a HbA_1c_ below 48 mmol mol^−1^ (< 6.5%) while taking no diabetes medications or metformin only, whereas only 10% of UC participants achieved this status.

One interpretation of these results is that the differences in observed outcomes over the year are due to advantages of the CCI over usual care. This suggests a need to incorporate carbohydrate restriction and comprehensive, continuous remote care as options in current guidelines for patients with diabetes as evidence accumulates [44]. However, alternative explanations are possible that may account for the large degree of difference observed. For instance, patients entering the CCI were recruited knowing that they were making a commitment to lifestyle change, while the UC participants were identified as recent referrals to local diabetes education programs and may not have had similar motivation or expectations of effort as the CCI participants. However, even when motivation is controlled for upon recruitment as an inclusion criterion for participation, additional factors may play a role in retention as evidenced by a recent study with randomization [45]. Also, the CCI and UC cohorts may also have differed in baseline characteristics that were not captured such as socioeconomic status.

Additionally, the treatment intensity of the two cohorts was not equal. The UC participants had one or more meetings with a registered dietitian and were under the medical supervision of their primary care provider or endocrinologist with periodic medical visits. In contrast, the CCI participants received a comprehensive and individualized continuous remote care intervention (and in one subgroup, the addition of on-site group classes). A more intensive intervention might have delivered somewhat better results than the investigation’s UC group. For instance, a recent in-person group-based intervention for weight loss in T2D adults reduced HbA_1c_ by 3 mmol mol^−1^ (0.3%) and weight by 4.0% after a year and medications were reduced in 26% of participants [46]. Future research might compare interventions of similar intensity with different treatment strategies to begin to understand the contribution of each component of the intervention to the overall effect.

### Adherence to CCI

Eighty-three percent of CCI participants were retained through 1 year; patient perceived benefits of favorable health outcomes, individualized continuity of care, relationship with health coach, ongoing education, biometric feedback, and peer support may have aided retention. Most participants achieved nutritional ketosis during CCI and maintained elevated BHB at 1 year, indicating sustainability and was possibly enabled by the novel use of blood BHB as daily biofeedback for adherence.

### Safety of CCI

No episodes of ketoacidosis, no hypo- or hyperglycemic events requiring assistance, and no adverse events were attributable to the CCI. With improvements or no change in liver, kidney, and thyroid function, safety of the intervention appears favorable. The absence of hypoglycemic events requiring assistance despite relatively tight glucose control may be due to the careful medical provider prescription management, especially rapid downward titration of insulin and sulfonylurea preventing hypoglycemia following dietary changes. Additionally, elevated BHB may have offered protection against hypoglycemic events, as starvation-adapted humans with elevated BHB have demonstrated full preservation of central nervous system function despite profound hypoglycemia induced by exogenous insulin [47].

### Study Strengths and Weaknesses

Prior studies have demonstrated favorable improvements in T2D status following carefully managed ketogenic diets as case series [48] or in small short-term randomized trials [45]. This study’s strengths include its prospective design, large cohort, high retention, duration, replication of findings between the CCI-onsite and CCI-web groups, and the collection of multiple time points in the intervention group allowing assessment of how biomarkers changed over time. This study also included participants prescribed insulin and with long-standing T2D, which were often exclusion criteria for prior studies. The means of recruitment, outpatient setting, and lack of food provision may enhance the real-world application of this study.

Weaknesses of this study include that it occurred at a single site and participants were mostly Caucasian. Socioeconomic and psychosocial status and genetics data were not collected. The study was not of sufficient size and duration to measure hard endpoints (e.g., mortality). Future trials could include a multi-site randomized controlled trial with greater racial and ethnic diversity, broader age range, and greater disease severity.

## Conclusions

This study demonstrated that a T2D intervention combining technology-enabled continuous remote care with individualized care plans encouraging nutritional ketosis can significantly reduce HbA_1c_, medication use, and weight within 70 days [23], and that these outcomes can be maintained or improved through 1 year. Most intervention participants with HbA_1c_ reported at 1 year achieved glycemic control in the sub-diabetes range with either no medication or the use of metformin alone. Related health parameters improved including blood pressure, lipid-lipoprotein profile, inflammation, and liver function. Ongoing research will determine the continued sustainability, effectiveness, and safety of these behavioral and metabolic changes.

## Electronic supplementary material

Below is the link to the electronic supplementary material.

Supplementary material 1 (PDF 539 kb)

## Abbreviations

ALP: Alkaline phosphatase
ALT: Alanine aminotransferase
ApoB: Apolipoprotein B
AST: Aspartate aminotransferase
BHB: Beta-hydroxybutyrate
BUN: Blood urea nitrogen
CBC: Complete blood count
CCI: Continuous care intervention
CCI-onsite: Subset of participants who selected on-site education
CCI-web: Subset of participants who selected web-based education
CMP: Complete metabolic panel
DPP-4: Dipeptidyl peptidase-4 inhibitor
eGFR: Estimated glomerular filtration rate
FT4: Free T4
GLP-1: Glucagon-like peptide 1 receptor agonists
HOMA-IR: Homeostatic model assessment of insulin resistance
hsCRP: High-sensitivity C-reactive protein
PCP: Primary care provider
SGLT-2: Sodium glucose co-transporter 2 inhibitors
T2D: Type 2 diabetes
TSH: Thyroid stimulating hormone
UC: Usual care
VLCD: Very low energy diet
