# Author Correction: Effectiveness and Safety of a Novel Care Model for the Management of Type 2 Diabetes at 1 Year: An Open-Label, Non-Randomized, Controlled Study

**DOI:** 10.1007/s13300-018-0386-4

**Published:** 2018-03-05

**Authors:** Sarah J. Hallberg, Amy L. McKenzie, Paul T. Williams, Nasir H. Bhanpuri, Anne L. Peters, Wayne W. Campbell, Tamara L. Hazbun, Brittanie M. Volk, James P. McCarter, Stephen D. Phinney, Jeff S. Volek

**Affiliations:** 1 Medically Supervised Weight Loss, Indiana University Health Arnett, Lafayette, IN USA; 2 Virta Health, San Francisco, CA USA; 3 Independent Consultant, Lafayette, CA USA; 4 Keck School of Medicine, University of Southern California, Los Angeles, CA USA; 5 Department of Nutrition Science, Purdue University, West Lafayette, IN USA; 6 Department of Genetics, Washington University School of Medicine, St. Louis, MO USA; 7 Department of Human Sciences, The Ohio State University, Columbus, OH USA

## Author Correction to: Diabetes Ther 10.1007/s13300-018-0373-9

The original version of this article unfortunately contained a mistake.

The presentation of Table 1 and ESM 1 have been published incorrectly. Corrected units have been provided for LDL-C values. The corrected Table 1 in text is given below and ESM Table 1 is available online.

**Table 1:** Baseline characteristics of the recruited sample, completers, and participants with missing data by treatment arm

	All		Completers with data		Dropout or missing data		Completers–dropouts
	*N*	Mean (SD) or ± SE	*N*	Mean (SD) or ± SE	*N*	Mean (SD) or ± SE	Mean ± SE
Age (years)							
CCI-all education^a^	262	53.75 (8.35)	218	54.09 (8.35)	44	52.09 (8.25)	2.0 ± 1.37
Usual care^a^	87	52.33 (9.52)	78	51.71 (9.62)	9	57.78 (6.85)	− 6.07 ± 2.53*
CCI-all vs. usual care^b^		1.42 ± 1.14		2.38 ± 1.23*		− 5.69 ± 2.6*	
Female (%)							
CCI-all education^a^	262	66.79 ± 2.91	218	65.14 ± 3.23	44	75.0 ± 6.53	− 9.86 ± 7.28
Usual care^a^	87	58.62 ± 5.28	78	60.26 ± 5.54	9	44.44 ± 16.56	15.81 ± 17.47
CCI-all vs. usual care^b^		8.17 ± 6.03		4.88 ± 6.41		30.56 ± 17.8	
African American (%)							
CCI-all education^a^	262	6.87 ± 1.56	218	5.96 ± 1.6	44	11.36 ± 4.78	− 5.4 ± 5.05
Usual care^a^	87	0.0 ± 0.0	78	0.0 ± 0.0	9	0.0 ± 0.0	0.0 ± 0.0
CCI-all vs. usual care^b^		6.87 ± 1.56§		5.96 ± 1.6‡		11.36 ± 4.78*	
Years with type 2 diabetes							
CCI-all education^a^	261	8.44 (7.22)	217	8.4 (7.28)	44	8.61 (6.97)	− 0.21 ± 1.16
Usual care^a^	71	7.85 (7.32)	71	7.85 (7.32)		Not collected	
CCI-all vs. usual care^b^		0.59 (0.9)		0.56 ± 1.0			
Beta-hydroxybutyrate (mmol L^−1^)							
CCI-all education^a^	248	0.17 (0.15)	186	0.17 (0.15)	62	0.19 (0.16)	− 0.02 ± 0.02
Usual care^a^	79	0.15 (0.13)	59	0.14 (0.12)	20	0.17 (0.15)	− 0.03 ± 0.03
CCI-all vs. usual care^b^		0.02 ± 0.02		0.02 ± 0.02		0.02 ± 0.04	
Hemoglobin A_1c_ (mmol mol^−1^)							
CCI-all education^a^	262	59.55 (16.4)	204	58.35 (15.3)	58	63.49 (19.57)	− 28.66 ± 2.73
Usual care^a^	87	59.99 (19.24)	72	61.08 (19.89)	15	54.52 (14.87)	− 16.97 ± 4.48
CCI-all vs. usual care^b^		− 0.44 ± 2.3		− 2.73 ± 2.62		8.96 ± 4.59*	
Hemoglobin A_1c_ (%)							
CCI-all education^a^	262	7.60 (1.50)	204	7.49 (1.4)	58	7.96 (1.79)	− 0.47 ± 0.25
Usual care^a^	87	7.64 (1.76)	72	7.74 (1.82)	15	7.14 (1.36)	0.60 ± 0.41
CCI-all vs. usual care^b^		− 0.04 ± 0.21		− 0.25 ± 0.24		0.82 ± 0.42*	
Fasting glucose (mmol L^−1^)							
CCI-all education^a^	258	8.92 (3.41)	202	8.8 (3.28)	56	9.36 (3.83)	− 0.55 ± 0.56
Usual care^a^	86	8.67 (4.03)	71	8.71 (3.96)	15	8.5 (4.5)	0.21 ± 1.25
CCI-all vs. usual care^b^		0.25 ± 0.48		0.1 ± 0.52		0.86 ± 1.27	
Insulin all (pmol L^−1^)							
CCI-all education^a^	248	198.35 (165.85)	186	197.65 (167.17)	62	200.5 (163.21)	− 2.85 ± 24.1
Usual care^a^	79	202.17 (172.58)	59	206.68 (187.93)	20	188.77 (119.18)	17.99 ± 36.18
CCI-all vs. usual care^b^		− 3.82 ± 22.09		− 9.1 ± 27.36		11.74 ± 33.75	
C-peptide (nmol L^−1^)							
CCI-all education^a^	247	1.45 (0.71)	185	1.47 (0.72)	62	1.39 (0.69)	0.07 ± 0.1
Usual care^a^	79	1.38 (0.82)	59	1.35 (0.82)	20	1.49 (0.84)	− 0.14 ± 0.22
CCI-all vs. usual care^b^		0.07 ± 0.1		0.12 ± 0.12		− 0.09 ± 0.21	
HOMA-IR (insulin derived), all							
CCI-all education^a^	244	11.8 (13.14)	179	11.19 (12.75)	65	13.48 (14.12)	− 2.3 ± 1.99
Usual care^a^	78	10.64 (9.12)	56	11.31 (10.05)	22	8.94 (6.03)	2.36 ± 1.86
CCI-all vs. usual care^b^		1.16 ± 1.33		− 0.12 ± 1.65		4.54 ± 2.17	
HOMA-IR (C-peptide derived)							
CCI-all education^a^	244	11.52 (7.15)	170	11.44 (6.26)	69	11.72 (9.04)	− 0.28 ± 1.19
Usual care^a^	72	11.16 (7.26)	47	10.56 (7.70)	25	12.29 (6.33)	− 1.73 ± 1.69
CCI-all vs. usual care^b^		0.36 ± 0.97		0.88 ± 1.22		− 0.56 ± 1.67	
Weight-clinic (kg)							
CCI-all education^a^	257	116.51 (25.94)	184	115.42 (24.62)	73	119.25 (29.01)	− 3.83 ± 3.85
Usual care^a^	83	105.63 (22.15)	69	106.79 (22.18)	14	99.94 (21.86)	6.84 ± 6.42
CCI-all vs. usual care^b^		10.87 ± 2.92§		8.63 ± 3.23†		19.3 ± 6.76†	
BMI (kg m^−2^)							
CCI-all education^a^	257	40.43 (8.81)	184	39.87 (7.88)	73	41.82 (10.75)	− 1.94 ± 1.39
Usual care^a^	83	36.72 (7.26)	69	37.14 (7.62)	14	34.66 (4.8)	2.48 ± 1.58
CCI-all vs. usual care^b^		3.7 ± 0.97‡		2.73 ± 1.09†		7.15 ± 1.8§	
Systolic blood pressure (mmHg)							
CCI-all education^a^	260	131.94 (14.09)	187	132.51 (14.54)	73	130.47 (12.84)	2.05 ± 1.84
Usual care^a^	79	129.8 (13.61)	67	128.72 (12.65)	12	135.83 (17.49)	− 7.12 ± 5.28
CCI-all vs. usual care^b^		2.14 ± 1.76		3.8 ± 1.88*		− 5.37 ± 5.27	
Diastolic blood pressure (mmHg)							
CCI-all education^a^	260	82.09 (8.25)	187	81.59 (8.05)	73	83.37 (8.67)	− 1.78 ± 1.17
Usual care^a^	79	82.0 (8.93)	67	81.1 (8.07)	12	87.0 (11.95)	− 5.9 ± 3.59
CCI-all vs. usual care^b^		0.09 ± 1.13		0.49 ± 1.15		− 3.63 ± 3.6	
Total cholesterol (mmol L^−1^)							
CCI-all education^a^	247	4.76 (1.07)	186	4.68 (1.03)	61	4.99 (1.15)	− 0.31 ± 0.17
Usual care^a^	79	4.76 (1.19)	59	4.72 (1.26)	20	4.88 (0.93)	− 0.16 ± 0.27
CCI-all vs. usual care^b^		− 0.0 ± 0.15		− 0.04 ± 0.18		0.11 ± 0.26	
LDL-cholesterol (mmol L^−1^)							
CCI-all education^a^	232	2.66 (0.85)	172	2.59 (0.84)	60	2.84 (0.86)	− 0.24 ± 0.13
Usual care^a^	70	2.63 (0.94)	48	2.60 (0.98)	22	2.69 (0.85)	− 0.09 ± 0.23
CCI-all vs. usual care^b^		0.03 ± 0.13		− 0.01 ± 0.16		0.14 ± 0.21	
Apo B (g L^−1^)							
CCI-all education^a^	248	1.05 (0.29)	186	1.03 (0.28)	62	1.1 (0.31)	− 0.06 ± 0.04
Usual care^a^	79	1.07 (0.28)	59	1.06 (0.3)	20	1.11 (0.24)	− 0.05 ± 0.07
CCI-all vs. usual care^b^		− 0.02 ± 0.04		− 0.02 ± 0.04		− 0.01 ± 0.07	
HDL-C (mmol L^−1^)							
CCI-all education^a^	247	1.09 (0.35)	186	1.1 (0.36)	61	1.08 (0.32)	0.02 ± 0.05
Usual care^a^	79	0.97 (0.29)	59	0.96 (0.29)	20	1.02 (0.29)	− 0.06 ± 0.08
CCI-all vs. usual care^b^		0.12 ± 0.04†		0.14 ± 0.05†		0.06 ± 0.08	
Triglycerides (mmol L^−1^)							
CCI-all education^a^	247	2.23 (1.62)	186	2.27 (1.73)	61	2.11 (1.25)	0.15 ± 0.2
Usual care^a^	79	3.2 (4.53)	59	3.36 (5.17)	20	2.72 (1.56)	0.64 ± 0.76
CCI-all vs. usual care^b^		− 0.97 ± 0.52*		− 1.09 ± 0.68		− 0.61 ± 0.38*	
Total/HDL-cholesterol							
CCI-all education^a^	247	4.72 (1.7)	186	4.65 (1.72)	61	4.93 (1.65)	− 0.28 ± 0.25
Usual care^a^	79	5.37 (2.42)	59	5.44 (2.63)	20	5.17 (1.72)	0.27 ± 0.52
CCI-all vs. usual care^b^		− 0.65 ± 0.29*		− 0.79 ± 0.36*		− 0.24 ± 0.44	
hsC-reactive protein (nmol L^−1^)							
CCI-all education^a^	249	81.33 (138.0)	193	85.62 (153.05)	56	66.76 (62.1)	18.86 ± 13.81
Usual care^a^	85	84.67 (82.1)	70	86.95 (86.95)	15	73.81 (73.81)	13.14 ± 19.14
CCI-all vs. usual care^b^		− 3.24 ± 12.48		− 1.33 ± 15.05		− 7.05 ± 18.19	
ALT (µkat L^−1^)							
CCI-all education^a^	257	0.51 (0.38)	201	0.52 (0.41)	56	0.47 (0.27)	0.05 ± 0.05
Usual care^a^	86	0.46 (0.33)	71	0.45 (0.34)	15	0.51 (0.29)	− 0.05 ± 0.09
CCI-all vs. usual care^b^		0.05 ± 0.04		0.07 ± 0.05		− 0.04 ± 0.08	
AST (µkat L^−1^)							
CCI-all education^a^	257	0.4 (0.25)	201	0.41 (0.28)	56	0.36 (0.15)	0.04 ± 0.03
Usual care^a^	86	0.4 (0.32)	71	0.39 (0.35)	15	0.42 (0.16)	− 0.03 ± 0.06
CCI-all vs. usual care^b^		− 0.0 ± 0.04		0.01 ± 0.05		− 0.06 ± 0.05	
Alkaline phosphatase (µkat L^−1^)							
CCI-all education^a^	256	1.24 (0.37)	200	1.24 (0.37)	56	1.23 (0.36)	0.01 ± 0.05
Usual care^a^	86	1.29 (0.44)	71	1.31 (0.45)	15	1.22 (0.38)	0.09 ± 0.11
CCI-all vs. usual care^b^		− 0.05 ± 0.05		− 0.07 ± 0.06		0.01 ± 0.11	
Serum creatinine (µmol L^−1^)							
CCI-all education^a^	258	77.79 (21.22)	202	77.79 (20.33)	56	81.33 (24.75)	− 3.54 ± 3.54
Usual care^a^	86	80.44 (22.1)	71	78.68 (20.33)	15	86.63 (25.64)	− 7.07 ± 7.07
CCI-all vs. usual care^b^		− 1.77 ± 2.65		− 1.77 ± 2.65		− 5.3 ± 7.07	
BUN (mmol L^−1^)							
CCI-all education^a^	258	6.03 (2.34)	202	6.06 (2.15)	56	5.9 (2.96)	0.16 ± 0.42
Usual care^a^	86	5.73 (2.23)	71	5.59 (1.86)	15	6.38 (3.52)	− 0.79 ± 0.94
CCI-all vs. usual care^b^		0.3 ± 0.28		0.47 ± 0.27		− 0.47 ± 0.99	
eGFR (mL s^−1^ m^−2^)							
CCI-all education^a^	258	1.34 (0.23)	202	1.35 (0.22)	56	1.33 (0.25)	0.02 ± 0.04
Usual care^a^	86	1.32 (0.23)	71	1.34 (0.22)	15	1.26 (0.28)	0.08 ± 0.08
CCI-all vs. usual care^b^		0.02 ± 0.03		0.02 ± 0.03		0.03 ± 0.08	
Anion gap (mmol L^−1^)							
CCI-all education^a^	257	6.83 (1.67)	201	6.79 (1.7)	56	6.98 (1.53)	− 0.19 ± 0.24
Usual care^a^	86	6.93 (1.82)	71	6.92 (1.82)	15	7.0 (1.89)	− 0.08 ± 0.53
CCI-all vs. usual care^b^		− 0.1 ± 0.22		− 0.12 ± 0.25		− 0.02 ± 0.53	
Uric acid (µmol L^−1^)							
CCI-all education^a^	261	347.99 (86.85)	202	348.58 (86.25)	59	346.2 (89.82)	2.38 ± 13.09
Usual care^a^	85	333.12 (87.44)	71	330.74 (85.66)	14	345.01 (98.75)	− 14.28 ± 28.55
CCI-all vs. usual care^b^		14.87 ± 10.71		17.25 ± 11.9		1.19 ± 29.15	
TSH (mIU L^−1^)							
CCI-all education^a^	259	2.32 (1.74)	200	2.31 (1.79)	59	2.38 (1.55)	− 0.07 ± 0.24
Usual care^a^	85	1.97 (1.16)	70	2.09 (1.16)	15	1.38 (1.03)	0.71 ± 0.3*
CCI-all vs. usual care^b^		0.36 ± 0.17*		0.21 ± 0.19		1.0 ± 0.33†	
Free T4 (pmol L^−1^)							
CCI-all education^a^	260	11.84 (2.19)	202	11.84 (2.32)	58	11.58 (2.19)	0.26 ± 0.39
Usual care^a^	86	11.33 (3.73)	71	11.33 (3.86)	15	10.94 (2.32)	0.39 ± 0.77
CCI-all vs. usual care^b^		0.51 ± 0.39		0.51 ± 0.51		0.64 ± 0.64	
Any diabetes medication, excluding metformin (%)							
CCI-all education^a^	262	56.87 ± 3.06	218	55.50 ± 3.37	44	63.64 ± 7.25	− 8.13 ± 8.00
Usual care^a^	87	66.67 ± 5.05	73	68.49 ± 5.44	14	57.14 ± 13.23	11.35 ± 14.32
CCI-all vs. usual care^b^		− 9.80 ± 5.91		− 12.99± 6.39*		6.49 ± 15.08	
Sulfonylurea (%)							
CCI-all education^a^	262	23.66 ± 2.63	218	24.31 ± 2.91	44	20.45 ± 6.08	3.86 ± 6.74
Usual care^a^	87	24.14 ± 4.59	73	23.29 ± 4.95	14	28.57 ± 12.07	− 5.28 ± 13.05
CCI-all vs. usual care^b^		− 0.48 ± 5.29		1.02 ± 5.74		− 8.12± 13.52	
Insulin (%)							
CCI-all education^a^	262	29.77 ± 2.82	218	28.44 ± 3.06	44	36.36 ± 7.25	− 7.92 ± 7.87
Usual care^a^	87	45.98 ± 5.34	78	50.0 ± 5.66	9	11.11 ± 10.48	38.89 (1.91)‡
CCI-all vs. usual care^b^		− 16.21 ± 6.04†		− 21.56 ± 6.43‡		25.25 ± 12.74*	
Thiazolidinedione (%)							
CCI-all education^a^	262	1.53 ± 0.76	218	1.83 ± 0.91	44	0.0 ± 0.0	1.83 ± 0.91*
Usual care^a^	87	1.15 ± 1.14	73	1.37 ± 1.36	14	0.0 ± 0.0	1.37 ± 1.36
CCI-all vs. usual care^b^		0.38 ± 1.37		0.46 ± 1.64		0.0 ± 0.0	
SGLT-2 (%)							
CCI-all education^a^	262	10.31 ± 1.88	218	10.55 ± 2.08	44	9.09 ± 4.33	1.46 ± 4.81
Usual care^a^	87	13.79 ± 3.7	73	15.07 ± 4.19	14	7.14 ± 6.88	7.93 ± 8.06
CCI-all vs. usual care^b^		− 3.48 ± 4.15		− 4.52 ± 4.68		1.95 ± 8.13*	
DPP-4 (%)							
CCI-all education^a^	262	9.92 ± 1.85	218	10.09 ± 2.04	44	9.09 ± 4.33	1.0 ± 4.79
Usual care^a^	87	8.05 ± 2.92	73	8.22 ± 3.21	14	7.14 ± 6.88	1.08 ± 7.60
CCI-all vs. usual care^b^		1.87 ± 3.45		1.87 ± 3.81		1.95 ± 8.13	
GLP-1 (%)							
CCI-all education^a^	262	13.36 ± 2.1	218	12.84 ± 2.27	44	15.91 ± 5.51	− 3.07 ± 5.96
Usual care^a^	87	14.94 ± 3.82	73	16.44 ± 4.34	14	7.14 ± 6.88	9.30 ± 8.14
CCI-all vs. usual care^b^		− 1.58 ± 4.36		− 3.59 ± 4.89		8.77 ± 8.82	
Metformin (%)							
CCI-all education^a^	262	71.37 ± 2.79	218	71.56 ± 3.06	44	70.45 ± 6.88	1.11 ± 7.53
Usual care^a^	87	60.92 ± 5.23	73	61.64 ± 5.69	14	57.14 ± 13.23	4.50 ± 14.40
CCI-all vs. usual care^b^		10.45 ± 5.93		9.92 ± 6.46		13.31 ± 14.91	

See Table S1 (electronic supplemental materials) for CCI-web, CCI-onsite, and additional comparisons

^a^Mean and standard deviations for continuous variables, percents and standard errors for categorical variables

^b^Difference between means or percentages ± 1 standard error of the difference. Significant baseline difference between means or percentages at 0.05 > *P* ≥ 0.01 (*); 0.01 > *P* ≥ 0.001 (†); 0.001 > *P* ≥ 0.0001 (‡); and *P* < 0.0001 (§)

## Electronic supplementary material

Below is the link to the electronic supplementary material.

Supplementary material 1 (PDF 206 kb)

