# Supplementary material for: Effectiveness and Safety of a Novel Care Model for the Management of Type 2 Diabetes at 1 Year: An Open-Label, Non-Randomized, Controlled Study
Source: Diabetes Ther. 2018 Feb 7;9(2):583–612. doi: 10.1007/s13300-018-0373-9 (PMC6104272; doi:10.1007/s13300-018-0373-9)
Supplement: Supplementary file 1 — Supplementary material 1 (PDF 539 kb) [file 13300_2018_373_MOESM1_ESM.pdf]

Supplementary Materials

Effectiveness and Safety of a Novel Care Model for the Management of Type 2 Diabetes at One Year: An Open-Label, Non-Randomized, Controlled Study

Supplementary Materials A.

<u>Methods - Full Inclusion and Exclusion Criteria</u>

Participants were 21 to 65 years old and were excluded if they had serious renal, hepatic, or cardiovascular dysfunction, infectious disease, uncontrolled psychiatric disorder, history of ketoacidosis, intolerance to dietary fat, cancer with active treatment in the last five years, pregnancy, or planned pregnancy.

Inclusion Criteria:

- Diabetes: Diagnosis of type-2 diabetes without end-organ failure
- BMI: > 25
- Ethnicity: all ethnic groups
- Age: 21 to 65 years

Exclusion Criteria:

- Type 1 diabetes
- History of keto-acidosis
- History of myocardial infarction, stroke, angina, or coronary insufficiency within the last 6 months
- Diabetic retinopathy requiring treatment
- Creatinine > 2.0 $mg \cdot dL^{-1}$ or > 152.5 $\mu mol \cdot L^{-1}$
- Urinary albumin > 1 $g \cdot dL^{-1}$ or > 10 $g \cdot L^{-1}$
- Impaired hepatic function (Bilirubin >2 $mg \cdot dL^{-1}$ or >34.2 $\mu mol \cdot L^{-1}$, Albumin < 3.5 $g \cdot dL^{-1}$ or <35 $g \cdot L^{-1}$)
- Cholelithiasis or biliary dysfunction
- Cancer requiring treatment in the past 5 years, with the exception of non-melanoma skin cancer
- Chronic infectious disease requiring ongoing treatment
- Other chronic diseases or condition likely to limit lifespan to <6 years
- Non-English speaking
- Unable or unwilling to participate in group sessions (e.g., plans to relocate within the next year) or conform to a carbohydrate restricted diet lifestyle (e.g., food intolerances, religious or personal restrictions)
- Weight loss of >10% in past 6 months
- Currently pregnant or nursing, or planning to become pregnant during the study
- Major psychiatric disorder (e.g., schizophrenia, bipolar disorder) currently uncontrolled
- Excessive alcohol intake (acute or chronic) defined as average consumption of 3 or more alcohol-containing beverages daily or consumption of more than 14 alcoholic beverages per week

Supplementary Materials B.

Methods - Medication Management

All medications changes were tracked in a database visible to the medical provider and health coach. Upon commencing dietary changes, insulin and sulfonylurea doses were reduced or eliminated to safely adjust for anticipated decreases in glucose concentrations based on the study physician's clinical judgment of the participant's most recent HbA_{1c}, participant-reported last two weeks of blood glucose measurements, and other concurrent anti-diabetic medications. Similarly, prescription of SGLT-2 inhibitors were discontinued at onset of dietary changes.[1] In the following days and weeks, participants reported BHB and glucoses one to three times per day via the app, based on medical provider instructions. Health coaches and medical providers monitored BHB and glucose, and the medical provider prescribed medications changes as necessary based on glucose and BHB trends and concurrent medication use with a goal of maintaining any reported glucose (fasting, pre-meal or post-meal) between 120 and 180 $mg \cdot dL^{-1}$ (6.66 and 9.99 $mmol \cdot L^{-1}$) until the medication could be eliminated. During medication management, the primary focus was on preventing episodes of symptomatic hypoglycemia in patients. Any reported blood glucose <100 $mg \cdot dL^{-1}$ (<5.55 $mmol \cdot L^{-1}$) prompted insulin or sulfonylurea reduction of 10-50%. Medication eliminations typically occurred by first discontinuing sulfonylureas, followed by short-acting, and then long-acting insulin. Every patient prescribed short-acting insulin was instructed not to take any insulin if blood glucose was <120 $mg \cdot dL^{-1}$ (<6.66 $mmol \cdot L^{-1}$); for blood glucose ≥120 $mg \cdot dL^{-1}$ (≥6.66 $mmol \cdot L^{-1}$), the newly prescribed dose was reduced in 20 - 50% increments in anticipation of reduction in dietary carbohydrates until the bolus or sliding scale was no longer needed. Thiazolidinediones, DPP-4 inhibitors, and GLP-1 were discontinued after elimination of insulin and sulfonylureas and reported blood glucoses were consistently under 100 $mg \cdot dL^{-1}$ (5.55 $mmol \cdot L^{-1}$). Metformin, given its effectiveness, low cost, and tolerability[2,3], was generally not eliminated unless the participants reported side effects or requested discontinuation.

At any time during the study, reported blood glucoses consistently above 180 $mg \cdot dL^{-1}$ (9.99 $mmol \cdot L^{-1}$), generally, >3 times within a week and sustained for >1 week, prompted a medication increase or addition. If the participant was previously prescribed insulin, long-acting insulin was the first medication re-started. If the participant was previously prescribed a DPP-4 inhibitor, GLP-1, or a thiazolidinedione without insulin or sulfonylurea use, these were re-started. Two participants self-resumed taking their SGLT-2 inhibitors without physician direction. In these participants, BHB was monitored at least once daily, and no values of concern were reported. In all participants, blood glucose levels were monitored for trends up and down, which prompted the appropriate medication adjustments.

Fifty percent of CCI participants entered the trial with statin prescriptions. Among completers, prescriptions were discontinued at patient request (n=18). In some cases, prescriptions were added (n=10) or increased (n=3) when deemed beneficial. Medication adjustments occurred both via remote care and at on-site clinic visits.

Supplementary Materials C.

Methods - Education

Education provided participants an understanding of the core concepts and implementation instructions to enable initial achievement of nutritional ketosis and behavior change. Content included the pathophysiology of diabetes, practical management of carbohydrate restriction while consuming protein in moderation and increasing fat intake with a goal of achieving nutritional ketosis, how to assess and utilize BHB as a biofeedback mechanism and implementing of behavior change techniques. Additional resources were provided to participants to supplement their education, such as guides, blog posts, and recipes.

Examples of Educational Modules

- Introduction
- Prepare for Adaptation
- Adaptation
- Understanding ketones and self-testing
- Navigating the social scene
- Beverages and sweeteners
- Dietary supplements
- Cholesterol and lipids
- Fiber
- Goal setting
- Body image
- Personalizing physical activity
- Maintenance
- Advanced lipid metabolism

Supplementary Materials D.

Methods – Laboratory Analysis

Blood analytes determined via standard procedures at a CLIA accredited laboratory at the time of draw included the following tests and panels: HbA_{1c}, complete blood count (CBC), complete metabolic panel (CMP), high-sensitive C-reactive protein (hsCRP), uric acid, thyroid stimulating hormone (TSH), free T4 (FT4), and lipids. HbA_{1c} was determined from EDTA-treated whole blood using high performance liquid chromatography on Bio-Rad Variant II Turbo 2.0. Complete blood count with differential was determined from EDTA-treated whole blood with flow cytometry on a Beckman Coulter DxH80. Lithium-heparin treated plasma was utilized to determine the CMP, hsCRP, and uric acid with a Beckman Coulter AU680 and to determine TSH and FT4 via chemiluminescent detection on the Beckman Coulter DXI600. Estimated glomerular filtration rate (eGFR) was calculated from serum creatinine, gender, race, and age[4]. 5.2% of all participants were African American.

Aliquots of serum were stored at -80°C and thawed once for later analysis of blood lipids, insulin, C-peptide, and BHB by a CLIA accredited laboratory. FDA approved methods were utilized for insulin, C-peptide (cobas e601, Roche Diagnostics; Indianapolis, IN, USA) and ApoB, total cholesterol, triglycerides, direct HDL (cobas c501, Roche Diagnostics; Indianapolis, IN, USA). LDL was calculated using the Friedewald equation. Beta-hydroxybutyrate was performed with research use only assays, using a coupled enzymatic assay (Stanbio Laboratory, Boerne, TX) on an open channel of the cobas c501. HOMA-IR derived from fasting serum insulin

concentrations was calculated as (fasting insulin x fasting glucose)/22.5 in participants not using insulin medication.[5] Homeostatic model assessment for insulin resistance (HOMA-IR) derived from C-peptide was calculated on all participants without regard to their insulin use from the updated HOMA2 (computer) model.[6]

Supplementary Materials References

1. Handelsman Y, Henry RR, Bloomgarden ZT, Dagogo-Jack S, DeFronzo RA, Einhorn D, et al. American Association of Clinical Endocrinologists and American College of Endocrinology Position Statement on the Association of SGLT-2 Inhibitors and Diabetic Ketoacidosis. Endocrine Practice. 2016 Jun;22(6):753–62.

2. American Diabetes Association. Standards of Medical Care in Diabetes—2018. Diabetes Care. 2018 Jan 1;41(Supplement 1):S1–S172.

3. Handelsman Y, Bloomgarden ZT, Grunberger G, Umpierrez G, Zimmerman RS, Bailey TS, et al. American Association of Clinical Endocrinologists and American College of Endocrinology – Clinical Practice Guidelines for Developing a Diabetes Mellitus Comprehensive Care Plan – 2015. Endocrine Practice. 2015 Apr;21(Supplement 1):1–87.

4. Levey AS, Stevens LA, Schmid CH, Zhang YL, Castro AF III, Feldman HI, et al. A New Equation to Estimate Glomerular Filtration Rate. Ann Intern Med. 2009 May 5;150(9):604–16.

5. Matthews DR, Hosker JP, Rudenski AS, Naylor BA, Treacher DF, Turner RC. Homeostasis model assessment: insulin resistance and ?-cell function from fasting plasma glucose and insulin concentrations in man. Diabetologia. 1985 Jul;28(7):412–9.

6. Levy JC, Matthews DR, Hermans MP. Correct homeostasis model assessment (HOMA) evaluation uses the computer program. Diabetes Care. 1998 Dec;21(12):2191–2.

Table S1. Baseline characteristics of the recruited sample, completers, and participants with missing data by treatment arm.

	All		Completers with data		Dropout or missing data		Completers-Dropouts
	N	Mean (SD) or ±SE	N	Mean (SD) or ±SE	N	Mean (SD) or ±SE	Mean ± SE
**Age (years)**							
All	349	53.4 (8.67)	296	53.46 (8.75)	53	53.06 (8.25)	0.4 ± 1.24
CCI-all education[a]	262	53.75 (8.35)	218	54.09 (8.35)	44	52.09 (8.25)	2.0 ± 1.37
CCI-web[a]	126	53.39 (8.19)	104	53.49 (8.23)	22	51.27 (8.44)	0.58 ± 1.92
CCI-onsite[a]	136	54.09 (8.52)	114	54.63 (8.47)	9	57.78 (6.85)	3.36 ± 1.97
Usual care[a]	87	52.33 (9.52)	78	51.71 (9.62)	9	57.78 (6.85)	-6.07 ± 2.53*
CCI-web vs. CCI-onsite[b]		-0.7 ± 1.03		-1.14 ± 1.13		1.64 ± 2.5	
CCI-web vs. usual care[b]		1.06 ± 1.25		1.79 ± 1.36		-4.87 ± 2.87	
CCI-onsite vs. usual care[b]		1.75 ± 1.26		2.93 ± 1.35		-6.51 ± 2.91	
CCI-all vs. usual care[b]		1.42 ± 1.14		2.38 ± 1.23*		-5.69 ± 2.6*	
**Female (%)**							
All	349	64.76 ± 2.55	296	63.85 ± 2.79	53	69.81 ± 6.31	-5.96 ± 6.9
CCI-all education[a]	262	66.79 ± 2.91	218	65.14 ± 3.23	44	75.0 ± 6.53	-9.86 ± 7.28
CCI-web[a]	126	61.9 ± 4.33	104	57.69 ± 4.84	22	81.82 ± 8.22	-24.13 ± 9.54*
CCI-onsite[a]	136	71.32 ± 3.88	114	71.93 ± 4.21	22	68.18 ± 9.93	3.75 ± 10.79
Usual care[a]	87	58.62 ± 5.28	78	60.26 ± 5.54	9	44.44 ± 16.56	15.81 ± 17.47
CCI-web vs. CCI-onsite[b]		-9.42 ± 5.81		-14.24 ± 6.42*		13.64 ± 12.89	
CCI-web vs. usual care[b]		3.28 ± 6.83		-2.56 ± 7.36		37.37 ± 18.49*	
CCI-onsite vs. usual care[b]		12.7 ± 6.55*		11.67 ± 6.96		23.74 ± 19.31	
CCI-all vs. usual care[b]		8.17 ± 6.03		4.88 ± 6.41		30.56 ± 17.8	
**African American (%)**							
All	349	5.16 ± 1.18	296	4.39 ± 1.19	53	9.43 ± 4.02	-5.04 ± 4.19
CCI-all education[a]	262	6.87 ± 1.56	218	5.96 ± 1.6	44	11.36 ± 4.78	-5.4 ± 5.05
CCI-web[a]	126	5.56 ± 2.04	104	5.77 ± 2.29	22	4.55 ± 4.44	1.22 ± 4.99
CCI-onsite[a]	136	8.09 ± 2.34	114	6.14 ± 2.25	22	18.18 ± 8.22	-12.04 ± 8.52
Usual care[a]	87	0.0 ± 0.0	78	0.0 ± 0.0	9	0.0 ± 0.0	0.0 ± 0.0
CCI-web vs. CCI-onsite[b]		-2.53 ± 3.1		-0.37 ± 3.21		-13.64 ± 9.35	
CCI-web vs. usual care[b]		5.56 ± 2.04†		5.77 ± 2.29*		4.55 ± 4.44	
CCI-onsite vs. usual care[b]		8.09 ± 2.34‡		6.14 ± 2.25†		18.18 ± 8.22*	
CCI-all vs. usual care[b]		6.87 ± 1.56§		5.96 ± 1.6‡		11.36 ± 4.78*	

**Years with type 2 diabetes**							
All	332	8.31 (7.23)	288	8.26 (7.28)	44	8.61 (6.97)	-0.35 ± 1.13
CCI-all education[a]	261	8.44 (7.22)	217	8.4 (7.28)	44	8.61 (6.97)	-0.21 ± 1.16
CCI-web[a]	126	7.88 (6.79)	104	7.95 (7.03)	22	7.55 (5.65)	0.41 ± 1.39
CCI-onsite[a]	135	8.96 (7.58)	113	8.81 (7.51)	22	9.68 (8.07)	-0.87 ± 1.86
Usual care[a]	71	7.85 (7.32)	71	7.85 (7.32)		Not collected	
CCI-web vs. CCI-onsite[b]		-1.07 ± 0.89		-0.86 ± 0.99			-2.14 ± 2.1
CCI-web vs. usual care[b]		0.04 ± 1.06		0.11 ± 1.11			
CCI-onsite vs. usual care[b]		1.11 ± 1.09		0.97 ± 1.12			
CCI-all vs. usual care[b]		0.59 (0.9)		0.56 ± 1.0			
**Beta-hydroxybutyrate (mmol·L^{-1})**							
All	327	0.17 (0.15)	245	0.16 (0.14)	82	0.19 (0.16)	-0.02 ± 0.02
CCI-all education[a]	248	0.17 (0.15)	186	0.17 (0.15)	62	0.19 (0.16)	-0.02 ± 0.02
CCI-web[a]	120	0.18 (0.15)	88	0.18 (0.15)	32	0.21 (0.16)	-0.03 ± 0.03
CCI-onsite[a]	128	0.16 (0.16)	98	0.16 (0.15)	30	0.17 (0.16)	-0.01 ± 0.04
Usual care[a]	79	0.15 (0.13)	59	0.14 (0.12)	20	0.17 (0.15)	-0.03 ± 0.03
CCI-web vs. CCI-onsite[b]		0.02 ± 0.02		0.01 ± 0.03		0.03 ± 0.04	
CCI-web vs. usual care[b]		0.03 ± 0.02		0.03 ± 0.03		0.03 ± 0.04	
CCI-onsite vs. usual care[b]		0.01 ± 0.02		0.02 ± 0.03		0.0 ± 0.04	
CCI-all vs. usual care[b]		0.02 ± 0.02		0.02 ± 0.02		0.02 ± 0.04	
**Hemoglobin A_{1c} (mmol·mol^{-1})**							
All	349	59.66 (17.16)	276	59.11 (16.62)	73	61.63 (18.91)	-26.04 ± 2.4
CCI-all education[a]	262	59.55 (16.4)	204	58.35 (15.3)	58	63.49 (19.57)	-28.66 ± 2.73
CCI-web[a]	126	58.68 (15.41)	98	57.69 (13.99)	28	61.85 (19.46)	-27.57 ± 3.94
CCI-onsite[a]	136	60.32 (17.38)	106	59.01 (16.51)	30	65.02 (19.89)	-29.65 ± 3.94
Usual care[a]	87	59.99 (19.24)	72	61.08 (19.89)	15	54.52 (14.87)	-16.97 ± 4.48
CCI-web vs. CCI-onsite[b]		-1.64 ± 2.08		-1.2 ± 2.19		-3.28 ± 5.14	
CCI-web vs. usual care[b]		-1.31 ± 2.51		-3.39 ± 2.73		7.32 ± 5.25	
CCI-onsite vs. usual care[b]		0.33 ± 2.51		-2.19 ± 2.84		10.6 ± 5.25*	
CCI-all vs. usual care[b]		-0.44 ± 2.3		-2.73 ± 2.62		8.96 ± 4.59*	
**Hemoglobin A_{1c} (%)**							
All	349	7.61 (1.57)	276	7.56 (1.52)	73	7.79 (1.73)	-0.23 ± 0.22
CCI-all education[a]	262	7.60 (1.50)	204	7.49 (1.4)	58	7.96 (1.79)	-0.47 ± 0.25
CCI-web[a]	126	7.52 (1.41)	98	7.43 (1.28)	28	7.81 (1.78)	-0.37 ± 0.36
CCI-onsite[a]	136	7.67 (1.59)	106	7.55 (1.51)	30	8.10 (1.82)	-0.56 ± 0.36

Usual care[a]	87	7.64 (1.76)	72	7.74 (1.82)	15	7.14 (1.36)	0.60 ± 0.41
CCI-web vs. CCI-onsite[b]		-0.15 ± 0.19		-0.11 ± 0.2		-0.3 ± 0.47	
CCI-web vs. usual care[b]		-0.12 ± 0.23		-0.31 ± 0.25		0.67 ± 0.48	
CCI-onsite vs. usual care[b]		0.03 ± 0.23		-0.2 ± 0.26		0.97 ± 0.48*	
CCI-all vs. usual care[b]		-0.04 ± 0.21		-0.25 ± 0.24		0.82 ± 0.42*	
**Fasting glucose (mmol·L^{-1})**							
All	344	8.86 (3.57)	273	8.78 (3.46)	71	9.18 (3.96)	-0.4 ± 0.52
CCI-all education[a]	258	8.92 (3.41)	202	8.8 (3.28)	56	9.36 (3.83)	-0.55 ± 0.56
CCI-web[a]	123	8.61 (3.12)	97	8.5 (3.05)	26	9.04 (3.41)	-0.54 ± 0.74
CCI-onsite[a]	135	9.2 (3.64)	105	9.08 (3.47)	30	9.63 (4.21)	-0.55 ± 0.84
Usual care[a]	86	8.67 (4.03)	71	8.71 (3.96)	15	8.5 (4.5)	0.21 ± 1.25
CCI-web vs. CCI-onsite[b]		-0.59 ± 0.42		-0.58 ± 0.46		-0.59 ± 1.02	
CCI-web vs. usual care[b]		-0.05 ± 0.52		-0.21 ± 0.56		0.54 ± 1.34	
CCI-onsite vs. usual care[b]		0.54 ± 0.54		0.38 ± 0.58		1.13 ± 1.39	
CCI-all vs. usual care[b]		0.25 ± 0.48		0.1 ± 0.52		0.86 ± 1.27	
**Insulin all (pmol·L^{-1})**							
All	327	199.25 (167.24)	245	199.81 (172.03)	82	197.59 (153.0)	2.22 ± 20.14
CCI-all education[a]	248	198.35 (165.85)	186	197.65 (167.17)	62	200.5 (163.21)	-2.85 ± 24.1
CCI-web[a]	120	168.07 (110.49)	88	175.99 (121.05)	32	146.26 (71.53)	29.72 ± 18.06
CCI-onsite[a]	128	226.68 (200.92)	98	217.03 (198.35)	30	258.28 (209.46)	-41.25 ± 43.2
Usual care[a]	79	202.17 (172.58)	59	206.68 (187.93)	20	188.77 (119.18)	17.99 ± 36.18
CCI-web vs. CCI-onsite[b]		-58.62 ± 20.42†		-41.04 ± 23.82		-112.02 ± 40.28†	
CCI-web vs. usual care[b]		-34.1 ± 21.88		-30.7 ± 27.64		-42.5 ± 29.52	
CCI-onsite vs. usual care[b]		24.59 ± 26.32		10.35 ± 31.6		69.59 ± 46.6	
CCI-all vs. usual care[b]		-3.82 ± 22.09		-9.1 ± 27.36		11.74 ± 33.75	
**Insulin, excluding exogenous users (pmol·L^{1})**							
All	219	202.86 (167.51)	160	202.79 (174.46)	59	203.0 (148.21)	-0.14 ± 23.75
CCI-all education[a]	176	204.04 (165.01)	135	203.42 (170.22)	41	205.99 (148.55)	-2.57 ± 27.43
CCI-web[a]	84	171.68 (100.98)	60	178.63 (110.36)	24	154.46 (71.53)	24.17 ± 20.42
CCI-onsite[a]	92	233.56 (203.0)	75	223.28 (204.6)	17	278.77 (195.43)	-55.49 ± 52.99
Usual care[a]	43	198.14 (179.18)	25	199.6 (199.81)	18	196.13 (151.61)	3.47 ± 53.62
CCI-web vs. CCI-onsite[b]		-61.81 ± 23.89†		-44.66 ± 27.57		-124.25 ± 49.59†	
CCI-web vs. usual care[b]		-26.39 ± 29.45		-20.97 ± 42.43		-41.67 ± 38.61	
CCI-onsite vs. usual care[b]		35.42 ± 34.59		23.68 ± 46.39		82.65 ± 59.45	
CCI-all vs. usual care[b]		5.9 ± 30.0		3.89 ± 42.57		9.86 ± 42.57	

**C-peptide (nmol·L^{-1})**							
All	326	1.43 (0.74)	244	1.44 (0.74)	82	1.42 (0.72)	0.02 ± 0.09
CCI-all education[a]	247	1.45 (0.71)	185	1.47 (0.72)	62	1.39 (0.69)	0.07 ± 0.1
CCI-web[a]	120	1.38 (0.68)	88	1.4 (0.74)	32	1.32 (0.46)	0.07 ± 0.11
CCI-onsite[a]	127	1.52 (0.73)	97	1.53 (0.69)	30	1.47 (0.87)	0.06 ± 0.17
Usual care[a]	79	1.38 (0.82)	59	1.35 (0.82)	20	1.49 (0.84)	-0.14 ± 0.22
CCI-web vs. CCI-onsite[b]		-0.14 ± 0.09		-0.13 ± 0.11		-0.15 ± 0.18	
CCI-web vs. usual care[b]		-0.01 ± 0.11		0.05 ± 0.13		-0.16 ± 0.21	
CCI-onsite vs. usual care[b]		0.13 ± 0.11		0.18 ± 0.13		-0.02 ± 0.24	
CCI-all vs. usual care[b]		0.07 ± 0.1		0.12 ± 0.12		-0.09 ± 0.21	
**HOMA-IR (insulin derived), all**							
All	322	11.52 (12.28)	235	11.22 (12.14)	87	12.34 (12.7)	-1.12 ± 1.57
CCI-all education[a]	244	11.8 (13.14)	179	11.19 (12.75)	65	13.48 (14.12)	-2.3 ± 1.99
CCI-web[a]	117	9.59 (8.37)	84	9.9 (9.26)	33	8.8 (5.54)	1.1 ± 1.4
CCI-onsite[a]	127	13.84 (16.11)	95	12.33 (15.14)	32	18.32 (18.23)	-5.99 ± 3.58
Usual care[a]	78	10.64 (9.12)	56	11.31 (10.05)	22	8.94 (6.03)	2.36 ± 1.86
CCI-web vs. CCI-onsite[b]		-4.25 ± 1.63†		-2.43 ± 1.85		-9.52 ± 3.36†	
CCI-web vs. usual care[b]		-1.05 ± 1.29		-1.4 ± 1.68		-0.15 ± 1.61	
CCI-onsite vs. usual care[b]		3.2 ± 1.76		1.02 ± 2.05		9.37 ± 3.47†	
CCI-all vs. usual care[b]		1.16 ± 1.33		-0.12 ± 1.65		4.54 ± 2.17	
**HOMA-IR (insulin derived), excluding exogenous users**							
All	215	11.30 (12.96)	154	10.74 (12.94)	61	12.72 (13.01)	-1.98 ± 1.97
CCI-all education[a]	172	11.77 (13.87)	129	11.00 (13.53)	43	14.09 (14.76)	-3.08 ± 2.55
CCI-web[a]	81	9.08 (7.16)	57	9.21 (7.82)	24	8.79 (5.39)	0.41 ± 1.51
CCI-onsite[a]	91	14.17 (17.54)	72	12.42 (16.65)	19	20.77 (19.66)	-8.35 ± 4.92
Usual care[a]	43	9.40 (8.25)	25	9.36 (9.39)	18	9.45 (6.61)	-0.09 ± 2.44
CCI-web vs. CCI-onsite[b]		-5.08 ± 2.00*		-3.22 ± 2.22		-11.98 ± 4.64†	
CCI-web vs. usual care[b]		-0.32 ± 1.49		-0.16 ± 2.14		-0.66 ± 1.91	
CCI-onsite vs. usual care[b]		4.76 ± 2.23*		3.06 ± 2.72		11.32 ± 4.77*	
CCI-all vs. usual care[b]		2.37 ± 1.64		1.64 ± 2.22		4.63 ± 2.74	
**HOMA-IR (C-peptide derived)**							
All	311	11.44 (7.17)	217	11.25 (6.59)	94	11.87 (8.38)	-0.63 ± 0.97
CCI-all education[a]	239	11.52 (7.15)	170	11.44 (6.26)	69	11.72 (9.04)	-0.28 ± 1.19
CCI-web[a]	115	10.52 (5.72)	79	10.75 (6.29)	36	10.01 (4.24)	0.74 ± 1.00
CCI-onsite[a]	124	12.45 (8.18)	91	12.04 (6.20)	33	13.59 (12.13)	-1.55 ± 2.21

Usual care[a]	72	11.16 (7.26)	47	10.56 (7.70)	25	12.29 (6.33)	-1.73 ± 1.69
CCI-web vs. CCI-onsite[b]		-1.93 ± 0.91*		-1.29 ± 0.96		-3.58 ± 2.23	
CCI-web vs. usual care[b]		-0.64 ± 1.01		0.19 ± 1.33		-2.28 ± 1.45	
CCI-onsite vs. usual care[b]		1.29 ± 1.13		1.48 ± 1.30		1.3 ± 2.46	
CCI-all vs. usual care[b]		0.36 ± 0.97		0.88 ± 1.22		-0.56 ± 1.67	
**Weight-clinic (kg)**							
All	340	113.85 (25.47)	253	113.06 (24.24)	87	116.14 (28.77)	-3.08 ± 3.44
CCI-all education[a]	257	116.51 (25.94)	184	115.42 (24.62)	73	119.25 (29.01)	-3.83 ± 3.85
CCI-web[a]	121	114.58 (27.08)	84	114.78 (25.87)	37	114.14 (30.01)	0.64 ± 5.68
CCI-onsite[a]	136	118.22 (24.86)	100	115.96 (23.63)	36	124.5 (27.36)	-8.54 ± 5.14
Usual care[a]	83	105.63 (22.15)	69	106.79 (22.18)	14	99.94 (21.86)	6.84 ± 6.42
CCI-web vs. CCI-onsite[b]		-3.64 ± 3.26		-1.18 ± 3.68		-10.36 ± 6.72	
CCI-web vs. usual care[b]		8.95 ± 3.46†		7.99 ± 3.89*		14.2 ± 7.65	
CCI-onsite vs. usual care[b]		12.58 ± 3.23§		9.17 ± 3.57†		24.55 ± 7.41‡	
CCI-all vs. usual care[b]		10.87 ± 2.92§		8.63 ± 3.23†		19.3 ± 6.76†	
**Weight-home scale (kg)**							
CCI-all education[a]	262	116.4 (26.31)	218	116.29 (25.25)	44	116.94 (31.37)	-0.64 ± 5.03
CCI-web[a]	126	114.52 (27.52)	104	116.05 (27.42)	22	107.26 (27.42)	8.8 ± 6.44
CCI-onsite[a]	136	118.14 (25.12)	114	116.51 (23.21)	22	126.61 (32.65)	-10.11 ± 7.29
CCI-web vs. CCI-onsite[b]		-3.62 ± 3.26		-0.45 ± 3.46		-19.36 ± 9.09*	
**BMI ($kg \cdot m^{-2}$)**							
All	340	39.52 (8.6)	253	39.13 (7.89)	87	40.66 (10.36)	-1.54 ± 1.22
CCI-all education[a]	257	40.43 (8.81)	184	39.87 (7.88)	73	41.82 (10.75)	-1.94 ± 1.39
CCI-web[a]	121	39.36 (9.12)	84	38.82 (7.81)	37	40.6 (11.6)	-1.79 ± 2.09
CCI-onsite[a]	136	41.37 (8.44)	100	40.76 (7.86)	36	43.06 (9.82)	-2.3 ± 1.82
Usual care[a]	83	36.72 (7.26)	69	37.14 (7.62)	14	34.66 (4.8)	2.48 ± 1.58
CCI-web vs. CCI-onsite[b]		-2.01 ± 1.1		-1.95 ± 1.16		-2.46 ± 2.51	
CCI-web vs. usual care[b]		2.64 ± 1.15*		1.68 ± 1.25		5.94 ± 2.3†	
CCI-onsite vs. usual care[b]		4.65 ± 1.08§		3.62 ± 1.21†		8.4 ± 2.08§	
CCI-all vs. usual care[b]		3.7 ± 0.97‡		2.73 ± 1.09†		7.15 ± 1.8§	
**Systolic blood pressure (mmHg)**							
All	339	131.44 (13.99)	254	131.51 (14.14)	85	131.22 (13.6)	0.29 ± 1.72
CCI-all education[a]	260	131.94 (14.09)	187	132.51 (14.54)	73	130.47 (12.84)	2.05 ± 1.84
CCI-web[a]	124	132.66 (14.15)	87	133.22 (14.78)	37	131.35 (12.63)	1.87 ± 2.61
CCI-onsite[a]	136	131.28 (14.06)	100	131.9 (14.38)	36	129.56 (13.16)	2.34 ± 2.62

Usual care[a]	79	129.8 (13.61)	67	128.72 (12.65)	12	135.83 (17.49)	-7.12 ± 5.28
CCI-web vs. CCI-onsite[b]		1.38 ± 1.75		1.32 ± 2.14		1.8 ± 3.02	
CCI-web vs. usual care[b]		2.86 ± 1.99		4.5 ± 2.21*		-4.48 ± 5.46	
CCI-onsite vs. usual care[b]		1.48 ± 1.95		3.18 ± 2.11		-6.28 ± 5.5	
CCI-all vs. usual care[b]		2.14 ± 1.76		3.8 ± 1.88*		-5.37 ± 5.27	
**Diastolic blood pressure (mmHg)**							
All	339	82.07 (8.4)	254	81.46 (8.04)	85	83.88 (9.2)	-2.42 ± 1.12*
CCI-all education[a]	260	82.09 (8.25)	187	81.59 (8.05)	73	83.37 (8.67)	-1.78 ± 1.17
CCI-web[a]	124	82.6 (8.85)	87	81.52 (8.27)	37	85.14 (9.74)	-3.62 ± 1.83*
CCI-onsite[a]	136	81.63 (7.67)	100	81.66 (7.9)	36	81.56 (7.09)	0.1 ± 1.42
Usual care[a]	79	82.0 (8.93)	67	81.1 (8.07)	12	87.0 (11.95)	-5.9 ± 3.59
CCI-web vs. CCI-onsite[b]		0.96 ± 1.03		-0.14 ± 1.19		3.58 ± 1.99	
CCI-web vs. usual care[b]		0.6 ± 1.28		0.41 ± 1.33		-1.86 ± 3.8	
CCI-onsite vs. usual care[b]		-0.37 ± 1.2		0.56 ± 1.26		-5.44 ± 3.65	
CCI-all vs. usual care[b]		0.09 ± 1.13		0.49 ± 1.15		-3.63 ± 3.6	
**Total cholesterol (mmol·L^{-1})**							
All	326	4.76 (1.09)	245	4.69 (1.09)	81	4.96 (1.1)	-0.28 ± 0.14*
CCI-all education[a]	247	4.76 (1.07)	186	4.68 (1.03)	61	4.99 (1.15)	-0.31 ± 0.17
CCI-web[a]	120	4.78 (1.1)	88	4.59 (1.06)	32	5.3 (1.07)	-0.72 ± 0.22‡
CCI-onsite[a]	127	4.73 (1.03)	98	4.76 (1.0)	29	4.65 (1.16)	0.11 ± 0.24
Usual care[a]	79	4.76 (1.19)	59	4.72 (1.26)	20	4.88 (0.93)	-0.16 ± 0.27
CCI-web vs. CCI-onsite[b]		0.04 ± 0.14		-0.17 ± 0.15		0.66 ± 0.29*	
CCI-web vs. usual care[b]		0.02 ± 0.17		-0.13 ± 0.2		0.42 ± 0.28	
CCI-onsite vs. usual care[b]		-0.03 ± 0.16		0.04 ± 0.19		-0.24 ± 0.3	
CCI-all vs. usual care[b]		-0.0 ± 0.15		-0.04 ± 0.18		0.11 ± 0.26	
**LDL-cholesterol (mmol·L^{-1})**							
All	302	102.27 (33.62)	220	100.15 (33.71)	82	107.99 (32.89)	-7.84 ± 4.28
CCI-all education[a]	232	102.51 (32.89)	172	100.08 (32.56)	60	109.47 (33.13)	-9.39 ± 4.94
CCI-web[a]	115	103.08 (35.61)	83	97.14 (35.58)	32	118.47 (31.22)	-21.32 ± 6.76†
CCI-onsite[a]	117	101.95 (30.13)	89	102.82 (29.4)	28	99.18 (32.75)	3.64 ± 6.93
Usual care[a]	70	101.50 (36.16)	48	100.38 (37.93)	22	103.95 (32.67)	-3.58 ± 8.86
CCI-web vs. CCI-onsite[b]		1.13 ± 4.33		-5.68 ± 5.00		19.29 ± 8.29*	
CCI-web vs. usual care[b]		1.58 ± 5.45		-3.23 ± 6.72		14.51 ± 8.89	
CCI-onsite vs. usual care[b]		0.45 ± 5.14		2.45 ± 6.30		-4.78 ± 9.32	
CCI-all vs. usual care[b]		1.01 ± 4.83		-0.29 ± 6.01		5.51 ± 8.17	

**Apo B (g·L^{-1})**							
All	327	1.06 (0.29)	245	1.04 (0.28)	82	1.1 (0.29)	-0.06 ± 0.04
CCI-all education[a]	248	1.05 (0.29)	186	1.03 (0.28)	62	1.1 (0.31)	-0.06 ± 0.04
CCI-web[a]	120	1.08 (0.3)	88	1.03 (0.29)	32	1.21 (0.3)	-0.18 ± 0.06†
CCI-onsite[a]	128	1.02 (0.27)	98	1.04 (0.27)	30	0.97 (0.26)	0.06 ± 0.06
Usual care[a]	79	1.07 (0.28)	59	1.06 (0.3)	20	1.11 (0.24)	-0.05 ± 0.07
CCI-web vs. CCI-onsite[b]		0.06 ± 0.04		-0.01 ± 0.04		0.24 ± 0.07‡	
CCI-web vs. usual care[b]		0.01 ± 0.04		-0.03 ± 0.05		0.11 ± 0.08	
CCI-onsite vs. usual care[b]		-0.05 ± 0.04		-0.02 ± 0.05		-0.13 ± 0.07	
CCI-all vs. usual care[b]		-0.02 ± 0.04		-0.02 ± 0.04		-0.01 ± 0.07	
**HDL-C (mmol·L^{-1})**							
All	326	1.06 (0.34)	245	1.06 (0.35)	81	1.06 (0.31)	0.0 ± 0.04
CCI-all education[a]	247	1.09 (0.35)	186	1.1 (0.36)	61	1.08 (0.32)	0.02 ± 0.05
CCI-web[a]	120	1.1 (0.33)	88	1.1 (0.36)	32	1.1 (0.24)	-0.0 ± 0.06
CCI-onsite[a]	127	1.09 (0.36)	98	1.1 (0.36)	29	1.05 (0.39)	0.04 ± 0.08
Usual care[a]	79	0.97 (0.29)	59	0.96 (0.29)	20	1.02 (0.29)	-0.06 ± 0.08
CCI-web vs. CCI-onsite[b]		0.01 ± 0.04		0.0 ± 0.05		0.05 ± 0.08	
CCI-web vs. usual care[b]		0.12 ± 0.04†		0.14 ± 0.05		0.08 ± 0.08	
CCI-onsite vs. usual care[b]		0.11 ± 0.05*		0.14 ± 0.05†		0.04 ± 0.1	
CCI-all vs. usual care[b]		0.12 ± 0.04†		0.14 ± 0.05†		0.06 ± 0.08	
**Triglycerides (mmol·L^{-1})**							
All	326	2.46 (2.66)	245	2.53 (2.97)	81	2.26 (1.35)	0.27 ± 0.24
CCI-all education[a]	247	2.23 (1.62)	186	2.27 (1.73)	61	2.11 (1.25)	0.15 ± 0.2
CCI-web[a]	120	2.14 (1.17)	88	2.16 (1.2)	32	2.1 (1.1)	0.06 ± 0.23
CCI-onsite[a]	127	2.31 (1.95)	98	2.36 (2.09)	29	2.13 (1.41)	0.23 ± 0.34
Usual care[a]	79	3.2 (4.53)	59	3.36 (5.17)	20	2.72 (1.56)	0.64 ± 0.76
CCI-web vs. CCI-onsite[b]		-0.16 ± 0.2		-0.2 ± 0.25		-0.03 ± 0.33	
CCI-web vs. usual care[b]		-1.05 ± 0.52*		-1.2 ± 0.69		-0.62 ± 0.4	
CCI-onsite vs. usual care[b]		-0.89 ± 0.54*		-1.0 ± 0.71		-0.59 ± 0.44	
CCI-all vs. usual care[b]		-0.97 ± 0.52*		-1.09 ± 0.68		-0.61 ± 0.38*	
**Total/HDL-cholesterol**							
All	326	4.88 (1.92)	245	4.84 (2.0)	81	4.99 (1.66)	-0.15 ± 0.22
CCI-all education[a]	247	4.72 (1.7)	186	4.65 (1.72)	61	4.93 (1.65)	-0.28 ± 0.25
CCI-web[a]	120	4.68 (1.54)	88	4.55 (1.58)	32	5.02 (1.36)	-0.47 ± 0.29
CCI-onsite[a]	127	4.76 (1.85)	98	4.74 (1.83)	29	4.84 (1.94)	-0.1 ± 0.41

Usual care[a]	79	5.37 (2.42)	59	5.44 (2.63)	20	5.17 (1.72)	0.27 ± 0.52
CCI-web vs. CCI-onsite[b]		-0.08 ± 0.22		-0.19 ± 0.25		0.19 ± 0.43	
CCI-web vs. usual care[b]		-0.69 ± 0.31*		-0.88 ± 0.38*		-0.15 ± 0.45	
CCI-onsite vs. usual care[b]		-0.61 ± 0.32		-0.7 ± 0.39		-0.33 ± 0.53	
CCI-all vs. usual care[b]		-0.65 ± 0.29*		-0.79 ± 0.36*		-0.24 ± 0.44	
**hsC-reactive protein (nmol·L^{-1})**							
All	334	82.19 (126.1)	263	86.0 (138.29)	71	68.29 (61.81)	17.71 ± 11.24
CCI-all education[a]	249	81.33 (138.0)	193	85.62 (153.05)	56	66.76 (62.1)	18.86 ± 13.81
CCI-web[a]	126	89.43 (183.72)	98	98.38 (98.38)	28	58.1 (58.1)	40.29 ± 22.1
CCI-onsite[a]	123	73.14 (63.53)	95	72.38 (72.38)	28	75.43 (75.43)	-3.05 ± 16.1
Usual care[a]	85	84.67 (82.1)	70	86.95 (86.95)	15	73.81 (73.81)	13.14 ± 19.14
CCI-web vs. CCI-onsite[b]		16.29 ± 17.33		25.91 ± 21.71		-17.33 ± 16.57	
CCI-web vs. usual care[b]		4.76 ± 18.67		11.43 ± 23.24		-15.71 ± 17.71	
CCI-onsite vs. usual care[b]		-11.52 ± 10.57		-14.57 ± 11.9		1.62 ± 22.0	
CCI-all vs. usual care[b]		-3.24 ± 12.48		-1.33 ± 15.05		-7.05 ± 18.19	
**ALT (μkat·L^{-1})**							
All	343	0.5 (0.37)	272	0.51 (0.39)	71	0.48 (0.27)	0.03 ± 0.04
CCI-all education[a]	257	0.51 (0.38)	201	0.52 (0.41)	56	0.47 (0.27)	0.05 ± 0.05
CCI-web[a]	123	0.53 (0.3)	97	0.56 (0.31)	26	0.45 (0.27)	0.11 ± 0.06
CCI-onsite[a]	134	0.49 (0.44)	104	0.49 (0.48)	30	0.49 (0.27)	0.0 ± 0.07
Usual care[a]	86	0.46 (0.33)	71	0.45 (0.34)	15	0.51 (0.29)	-0.05 ± 0.09
CCI-web vs. CCI-onsite[b]		0.04 ± 0.05		0.06 ± 0.06		-0.04 ± 0.07	
CCI-web vs. usual care[b]		0.07 ± 0.04		0.1 ± 0.05*		-0.06 ± 0.09	
CCI-onsite vs. usual care[b]		0.03 ± 0.05		0.04 ± 0.06		-0.02 ± 0.09	
CCI-all vs. usual care[b]		0.05 ± 0.04		0.07 ± 0.05		-0.04 ± 0.08	
**AST (μkat·L^{-1})**							
All	343	0.4 (0.27)	272	0.4 (0.3)	71	0.37 (0.15)	0.03 ± 0.03
CCI-all education[a]	257	0.4 (0.25)	201	0.41 (0.28)	56	0.36 (0.15)	0.04 ± 0.03
CCI-web[a]	123	0.42 (0.27)	97	0.44 (0.29)	26	0.35 (0.17)	0.09 ± 0.04
CCI-onsite[a]	134	0.37 (0.24)	104	0.37 (0.26)	30	0.37 (0.14)	0.0 ± 0.04
Usual care[a]	86	0.4 (0.32)	71	0.39 (0.35)	15	0.42 (0.16)	-0.03 ± 0.06
CCI-web vs. CCI-onsite[b]		0.05 ± 0.03		0.07 ± 0.04		-0.02 ± 0.04	
CCI-web vs. usual care[b]		0.02 ± 0.04		0.05 ± 0.05		-0.07 ± 0.05	
CCI-onsite vs. usual care[b]		-0.03 ± 0.04		-0.02 ± 0.05		-0.05 ± 0.05	
CCI-all vs. usual care[b]		-0.0 ± 0.04		0.01 ± 0.05		-0.06 ± 0.05	

**Alkaline phosphatase (µkat·L^{-1})**							
All	342	1.25 (0.39)	271	1.26 (0.4)	71	1.23 (0.36)	0.03 ± 0.05
CCI-all education[a]	256	1.24 (0.37)	200	1.24 (0.37)	56	1.23 (0.36)	0.01 ± 0.05
CCI-web[a]	123	1.22 (0.39)	97	1.23 (0.4)	26	1.19 (0.33)	0.04 ± 0.08
CCI-onsite[a]	133	1.25 (0.36)	103	1.25 (0.35)	30	1.27 (0.39)	-0.02 ± 0.08
Usual care[a]	86	1.29 (0.44)	71	1.31 (0.45)	15	1.22 (0.38)	0.09 ± 0.11
CCI-web vs. CCI-onsite[b]		-0.03 ± 0.05		-0.02 ± 0.05		-0.08 ± 0.1	
CCI-web vs. usual care[b]		-0.07 ± 0.06		-0.08 ± 0.07		-0.03 ± 0.12	
CCI-onsite vs. usual care[b]		-0.04 ± 0.06		-0.06 ± 0.06		0.05 ± 0.12	
CCI-all vs. usual care[b]		-0.05 ± 0.05		-0.07 ± 0.06		0.01 ± 0.11	
**Serum creatinine (µmol·L^{-1})**							
All	344	78.68 (21.22)	273	77.79 (20.33)	71	82.21 (24.75)	-4.42 ± 3.54
CCI-all education[a]	258	77.79 (21.22)	202	77.79 (20.33)	56	81.33 (24.75)	-3.54 ± 3.54
CCI-web[a]	123	76.91 (17.68)	97	76.91 (18.56)	26	76.02 (15.91)	0.88 ± 3.54
CCI-onsite[a]	135	79.56 (23.87)	105	77.79 (21.22)	30	85.75 (29.17)	-7.96 ± 6.19
Usual care[a]	86	80.44 (22.1)	71	78.68 (20.33)	15	86.63 (25.64)	-7.07 ± 7.07
CCI-web vs. CCI-onsite[b]		-2.65 ± 2.65		-0.88 ± 2.65		-10.61 ± 6.19	
CCI-web vs. usual care[b]		-3.54 ± 2.65		-1.77 ± 2.65		-10.61 ± 7.07	
CCI-onsite vs. usual care[b]		-0.88 ± 3.54		-0.88 ± 3.54		0.0 ± 8.84	
CCI-all vs. usual care[b]		-1.77 ± 2.65		-1.77 ± 2.65		-5.3 ± 7.07	
**BUN (mmol·L^{-1})**							
All	344	5.95 (2.31)	273	5.94 (2.08)	71	6.0 (3.06)	-0.06 ± 0.39
CCI-all education[a]	258	6.03 (2.34)	202	6.06 (2.15)	56	5.9 (2.96)	0.16 ± 0.42
CCI-web[a]	123	6.02 (1.71)	97	6.17 (1.81)	26	5.42 (1.14)	0.75 ± 0.29†
CCI-onsite[a]	135	6.04 (2.8)	105	5.96 (2.42)	30	6.32 (3.88)	-0.36 ± 0.75
Usual care[a]	86	5.73 (2.23)	71	5.59 (1.86)	15	6.38 (3.52)	-0.79 ± 0.94
CCI-web vs. CCI-onsite[b]		-0.02 ± 0.29		0.21 ± 0.3		-0.9 ± 0.74	
CCI-web vs. usual care[b]		0.29 ± 0.29		0.58 ± 0.29*		-0.95 ± 0.94	
CCI-onsite vs. usual care[b]		0.31 ± 0.34		0.37 ± 0.32		-0.06 ± 1.15	
CCI-all vs. usual care[b]		0.3 ± 0.28		0.47 ± 0.27		-0.47 ± 0.99	
**eGFR (mL·s^{-1}·m^{-2})**							
All	344	1.34 (0.23)	273	1.34 (0.22)	71	1.31 (0.26)	0.03 ± 0.03
CCI-all education[a]	258	1.34 (0.23)	202	1.35 (0.22)	56	1.33 (0.25)	0.02 ± 0.04
CCI-web[a]	123	1.36 (0.21)	97	1.37 (0.2)	26	1.35 (0.23)	0.02 ± 0.05
CCI-onsite[a]	135	1.33 (0.24)	105	1.33 (0.24)	30	1.32 (0.26)	0.01 ± 0.05

Usual care[a]	86	1.32 (0.23)	71	1.34 (0.22)	15	1.26 (0.28)	0.08 ± 0.08
CCI-web vs. CCI-onsite[b]		0.04 ± 0.05		0.04 ± 0.03		0.03 ± 0.07	
CCI-web vs. usual care[b]		0.04 ± 0.03		0.03 ± 0.03		0.09 ± 0.09	
CCI-onsite vs. usual care[b]		0.0 ± 0.03		-0.01 (0.01)		0.06 ± 0.09	
CCI-all vs. usual care[b]		0.02 ± 0.03		0.02 ± 0.03		0.03 ± 0.08	
**Anion gap (mmol·L^{-1})**							
All	343	6.86 (1.7)	272	6.82 (1.73)	71	6.99 (1.6)	-0.16 ± 0.22
CCI-all education[a]	257	6.83 (1.67)	201	6.79 (1.7)	56	6.98 (1.53)	-0.19 ± 0.24
CCI-web[a]	123	6.93 (1.71)	97	6.97 (1.66)	26	6.77 (1.92)	0.2 ± 0.41
CCI-onsite[a]	134	6.75 (1.63)	104	6.63 (1.74)	30	7.17 (1.09)	-0.54 ± 0.26*
Usual care[a]	86	6.93 (1.82)	71	6.92 (1.82)	15	7.0 (1.89)	-0.08 ± 0.53
CCI-web vs. CCI-onsite[b]		0.18 ± 0.21		0.34 ± 0.24		-0.4 ± 0.43	
CCI-web vs. usual care[b]		0.0 ± 0.25		0.05 ± 0.27		-0.23 ± 0.62	
CCI-onsite vs. usual care[b]		-0.18 ± 0.24		-0.29 ± 0.27		0.17 ± 0.53	
CCI-all vs. usual care[b]		-0.1 ± 0.22		-0.12 ± 0.25		-0.02 ± 0.53	
**CO_2 (mmol·L^{-1})**							
All	344	27.86 (2.65)	273	27.97 (2.58)	71	27.42 (2.91)	0.55 ± 0.38
CCI-all education[a]	258	27.78 (2.55)	202	27.95 (2.37)	56	27.18 (3.06)	0.77 ± 0.44
CCI-web[a]	123	27.9 (2.37)	97	27.97 (2.15)	26	27.65 (3.1)	0.32 ± 0.65
CCI-onsite[a]	135	27.67 (2.7)	105	27.92 (2.56)	30	26.77 (3.01)	1.16 ± 0.6
Usual care[a]	86	28.1 (2.95)	71	28.06 (3.11)	15	28.33 (2.09)	-0.28 ± 0.65
CCI-web vs. CCI-onsite[b]		0.24 ± 0.32		0.05 ± 0.33		0.89 ± 0.82	
CCI-web vs. usual care[b]		-0.2 ± 0.38		-0.09 ± 0.43		-0.68 ± 0.81	
CCI-onsite vs. usual care[b]		-0.44 ± 0.39		-0.13 ± 0.45		-1.57 ± 0.77*	
CCI-all vs. usual care[b]		-0.33 ± 0.36		-0.11 ± 0.4		-1.15 ± 0.68	
**Uric acid (µmol·L^{-1})**							
All	346	344.42 (86.85)	273	343.82 (86.25)	73	346.2 (91.01)	-2.38 ± 11.9
CCI-all education[a]	261	347.99 (86.85)	202	348.58 (86.25)	59	346.2 (89.82)	2.38 ± 13.09
CCI-web[a]	126	355.13 (87.44)	98	361.07 (89.82)	28	333.71 (77.93)	27.36 ± 17.25
CCI-onsite[a]	135	341.44 (85.66)	104	336.69 (80.9)	31	357.5 (99.34)	-20.82 ± 19.63
Usual care[a]	85	333.12 (87.44)	71	330.74 (85.66)	14	345.01 (98.75)	-14.28 ± 28.55
CCI-web vs. CCI-onsite[b]		13.68 ± 10.71		24.98 ± 11.9		-23.79 ± 23.2	
CCI-web vs. usual care[b]		22.01 ± 12.49		30.34 ± 13.68*		-11.3 ± 30.34	
CCI-onsite vs. usual care[b]		7.73 ± 11.9		5.35 ± 13.09*		12.49 ± 32.12	
CCI-all vs. usual care[b]		14.87 ± 10.71		17.25 ± 11.9		1.19 ± 29.15	

**TSH (mIU·L^{-1})**							
All	344	2.23 (1.62)	270	2.25 (1.65)	74	2.17 (1.51)	0.08 ± 0.2
CCI-all education[a]	259	2.32 (1.74)	200	2.31 (1.79)	59	2.38 (1.55)	-0.07 ± 0.24
CCI-web[a]	126	2.37 (1.91)	98	2.46 (2.11)	28	2.07 (0.91)	0.38 ± 0.27
CCI-onsite[a]	133	2.28 (1.56)	102	2.16 (1.42)	31	2.65 (1.93)	-0.49 ± 0.37
Usual care[a]	85	1.97 (1.16)	70	2.09 (1.16)	15	1.38 (1.03)	0.71 ± 0.3*
CCI-web vs. CCI-onsite[b]		0.09 ± 0.22		0.29 ± 0.26		-0.58 ± 0.39	
CCI-web vs. usual care[b]		0.4 ± 0.21		0.36 ± 0.25		0.69 ± 0.32	
CCI-onsite vs. usual care[b]		0.31 ± 0.19		0.07 ± 0.2		1.27 ± 0.44†	
CCI-all vs. usual care[b]		0.36 ± 0.17*		0.21 ± 0.19		1.0 ± 0.33†	
**Free T4 (pmol·L^{-1})**							
All	346	11.71 (2.7)	273	11.71 (2.83)	73	11.46 (2.19)	0.26 ± 0.26
CCI-all education[a]	260	11.84 (2.19)	202	11.84 (2.32)	58	11.58 (2.19)	0.26 ± 0.39
CCI-web[a]	125	11.84 (2.06)	98	11.84 (1.93)	27	12.1 (2.06)	-0.26 ± 0.51
CCI-onsite[a]	135	11.84 (2.45)	104	11.97 (2.57)	31	11.2 (2.19)	0.77 ± 0.51
Usual care[a]	86	11.33 (3.73)	71	11.33 (3.86)	15	10.94 (2.32)	0.39 ± 0.77
CCI-web vs. CCI-onsite[b]		0.0 ± 0.26		-0.26 ± 0.26		0.77 ± 0.51	
CCI-web vs. usual care[b]		0.51 ± 0.39		0.51 ± 0.51		1.03 ± 0.77	
CCI-onsite vs. usual care[b]		0.51 ± 0.39		0.64 ± 0.51		0.26 ± 0.77	
CCI-all vs. usual care[b]		0.51 ± 0.39		0.51 ± 0.51		0.64 ± 0.64	
**Any diabetes medication, excluding metformin (%)**							
All	349	59.31 ± 2.63	291	58.76 ± 2.89	58	60.07 ± 6.37	-3.31 ± 6.99
CCI-all education[a]	262	56.87 ± 3.06	218	55.50 ± 3.37	44	63.64 ± 7.25	-8.13 ± 8.00
CCI-web[a]	126	57.14 ± 4.41	104	56.73 ± 4.86	22	59.09 ± 10.48	-2.36 ± 11.55
CCI-onsite[a]	136	56.62 ± 4.25	114	54.39 ± 4.66	22	68.18 ± 9.93	-13.80± 10.97†
Usual care[a]	87	66.67 ± 5.05	73	68.49 ± 5.44	14	57.14 ± 13.23	11.35 ± 14.32
CCI-web vs. CCI-onsite[b]		0.53 ± 6.12		2.34 ± 6.74		-9.09 ± 14.44	
CCI-web vs. usual care[b]		-9.52 ± 6.71		-11.76 ± 7.29		1.95 ± 16.88	
CCI-onsite vs. usual care[b]		-10.05 ± 6.60		-14.11 ± 7.16*		11.04 ± 16.54	
CCI-all vs. usual care[b]		-9.80 ± 5.91		-12.99± 6.39*		6.49 ± 15.08	
**Sulfonylurea (%)**							
All	349	23.78 ± 2.28	291	24.05 ± 2.51	58	22.41 ± 5.48	1.64 ± 6.02
CCI-all education[a]	262	23.66 ± 2.63	218	24.31 ± 2.91	44	20.45 ± 6.08	3.86 ± 6.74
CCI-web[a]	126	23.81 ± 3.79	104	25.96 ± 4.3	22	13.64 ± 7.32	12.33 ± 8.49
CCI-onsite[a]	136	23.53 ± 3.64	114	22.81 ± 3.93	22	27.27 ± 9.5	-4.47 ± 10.28

Usual care[a]	87	24.14 ± 4.59	73	23.29 ± 4.95	14	28.57 ± 12.07	-5.28 ± 13.05
CCI-web vs. CCI-onsite[b]		0.28 ± 5.26		3.15 ± 5.82		-13.64 ± 11.99	
CCI-web vs. usual care[b]		-0.33 ± 5.95		2.67 ± 6.55		-14.94 ± 14.12	
CCI-onsite vs. usual care[b]		-0.61 ± 5.85		-0.48 ± 6.32		-1.30 ± 15.36	
CCI-all vs. usual care[b]		-0.48 ± 5.29		1.02 ± 5.74		-8.12± 13.52	
**Insulin (%)**							
All	349	33.81 ± 2.53	296	34.12 ± 2.76	53	32.08 ± 6.41	2.04 ± 6.98
CCI-all education[a]	262	29.77 ± 2.82	218	28.44 ± 3.06	44	36.36 ± 7.25	-7.92 ± 7.87
CCI-web[a]	126	28.57 ± 4.02	104	27.88 ± 4.4	22	31.82 ± 9.93	-3.93 ± 10.86
CCI-onsite[a]	136	30.88 ± 3.96	114	28.95 ± 4.25	22	40.91 ± 10.48	-11.96 ± 11.31
Usual care[a]	87	45.98 ± 5.34	78	50.0 ± 5.66	9	11.11 ± 10.48	38.89 (1.91)‡
CCI-web vs. CCI-onsite[b]		-2.31 ± 5.65		-1.06 ± 6.11		-9.09 ± 14.44	
CCI-web vs. usual care[b]		-17.41 ± 6.69†		-22.12 ± 7.17†		20.71 ± 14.43	
CCI-onsite vs. usual care[b]		-15.09 ± 6.65*		-21.05 ± 7.08†		29.8 ± 14.82*	
CCI-all vs. usual care[b]		-16.21 ± 6.04†		-21.56 ± 6.43‡		25.25 ± 12.74*	
**Thiazolidinedione (%)**							
All	349	1.43 ± 0.64	291	1.72 ± 0.76	58	0.0 ± 0.0	1.72 ± 0.76*
CCI-all education[a]	262	1.53 ± 0.76	218	1.83 ± 0.91	44	0.0 ± 0.0	1.83 ± 0.91*
CCI-web[a]	126	2.38 ± 1.36	104	2.88 ± 1.64	22	0.0 ± 0.0	2.88 ± 1.64
CCI-onsite[a]	136	0.74 ± 0.73	114	0.88 ± 0.87	22	0.0 ± 0.0	0.88 ± 0.87
Usual care[a]	87	1.15 ± 1.14	73	1.37 ± 1.36	14	0.0 ± 0.0	1.37 ± 1.36
CCI-web vs. CCI-onsite[b]		1.65 ± 1.54		2.01 ± 1.86		0.0 ± 0.0	
CCI-web vs. usual care[b]		1.23 ± 1.78		1.51 ± 2.13		0.0 ± 0.0	
CCI-onsite vs. usual care[b]		-0.41 ± 1.36		-0.49 ± 1.62		0.0 ± 0.0	
CCI-all vs. usual care[b]		0.38 ± 1.37		0.46 ± 1.64		0.0 ± 0.0	
**SGLT-2 (%)**							
All	349	11.17 ± 1.69	291	11.68 ± 1.88	58	8.62 ± 3.69	3.06 ± 4.14
CCI-all education[a]	262	10.31 ± 1.88	218	10.55 ± 2.08	44	9.09 ± 4.33	1.46 ± 4.81
CCI-web[a]	126	7.14 ± 2.29	104	7.69 ± 2.61	22	4.55 ± 4.44	3.15 ± 5.15
CCI-onsite[a]	136	13.24 ± 2.91	114	13.16 ± 3.17	22	13.64 ± 7.32	-0.48 ± 7.97
Usual care[a]	87	13.79 ± 3.7	73	15.07 ± 4.19	14	7.14 ± 6.88	7.93 ± 8.06
CCI-web vs. CCI-onsite[b]		-6.09 ± 3.7		-5.47 ± 4.1		-9.09 ± 8.56	
CCI-web vs. usual care[b]		-6.65 ± 4.35		-7.38 ± 4.94		-2.60 ± 8.19	
CCI-onsite vs. usual care[b]		-0.56 ± 4.7		-1.91 ± 5.25		6.49 ± 10.05	
CCI-all vs. usual care[b]		-3.48 ± 4.15		-4.52 ± 4.68		1.95 ± 8.13*	

**DPP-4 (%)**							
All	349	9.46 ± 1.57	291	9.62 ± 1.73	58	8.62 ± 3.69	1.00 ± 4.07
CCI-all education[a]	262	9.92 ± 1.85	218	10.09 ± 2.04	44	9.09 ± 4.33	1.0 ± 4.79
CCI-web[a]	126	10.32 ± 2.71	104	9.62 ± 2.89	22	13.64 ± 7.32	-4.02 ± 7.87
CCI-onsite[a]	136	9.56 ± 2.52	114	10.53 ± 2.87	22	4.55 ± 4.44	5.98 ± 5.29
Usual care[a]	87	8.05 ± 2.92	73	8.22 ± 3.21	14	7.14 ± 6.88	1.08 ± 7.60
CCI-web vs. CCI-onsite[b]		0.76 ± 3.7		-0.91 ± 4.08		9.09 ± 8.56	
CCI-web vs. usual care[b]		2.27 ± 3.98		1.40 ± 4.32		6.49 ± 10.05	
CCI-onsite vs. usual care[b]		1.51 ± 3.85		2.31 ± 4.31		-2.60 ± 8.19	
CCI-all vs. usual care[b]		1.87 ± 3.45		1.87 ± 3.81		1.95 ± 8.13	
**GLP-1 (%)**							
All	349	13.75 ± 1.84	291	13.75 ± 2.02	58	13.79 ± 4.53	-0.05 ± 4.96
CCI-all education[a]	262	13.36 ± 2.1	218	12.84 ± 2.27	44	15.91 ± 5.51	-3.07 ± 5.96
CCI-web[a]	126	12.7 ± 2.97	104	11.54 ± 3.13	22	18.18 ± 8.22	-6.64 ± 8.8
CCI-onsite[a]	136	13.97 ± 2.97	114	14.04 ± 3.25	22	13.64 ± 7.32	0.4 ± 8.01
Usual care[a]	87	14.94 ± 3.82	73	16.44 ± 4.34	14	7.14 ± 6.88	9.30 ± 8.14
CCI-web vs. CCI-onsite[b]		-1.27 ± 4.2		-2.5 ± 4.52		4.55 ± 11.01	
CCI-web vs. usual care[b]		-2.24 ± 4.84		-4.90 ± 5.35		11.04 ± 10.72	
CCI-onsite vs. usual care[b]		-0.97 ± 4.84		-2.40 ± 5.42		6.49 ± 10.05	
CCI-all vs. usual care[b]		-1.58 ± 4.36		-3.59 ± 4.89		8.77 ± 8.82	
**Metformin (%)**							
All	349	68.77 ± 2.48	291	69.07 ± 2.71	58	67.24 ± 6.16	1.83 ± 6.73
CCI-all education[a]	262	71.37 ± 2.79	218	71.56 ± 3.06	44	70.45 ± 6.88	1.11 ± 7.53
CCI-web[a]	126	69.84 ± 4.09	104	71.15 ± 4.44	22	63.64 ± 10.26	7.52 ± 11.18
CCI-onsite[a]	136	72.79 ± 3.82	114	71.93 ± 4.21	22	77.27 ± 8.93	-5.34 ± 9.88
Usual care[a]	87	60.92 ± 5.23	73	61.64 ± 5.69	14	57.14 ± 13.23	4.50 ± 14.40
CCI-web vs. CCI-onsite[b]		-2.95 ± 5.59		-0.78 ± 6.12		-13.64 ± 13.6	
CCI-web vs. usual care[b]		8.92 ± 6.64		9.51 ± 7.22		6.49 ± 16.74	
CCI-onsite vs. usual care[b]		11.87 ± 6.48		10.29 ± 7.08		20.13 ± 15.96	
CCI-all vs. usual care[b]		10.45 ± 5.93		9.92 ± 6.46		13.31 ± 14.91	
**Statin (%)**							
All	349	52.15 ± 2.67	291	52.58 ± 2.93	58	50.0 ± 6.57	2.58 ± 7.19
CCI-all education[a]	262	50.0 ± 3.09	218	51.83 ± 3.38	44	40.91 ± 7.41	10.93 ± 8.15
CCI-web[a]	126	47.62 ± 4.45	104	50.96 ± 4.9	22	31.82 ± 9.93	19.14 ± 11.07
CCI-onsite[a]	136	52.21 ± 4.28	114	52.63 ± 4.68	22	50.0 ± 10.66	2.63 ± 11.64

Usual care[a]	87	58.62 ± 5.28	73	54.79 ± 5.83	14	78.57 ± 10.97	-23.78 ± 12.42
CCI-web vs. CCI-onsite[b]		-4.59 ± 6.18		-1.67 ± 6.77		-18.18 ± 14.57	
CCI-web vs. usual care[b]		-11.0 ± 6.9		-3.83 ± 7.61		-46.75 ± 14.79†	
CCI-onsite vs. usual care[b]		-6.41 ± 6.8		-2.16 ± 7.47		-28.57 ± 15.29	
CCI-all vs. usual care[b]		-8.62 ± 6.12		-2.96 ± 6.74		-37.66 ± 13.24†	
**Blood pressure medication (%)**							
All	349	63.61 ± 2.58	291	63.92 ± 2.82	58	62.07 ± 6.37	1.85 ± 6.97
CCI-all education[a]	262	67.18 ± 2.90	218	68.35 ± 3.15	44	61.36 ± 7.34	6.98 ± 7.99
CCI-web[a]	126	65.08 ± 4.25	104	65.38 ± 4.67	22	63.64 ± 10.26	1.75 ± 11.27
CCI-onsite[a]	136	69.12 ± 3.96	114	71.05 ± 4.25	22	59.09 ± 10.48	11.96 ± 11.31
Usual care[a]	87	52.87 ± 5.35	73	50.68 ± 5.85	14	64.29 ± 12.81	-13.60 ± 14.08
CCI-web vs. CCI-onsite[b]		-4.04 ± 5.81		-5.67 ± 6.31		4.55 ± 14.67	
CCI-web vs. usual care[b]		12.21 ± 6.83		14.70 ± 7.48*		-0.65 ± 16.41	
CCI-onsite vs. usual care[b]		16.24 ± 6.66*		20.37 ± 7.23†		-5.19 ± 16.55	
CCI-all vs. usual care[b]		14.30 ± 6.09*		17.66 ± 6.65†		-2.92 ± 14.76	
**ACE or ARB (%)**							
All	349	26.65 ± 2.37	291	25.09 ± 2.54	58	34.48 ± 6.24	-9.4 ± 6.74
CCI-all education[a]	262	29.39 ± 2.81	218	27.98 ± 3.04	44	36.36 ± 7.25	-8.38 ± 7.86
CCI-web[a]	126	32.54 ± 4.17	104	30.77 ± 4.53	22	40.91 ± 10.48	-10.14 ± 11.42
CCI-onsite[a]	136	26.47 ± 3.78	114	25.44 ± 4.08	22	31.82 ± 9.93	-6.38 ± 10.74
Usual care[a]	87	18.39 ± 4.15	73	16.44 ± 4.34	14	28.57 ± 12.07	-12.13 ± 12.83
CCI-web vs. CCI-onsite[b]		6.07 ± 5.63		5.33 ± 6.09		9.09 ± 14.44	
CCI-web vs. usual care[b]		14.15 ± 5.89*		14.33 ± 6.27*		12.34 ± 15.99	
CCI-onsite vs. usual care[b]		8.08 ± 5.62		9.0 ± 5.95		3.25 ± 15.63	
CCI-all vs. usual care[b]		11.0 ± 5.02*		11.54 ± 5.3*		7.79 ± 14.08	
**Diuretics (%)**							
All	349	38.11 ± 2.6	291	37.11 ± 2.83	58	43.1 ± 6.5	-5.99 ± 7.09
CCI-all education[a]	262	40.84 ± 3.04	218	41.28 ± 3.33	44	38.64 ± 7.34	2.65 ± 8.06
CCI-web[a]	126	40.48 ± 4.37	104	41.35 ± 4.83	22	36.36 ± 10.26	4.98 ± 11.34
CCI-onsite[a]	136	41.18 ± 4.22	114	41.23 ± 4.61	22	40.91 ± 10.48	0.32 ± 11.45
Usual care[a]	87	29.89 ± 4.91	73	24.66 ± 5.04	14	57.14 ± 13.23	-32.49 ± 14.16*
CCI-web vs. CCI-onsite[b]		-0.7 ± 6.08		0.12 ± 6.68		-4.55 ± 14.67	
CCI-web vs. usual care[b]		10.59 ± 6.57		16.69 ± 6.98*		-20.78 ± 16.74	
CCI-onsite vs. usual care[b]		11.29 ± 6.47		16.57 ± 6.83*		-16.23 ± 16.88	
CCI-all vs. usual care[b]		10.95 ± 5.77		16.63 ± 6.05†		-18.51 ± 15.13	

[a]Mean and standard deviations for continuous variables, percents and standard errors for categorical variables
[b]Difference between means or percentages ± 1 standard error of the difference. Significant baseline difference between means or percentages at 0.05>P≥0.01 (*); 0.01>P≥0.001 (†); 0.001>P≥0.0001 (‡); and P<0.0001 (§).

Table S2. Mean changes in biomarkers between baseline and one-year for participants receiving the CCI and UC.

	Completers						All starters (Dropouts imputed)[d]		
			Unadjusted		Adjusted for baseline[c]			Unadjusted	
	N	Difference (SD) or ±SE	One Year	Signif-icance[e]	Difference ±SE	Signif-icance[e]	Difference ±SE	One Year	Signif-Icance[e]
**Beta-hydroxybutyrate (mmol·L^{-1})**									
CCI-all education[a]	186	0.14 (0.36)	0.31 (0.35)	$2.2x10^{-5}$	0.13 ± 0.02	$2.8x10^{-7}$	0.12 ± 0.02	0.3 ± 0.02	$5.8x10^{-7}$
CCI-web[a]	88	0.14 (0.37)	0.32 (0.36)	0.0003	0.14 ± 0.04	0.0001	0.13 ± 0.04	0.31 ± 0.04	0.0004
CCI-onsite[a]	98	0.13 (0.36)	0.29 (0.34)	0.0002	0.12 ± 0.04	0.0007	0.12 ± 0.03	0.28 ± 0.03	0.0004
Usual care[a]	59	0.04 (0.23)	0.18 (0.21)	0.24	0.06 ± 0.05	0.18	0.03 ± 0.04	0.18 ± 0.03	0.38
CCI-web vs. CCI-onsite[b]		0.01 ± 0.05		0.87	0.02 ± 0.05	0.73	0.01 ± 0.05		0.86
CCI-web vs. usual care[b]		0.11 ± 0.05		0.03	0.07 ± 0.06	0.22	0.1 ± 0.05		0.06
CCI-onsite vs. usual care[b]		0.1 ± 0.05		0.04	0.06 ± 0.06	0.37	0.09 ± 0.05		0.08
CCI-all vs. usual care[b]		0.1 (0.0)		0.01	0.06 ± 0.05	0.24	0.09 ± 0.04		0.04
**Hemoglobin A_{1c} (mmol·mol^{-1})**									
CCI-all education[a]	204	-14.1 (14.43)	44.25 (10.28)	$<10^{-16}$	-14.43 ± 0.98	$<10^{-16}$	-14.21 ± 0.98	45.23 ± 0.77	$<10^{-16}$
CCI-web[a]	98	-12.9 (12.24)	44.79 (9.29)	$<10^{-16}$	-13.12 ± 1.42	$<10^{-16}$	-13.23 ± 1.2	45.45 ± 1.09	$<10^{-16}$
CCI-onsite[a]	106	-15.3 (16.07)	43.7 (11.15)	$<10^{-16}$	-15.63 ± 1.42	$<10^{-16}$	-15.19 ± 1.42	45.12 ± 1.09	$<10^{-16}$
Usual care[a]	72	2.19 (14.76)	63.27 (19.89)	0.21	2.4 ± 1.75	0.17	2.19 ± 1.64	62.18 ± 2.08	0.18
CCI-web vs. CCI-onsite[b]		2.3 ± 1.97		0.24	2.4 ± 2.08	0.23	2.08 ± 1.86		0.28
CCI-web vs. usual care[b]		-15.08 ± 2.19		$1.5x10^{-12}$	-15.63 ± 2.3	$1.1x10^{-11}$	-15.3 ± 2.08		$3.8x10^{-14}$
CCI-onsite vs. usual care[b]		-17.49 ± 2.3		$8.7x10^{-14}$	-18.04 ± 2.3	$6.7x10^{-15}$	-17.38 ± 2.19		$6.7x10^{-16}$
CCI-all vs. usual care[b]		-16.29 ± 1.97		$4.4x10^{-16}$	-16.83 ± 2.08	$4.4x10^{-16}$	-16.4 ± 1.86		$<10^{-16}$
**Hemoglobin A_{1c} (%)**									
CCI-all education[a]	204	-1.29 (1.32)	6.20 (0.94)	$<10^{-16}$	-1.32 ± 0.09	$<10^{-16}$	-1.30 ± 0.09	6.29 ± 0.07	$<10^{-16}$
CCI-web[a]	98	-1.18 (1.12)	6.25 (0.85)	$<10^{-16}$	-1.20 ± 0.13	$<10^{-16}$	-1.21 ± 0.11	6.31 ± 0.1	$<10^{-16}$
CCI-onsite[a]	106	-1.40 (1.47)	6.15 (1.02)	$<10^{-16}$	-1.43 ± 0.13	$<10^{-16}$	-1.39 ± 0.13	6.28 ± 0.1	$<10^{-16}$
Usual care[a]	72	0.20 (1.35)	7.94 (1.82)	0.21	0.22 ± 0.16	0.17	0.20 ± 0.15	7.84 ± 0.19	0.18
CCI-web vs. CCI-onsite[b]		0.21 ± 0.18		0.24	0.22 ± 0.19	0.23	0.19 ± 0.17		0.28
CCI-web vs. usual care[b]		-1.38 ± 0.20		$1.5x10^{-12}$	-1.43 ± 0.21	$1.1x10^{-11}$	-1.40 ± 0.19		$3.8x10^{-14}$
CCI-onsite vs. usual care[b]		-1.60 ± 0.21		$8.7x10^{-14}$	-1.65 ± 0.21	$6.7x10^{-15}$	-1.59 ± 0.20		$6.7x10^{-16}$
CCI-all vs. usual care[b]		-1.49 ± 0.18		$4.4x10^{-16}$	-1.54 ± 0.19	$4.4x10^{-16}$	-1.50 ± 0.17		$<10^{-16}$
**Fasting glucose (mmol·L^{-1})**									
CCI-all education[a]	202	-1.96 (3.2)	6.84 (1.87)	$<10^{-16}$	-2.02 ± 0.26	$6.0x10^{-15}$	-1.95 ± 0.23	6.98 ± 0.17	$<10^{-16}$

CCI-web[a]	97	-1.87 (2.99)	6.63 (1.42)	6.4×10^{-10}	-1.94 ± 0.37	1.8×10^{-7}	-1.92 ± 0.31	6.66 ± 0.21	3.7×10^{-10}
CCI-onsite[a]	105	-2.04 (3.41)	7.04 (2.19)	8.5×10^{-10}	-2.1 ± 0.36	7.1×10^{-9}	-1.98 ± 0.34	7.27 ± 0.25	4.4×10^{-9}
Usual care[a]	71	0.59 (4.59)	9.3 (4.74)	0.28	0.81 ± 0.45	0.07	0.63 ± 0.49	9.29 ± 0.49	0.2
CCI-web vs. CCI-onsite[b]		0.16 ± 0.45		0.71	0.16 ± 0.52	0.76	0.06 ± 0.46		0.9
CCI-web vs. usual care[b]		-2.47 ± 0.62		7.5×10^{-5}	-2.75 ± 0.59	2.6×10^{-6}	-2.55 ± 0.58		1.1×10^{-5}
CCI-onsite vs. usual care[b]		-2.63 ± 0.64		3.7×10^{-5}	-2.91 ± 0.59	8.2×10^{-7}	-2.6 ± 0.6		1.3×10^{-5}
CCI-all vs. usual care[b]		-2.55 ± 0.59		1.5×10^{-5}	-2.83 ± 0.53	7.9×10^{-8}	-2.58 ± 0.54		2.1×10^{-6}
**Insulin, all (pmol·L^{-1})**									
CCI-all education[a]	186	-75.01 (178.49)	122.58 (169.6)	9.9×10^{-9}	-91.4 ± 12.15	5.5×10^{-14}	-73.62 ± 12.5	126.26 ± 12.5	4.3×10^{-9}
CCI-web[a]	88	-52.16 (202.59)	123.83 (212.38)	0.02	-74.66 ± 18.2	4.1×10^{-5}	-49.87 ± 19.38	118.9 ± 19.52	0.01
CCI-onsite[a]	98	-95.56 (151.89)	121.47 (120.08)	4.7×10^{-10}	-105.15 ± 16.53	2.2×10^{-10}	-95.56 ± 15.9	133.14 ± 15.77	2.0×10^{-9}
Usual care[a]	59	12.15 (210.23)	218.91 (239.46)	0.66	36.88 ± 29.66	0.21	5.97 ± 24.52	206.27 ± 26.11	0.81
CCI-web vs. CCI-onsite[b]		43.41 ± 26.46		0.1	30.49 ± 24.79	0.22	45.7 ± 25.07		0.07
CCI-web vs. usual care[b]		-64.31 ± 34.86		0.07	-111.54 ± 34.86	0.001	-55.84 ± 31.25		0.07
CCI-onsite vs. usual care[b]		-107.72 ± 31.39		0.0006	-142.03 ± 34.38	8.1×10^{-5}	-101.54 ± 29.24		0.0005
CCI-all vs. usual care[b]		-87.23 (29.86)		0.004	-127.58 ± 32.43	0.0009	-79.59 ± 27.5		0.004
**Insulin, excluding exogenous users (pmol·L^{-1})**									
CCI-all education[a]	135	-90.49 (118.34)	112.93 (99.94)	$<10^{-16}$	-91.4 ± 12.15	6.0×10^{-14}	-88.41 ± 10.9	112.16 ± 8.68	6.7×10^{-16}
CCI-web[a]	60	-76.19 (77.09)	102.44 (65.14)	1.9×10^{-14}	-74.66 ± 18.2	4.1×10^{-5}	-67.99 ± 14.17	101.26 ± 11.39	1.6×10^{-6}
CCI-onsite[a]	75	-101.95 (142.51)	121.33 (120.57)	5.7×10^{-10}	-105.15 ± 16.53	2.0×10^{-10}	-107.3 ± 15.56	122.16 ± 12.99	5.9×10^{-12}
Usual care[a]	25	41.25 (236.27)	240.85 (301.97)	0.38	36.25 ± 29.72	0.22	33.68 ± 27.85	234.53 ± 28.75	0.23
CCI-web vs. CCI-onsite[b]		25.77 ± 19.24		0.18	30.49 ± 24.79	0.22	39.31 ± 21.11		0.06
CCI-web vs. usual care[b]		-117.51 ± 48.27		0.01	-111.54 ± 34.93	0.002	-101.67 ± 31.25		0.001
CCI-onsite vs. usual care[b]		-143.28 ± 50.0		0.004	-142.03 ± 34.45	3.6×10^{-5}	-140.98 ± 31.95		1.0×10^{-5}
CCI-all vs. usual care[b]		-131.82 ± 48.34		0.006	-127.58 ± 32.43	8.4×10^{-5}	-122.09 ± 29.93		4.6×10^{-5}
**C-peptide (nmol·L^{-1})**									
CCI-all education[a]	185	-0.36 (0.57)	1.11 (0.59)	$<10^{-16}$	-0.34 ± 0.05	1.1×10^{-13}	-0.33 ± 0.04	1.11 ± 0.04	2.2×10^{-16}
CCI-web[a]	88	-0.4 (0.51)	1.0 (0.52)	3.8×10^{-13}	-0.39 ± 0.07	4.8×10^{-9}	-0.37 ± 0.06	1.02 ± 0.05	1.9×10^{-11}
CCI-onsite[a]	97	-0.32 (0.62)	1.21 (0.63)	3.6×10^{-7}	-0.3 ± 0.07	3.8×10^{-6}	-0.3 ± 0.06	1.19 ± 0.06	6.5×10^{-7}
Usual care[a]	59	0.08 (0.77)	1.43 (0.92)	0.41	0.02 ± 0.09	0.79	0.06 ± 0.09	1.44 ± 0.1	0.5
CCI-web vs. CCI-onsite[b]		-0.07 ± 0.08		0.37	-0.09 ± 0.09	0.36	-0.07 ± 0.08		0.36
CCI-web vs. usual care[b]		-0.48 ± 0.12		2.8×10^{-5}	-0.41 ± 0.11	0.0003	-0.43 ± 0.11		3.5×10^{-5}
CCI-onsite vs. usual care[b]		-0.4 ± 0.12		0.0007	-0.32 ± 0.11	0.005	-0.36 ± 0.11		0.0008
CCI-all vs. usual care[b]		-0.44 ± 0.11		5.4×10^{-5}	-0.37 ± 0.1	0.0004	-0.4 ± 0.1		5.3×10^{-5}

**HOMA-IR (insulin derived), all**									
CCI-all education[a]	179	-5.54 (12.19)	5.65 (8.71)	1.2x10^{-9}	-5.87 ± 0.92	2.2x10^{-10}	-5.58 ± 0.86	6.16 ± 0.69	7.5x10^{-11}
CCI-web[a]	84	-4.27 (11.9)	5.64 (10.27)	0.001	-4.32 ± 1.33	0.001	-4.35 ± 1.16	5.29 ± 0.99	0.0002
CCI-onsite[a]	95	-6.67 (12.39)	5.66 (7.11)	1.5x10^{-7}	-7.31 ± 1.28	1.3x10^{-8}	-6.72 ± 1.24	6.96 ± 0.95	6.3x10^{-8}
Usual care[a]	56	1.65 (12.46)	12.96 (12.9)	0.32	2.4 ± 1.76	0.17	1.82 ± 1.49	12.2 ± 1.42	0.22
CCI-web vs. CCI-onsite[b]		2.4 ± 1.82		0.19	2.99 ± 1.85	0.11	2.37 ± 1.7		0.16
CCI-web vs. usual care[b]		-5.92 ± 2.11		0.005	-6.86 ± 2.21	0.002	-6.17 ± 1.89		0.001
CCI-onsite vs. usual care[b]		-8.32 ± 2.09		7.1x10^{-5}	-9.85 ± 2.25	1.2x10^{-5}	-8.54 ± 1.94		1.1x10^{-5}
CCI-all vs. usual care[b]		-7.19 (1.9)		0.0002	-8.27±2.04	4.9x10^{-5}	-7.4 ± 1.72		1.6x10^{-5}
**HOMA-IR (insulin derived), excluding exogenous insulin users**									
CCI-all education[a]	129	-6.03 (10.67)	4.98 (5.69)	1.4x10^{-10}	-6.13 ± 0.98	4.2x10^{-10}	-6.82 ± 0.9	5.61 ± 0.51	3.2x10^{-5}
CCI-web[a]	57	-4.77 (6.0)	4.43 (3.19)	2.0x10^{-9}	-4.83 ± 1.48	0.001	-5.19 ± 1.1	4.56 ± 0.62	2.3x10^{-6}
CCI-onsite[a]	72	-7.02 (13.22)	5.4 (7.07)	6.6x10^{-6}	-7.17 ± 1.32	6.2x10^{-8}	-8.32 ± 1.37	6.59 ± 0.78	1.2x10^{-9}
Usual care[a]	25	3.99 (12.76)	13.35 (14.71)	0.12	4.1 ± 2.34	0.08	1.84 ± 1.96	13.3 ± 1.56	0.35
CCI-web vs. CCI-onsite[b]		2.25 ± 1.75		0.2	2.33 ± 2.0	0.25	3.13 ± 1.76		0.07
CCI-web vs. usual care[b]		-8.76 ± 2.67		0.001	-8.98 ± 2.77	0.002	-7.03 ± 2.25		0.002
CCI-onsite vs. usual care[b]		-11.01 ± 2.99		0.0002	-11.31 ± 2.72	3.2x10^{-5}	-10.16 ± 2.39		2.1x10^{-5}
CCI-all vs. usual care[b]		-10.01 ± 2.72		0.0002	-10.23 ± 2.56	6.3x10^{-5}	-8.65 ± 2.16		6.0x10^{-5}
**HOMA-IR (C-peptide derived)**									
CCI-all education[a]	170	-3.53 (5.59)	7.9 (3.89)	2.2x10^{-16}	-3.53 ± 0.55	1.2x10^{-10}	-3.45 ± 0.46	8.25 ± 0.4	1.0x10^{-13}
CCI-web[a]	79	-3.45 (5.36)	7.29 (3.62)	1.0x10^{-8}	-3.52 ± 0.79	9.2x10^{-6}	-3.55 ± 0.61	7.38 ± 0.5	7.6x10^{-9}
CCI-onsite[a]	91	-3.61 (5.81)	8.43 (4.05)	3.1x10^{-9}	-3.53 ± 0.77	3.9x10^{-6}	-3.36 ± 0.69	9.06 ± 0.6	1.2x10^{-6}
Usual care[a]	47	1.94 (10.54)	12.49 (10.46)	0.21	1.77 ± 1.12	0.11	1.65 ± 1.13	12.6 ± 1.11	0.14
CCI-web vs. CCI-onsite[b]		0.15 ± 0.86		0.86	0.01 ± 1.11	0.99	-0.19 ± 0.93		0.84
CCI-web vs. usual care[b]		-5.39 ± 1.65		0.001	-5.29 ± 1.38	0.0002	-5.2 ± 1.29		5.3x10^{-5}
CCI-onsite vs. usual care[b]		-5.55 ± 1.65		0.0008	-5.3 ± 1.42	0.0002	-5.02 ± 1.33		0.0002
CCI-all vs. usual care[b]		-5.47 (1.6)		0.0006	-5.29 ± 1.28	3.3 x10^{-5}	-5.11 ± 1.22		3.0x10^{-5}
**Weight-clinic (kg)**									
CCI-all education[a]	184	-14.24 (10.29)	101.17 (22.06)	$<10^{-16}$	-13.81 ± 0.63	$<10^{-16}$	-13.8 ± 0.71	102.72 ± 1.5	$<10^{-16}$
CCI-web[a]	84	-13.77 (9.84)	101.0 (23.29)	$<10^{-16}$	-13.93 ± 0.92	$<10^{-16}$	-13.7 ± 1.01	100.98 ± 2.25	$<10^{-16}$
CCI-onsite[a]	100	-14.64 (10.69)	101.31 (21.09)	$<10^{-16}$	-13.71 ± 0.86	$<10^{-16}$	-13.89 ± 0.99	104.33 ± 1.99	$<10^{-16}$
Usual care[a]	69	0.04 (5.94)	106.82 (22.52)	0.95	-1.11 ± 1.06	0.29	-0.16 ± 0.84	107.31 ± 2.55	0.85
CCI-web vs. CCI-onsite[b]		0.87 ± 1.51		0.57	-0.22 ± 1.26	0.86	0.19 ± 1.41		0.89
CCI-web vs. usual care[b]		-13.81 ± 1.29		$<10^{-16}$	-12.81 ± 1.42	$<10^{-16}$	-13.55 ± 1.32		$<10^{-16}$
CCI-onsite vs. usual care[b]		-14.68 ± 1.29		$<10^{-16}$	-12.59 ± 1.4	$<10^{-16}$	-13.74 ± 1.3		$<10^{-16}$
CCI-all vs. usual care[b]		-14.29 ± 1.04		$<10^{-16}$	-12.7 ± 1.26	$<10^{-16}$	-13.65 ± 1.1		$<10^{-16}$

**Systolic blood pressure (mmHg)**									
CCI-all education[a]	187	-6.77 (16.3)	125.84 (13.22)	1.3x10^{-8}	-6.52 ± 1.24	1.6x10^{-7}	-6.36 ± 1.12	125.57 ± 0.91	1.3x10^{-8}
CCI-web[a]	87	-7.38 (16.39)	125.66 (10.72)	2.7x10^{-5}	-7.31 ± 1.81	5.2x10^{-5}	-6.91 ± 1.63	125.72 ± 1.39	2.3x10^{-5}
CCI-onsite[a]	100	-6.24 (16.28)	128.97 (15.6)	0.0001	-5.8 ± 1.72	0.0007	-5.85 ± 1.53	125.43 ± 1.18	0.0001
Usual care[a]	67	0.25 (17.8)	128.57 (11.82)	0.91	-0.45 ± 2.15	0.83	-0.9 ± 2.07	129.01 ± 1.72	0.67
CCI-web vs. CCI-onsite[b]		-1.14 ± 2.4		0.63	-1.51 ± 2.49	0.55	-1.06 ± 2.24		0.64
CCI-web vs. usual care[b]		-7.63 ± 2.8		0.006	-6.83 ± 2.84	0.02	-6.01 ± 2.64		0.02
CCI-onsite vs. usual care[b]		-6.49 ± 2.72		0.02	-5.32 ± 2.84	0.06	-4.95 ± 2.58		0.05
CCI-all vs. usual care[b]		-7.02 (2.4)		0.005	-6.07 ± 2.55	0.02	-5.46 ± 2.36		0.02
**Diastolic blood pressure (mmHg)**									
CCI-all education[a]	187	-3.59 (9.33)	78.0 (7.55)	1.4x10^{-7}	-3.5 ± 0.7	6.2x10^{-7}	-3.51 ± 0.65	78.58 ± 0.56	7.2x10^{-8}
CCI-web[a]	87	-3.36 (9.94)	78.16 (7.66)	0.002	-3.3 ± 1.02	0.001	-3.77 ± 1.0	78.83 ± 0.83	0.0002
CCI-onsite[a]	100	-3.8 (8.81)	77.86 (7.5)	1.6x10^{-5}	-3.67 ± 0.97	0.0001	-3.28 ± 0.85	78.35 ± 0.75	0.0001
Usual care[a]	67	-0.12 (10.15)	80.99 (9.59)	0.92	-0.39 ± 1.21	0.75	-0.9 ± 1.2	81.12 ± 1.08	0.45
CCI-web vs. CCI-onsite[b]		0.44 ± 1.38		0.75	0.37 ± 1.4	0.79	-0.49 ± 1.31		0.71
CCI-web vs. usual care[b]		-3.24 ± 1.63		0.05	-2.92 ± 1.6	0.07	-2.87 ± 1.56		0.07
CCI-onsite vs. usual care[b]		-3.68 ± 1.52		0.02	-3.29 ± 1.6	0.04	-2.38 ± 1.47		0.11
CCI-all vs. usual care[b]		-3.47 ± 1.42		0.01	-3.10±1.44	0.03	-2.61 ± 1.37		0.06
**Total cholesterol (mmol·L^{-1})**									
CCI-all education[a]	186	0.24 (0.93)	4.92 (1.18)	0.0004	0.24 ± 0.08	0.004	0.21 ± 0.07	4.97 ± 0.08	0.006
CCI-web[a]	88	0.25(0.93)	4.83 (1.19)	0.01	0.26± 0.12	0.04	0.16 ± 0.11	4.95 ± 0.12	0.15
CCI-onsite[a]	98	0.24 (0.94)	5.0 (1.17)	0.01	0.23 ± 0.12	0.05	0.25 ± 0.1	4.99 ± 0.12	0.02
Usual care[a]	59	0.00 (1.60)	4.72 (1.62)	0.99	0.0 ± 0.16	0.98	-0.04 ± 0.18	4.69 ± 0.18	0.83
CCI-web vs. CCI-onsite[b]		0.01 ± 0.14		0.97	0.02 ± 0.17	0.89	-0.09 ± 0.15		0.54
CCI-web vs. usual care[b]		0.25 ± 0.23		0.28	0.26 ± 0.20	0.19	0.2 ± 0.21		0.34
CCI-onsite vs. usual care[b]		0.24 ± 0.23		0.29	0.24 ± 0.20	0.25	0.29 ± 0.2		0.16
CCI-all vs. usual care[b]		0.25 ± 0.22		0.26	0.25 ± 0.18	0.17	0.24 ± 0.19		0.2
**LDL-C (mmol·L^{-1})**									
CCI-all education[a]	172	0.28 (0.83)	2.87 (0.98)	7.7x10^{-6}	0.28 ± 0.07	2.6x10^{-5}	0.26 ± 0.06	2.94 ± 0.07	5.1x10^{-5}
CCI-web[a]	83	0.30 (0.86)	2.82 (1.01)	0.002	0.31 ± 0.10	0.001	0.22 ± 0.09	2.91 ± 0.1	0.02
CCI-onsite[a]	89	0.27(0.80)	2.93 (0.95)	0.002	0.25 ± 0.09	0.007	0.29 ± 0.09	2.97 ± 0.1	0.0009
Usual care[a]	48	-0.28 (0.97)	2.32 (0.8)	0.05	-0.28 ± 0.13	0.03	-0.28 ± 0.12	2.32 ± 0.12	0.02
CCI-web vs. CCI-onsite[b]		0.03± 0.13		0.81	0.06 ± 0.14	0.67	-0.07 ± 0.13		0.59
CCI-web vs. usual care[b]		0.58 ± 0.17		0.0006	0.59± 0.16	0.0004	0.51 ± 0.15		0.001
CCI-onsite vs. usual care[b]		0.55 ± 0.16		0.0009	0.53 ± 0.17	0.002	0.58 ± 0.15		0.0001

CCI-all vs. usual care[b]		0.56 ± 0.15		0.0003	0.56 ± 0.15	0.0002	0.54 ± 0.14		0.0001
**Apo B (g·L^{-1})**									
CCI-all education[a]	186	-0.01 (0.24)	1.03 (0.29)	0.69	-0.0 ± 0.02	0.82	-0.02 ± 0.02	1.04 ± 0.02	0.37
CCI-web[a]	88	-0.0 (0.2)	1.03 (0.29)	0.95	-0.0 ± 0.03	0.93	-0.03 ± 0.03	1.05 ± 0.03	0.28
CCI-onsite[a]	98	-0.01 (0.27)	1.03 (0.3)	0.66	-0.01 ± 0.03	0.82	-0.01 ± 0.03	1.02 ± 0.03	0.82
Usual care[a]	59	0.02 (0.37)	1.07 (0.39)	0.75	0.0 ± 0.04	0.9	0.0 ± 0.04	1.06 ± 0.04	0.95
CCI-web vs. CCI-onsite[b]		0.01 ± 0.03		0.76	0.0 ± 0.04	0.92	-0.02 ± 0.04		0.56
CCI-web vs. usual care[b]		-0.02 ± 0.05		0.75	-0.01 ± 0.05	0.87	-0.03 ± 0.05		0.53
CCI-onsite vs. usual care[b]		-0.03 ± 0.06		0.62	-0.01 ± 0.05	0.81	-0.01 ± 0.05		0.86
CCI-all vs. usual care[b]		-0.02 (0.05)		0.66	-0.01 ± 0.05	0.83	-0.02 ± 0.05		0.67
**HDL-C (mmol·L^{-1})**									
CCI-all education[a]	186	0.20 (0.31)	1.29 (0.41)	$<10^{-16}$	0.19 ± 0.02	$<10^{-16}$	0.2 ± 0.02	1.29 ± 0.03	$<10^{-16}$
CCI-web[a]	88	0.16 (0.33)	1.26 (0.42)	$2.9x10^{-6}$	0.17 ± 0.03	$7.3x10^{-8}$	0.18 ± 0.03	1.28 ± 0.04	$2.9x10^{-8}$
CCI-onsite[a]	98	0.23 (0.29)	1.32 (0.41)	$7.1x10^{-11}$	0.21 ± 0.03	$5.3x10^{-12}$	0.22 ± 0.03	1.31 ± 0.04	$2.3x10^{-14}$
Usual care[a]	59	-0.04 (0.23)	0.92 (0.32)	0.15	-0.02 ± 0.04	0.69	-0.03 ± 0.03	0.95 ± 0.04	0.41
CCI-web vs. CCI-onsite[b]		-0.06 ± 0.05		0.18	-0.04 ± 0.04	0.36	-0.04 ± 0.04		0.31
CCI-web vs. usual care[b]		0.21 ± 0.05		$7.1x10^{-6}$	0.18 ± 0.05	0.0003	0.2 ± 0.05		$1.0x10^{-5}$
CCI-onsite vs. usual care[b]		0.27 ± 0.04		$1.1x10^{-10}$	0.22 ± 0.05	$1.3x10^{-5}$	0.25 ± 0.04		$2.3x10^{-8}$
CCI-all vs. usual care[b]		0.24 ± 0.04		$1.7x10^{-10}$	0.2 ± 0.05	$9.9x10^{-6}$	0.23 ± 0.04		$1.3x10^{-8}$
**Triglycerides (mmol·L^{-1})**									
CCI-all education[a]	186	-0.56 (1.9)	1.71 (1.64)	$<10^{-16}$	-0.56 ± 0.18	$9.3x10^{-15}$	-0.54 ± 0.14	1.67 ± 0.13	$<10^{-16}$
CCI-web[a]	88	-0.36 (1.79)	1.8 (1.77)	$2.2x10^{-6}$	-0.34 ± 0.25	$2.0x10^{-7}$	-0.4 ± 0.2	1.73 ± 0.19	$1.3x10^{-7}$
CCI-onsite[a]	98	-0.74(1.97)	1.62 (1.51)	$3.4x10^{-14}$	-0.78 ± 0.25	$6.0x10^{-13}$	-0.67 ± 0.21	1.62 ± 0.18	$1.9x10^{-12}$
Usual care[a]	59	0.34 (3.40)	3.7 (5.67)	0.22	-0.35 ± 0.32	0.48	0.32 ± 0.37	3.45 ± 0.55	0.43
CCI-web vs. CCI-onsite[b]		0.38 ± 0.28		0.16	0.44 ± 0.35	0.20	0.28 ± 0.29		0.34
CCI-web vs. usual care[b]		-0.7 ± 0.48		$6.9x10^{-5}$	-0.71 ± 0.41	0.0004	-0.71 ± 0.42		$8.7x10^{-5}$
CCI-onsite vs. usual care[b]		-1.08 ± 0.48		$4.8x10^{-8}$	1.15 ± 0.42	$4.2x10^{-6}$	-0.99 ± 0.42		$1.2x10^{-6}$
CCI-all vs. usual care[b]		-0.9 ± 0.46		$1.4x10^{-7}$	-0.92 ± 0.38	$7.5x10^{-6}$	-0.86 ± 0.39		$9.9x10^{-7}$
**Total/HDL-cholesterol**									
CCI-all education[a]	186	-0.47 (1.41)	4.18 (1.71)	$4.1 x10^{-6}$	-0.45 ± 0.16	0.005	-0.53 ± 0.12	4.19 ± 0.13	$1.7x10^{-5}$
CCI-web[a]	88	-0.37 (1.41)	4.19 (1.57)	0.02	-0.35 ± 0.22	0.11	-0.47 ± 0.18	4.2 ± 0.18	0.007
CCI-onsite[a]	98	-0.57 (1.4)	4.17 (1.84)	$5.1 x10^{-5}$	-0.54 ± 0.22	0.01	-0.58 ± 0.17	4.17 ± 0.2	0.0008
Usual care[a]	59	0.52 (3.45)	5.96 (4.27)	0.24	0.44 ± 0.29	0.13	0.42 ± 0.36	5.73 ± 0.43	0.24
CCI-web vs. CCI-onsite[b]		0.21 ± 0.21		0.31	0.19 ± 0.31	0.61	0.1 ± 0.25		0.67
CCI-web vs. usual care[b]		-0.89 ± 0.47		0.06	-0.80 ± 0.36	0.03	-0.89 ± 0.4		0.03

CCI-onsite vs. usual care[b]		-1.09 ± 0.47		0.02	-1.00 ± 0.37	0.008	-1.0 ± 0.4		0.01
CCI-all vs. usual care[b]		-1.00 ± 0.46		0.03	-0.89 ± 0.33	0.008	-0.95 ± 0.38		0.01
**hsC-reactive protein (nmol·L^{-1})**									
CCI-all education[a]	193	-31.71 (127.15)	53.81 (67.24)	$<10^{-8}$	-29.43 ± 9.14	$<10^{-16}$	-34.29 ± 10.0	52.86 ± 5.24	$<10^{-16}$
CCI-web[a]	98	-42.38 (167.34)	56.0 (68.67)	$5.9x10^{-11}$	-40.1 ± 12.76	$1.5x10^{-10}$	-34.86 ± 14.29	54.57 ± 7.62	$6.1x10^{-11}$
CCI-onsite[a]	95	-20.76 (62.1)	51.72 (66.0)	$3.1x10^{-11}$	-18.19 ± 13.14	$4.7x10^{-12}$	-33.81 ± 13.91	51.24 ± 7.24	$7.8x10^{-13}$
Usual care[a]	70	12.48 (126.86)	99.43 (139.91)	0.94	8.48 ± 16.1	0.88	12.48 ± 14.1	97.72 ± 14.76	0.93
CCI-web vs. CCI-onsite[b]		-21.62 ± 18.1		0.44	-21.91 ± 18.29	0.62	-1.05 ± 20.0		0.43
CCI-web vs. usual care[b]		-54.86 ± 22.67		$4.7x10^{-5}$	-48.1 ± 20.67	$3.5x10^{-5}$	-47.33 ± 20.1		$4.9x10^{-5}$
CCI-onsite vs. usual care[b]		-33.24 ± 16.48		$6.7x10^{-6}$	-26.29 ± 21.24	$8.2x10^{-6}$	-46.29 ± 19.81		$3.4x10^{-6}$
CCI-all vs. usual care[b]		-44.29 (17.14)		$1.2x10^{-6}$	-37.91 ± 18.55	$3.0x10^{-5}$	-46.76 ± 17.24		$9.3x10^{-7}$
**ALT (µkat·L^{-1})**									
CCI-all education[a]	201	-0.16 (0.4)	0.36 (0.19)	$9.5x10^{-9}$	-0.16 ± 0.03	$9.4x10^{-10}$	-0.15 ± 0.02	0.36 ± 0.01	$2.4x10^{-10}$
CCI-web[a]	97	-0.18 (0.31)	0.37 (0.15)	$4.9x10^{-9}$	-0.19 ± 0.04	$1.3x10^{-6}$	-0.17 ± 0.03	0.37 ± 0.02	$4.1x10^{-9}$
CCI-onsite[a]	104	-0.14 (0.47)	0.35 (0.22)	0.002	-0.14 ± 0.04	0.0001	-0.14 ± 0.04	0.36 ± 0.02	0.0004
Usual care[a]	71	0.01 (0.28)	0.47 (0.34)	0.67	0.02 ± 0.05	0.67	0.01 ± 0.03	0.47 ± 0.04	0.77
CCI-web vs. CCI-onsite[b]		-0.04 ± 0.06		0.48	-0.04 ± 0.39	0.43	-0.04 ± 0.05		0.46
CCI-web vs. usual care[b]		-0.2 ± 0.05		$1.5x10^{-5}$	-0.2 ± 0.06	0.0009	-0.18 ± 0.04		$2.7x10^{-5}$
CCI-onsite vs. usual care[b]		-0.16 ± 0.06		0.006	-0.16 ± 0.06	0.009	-0.15 ± 0.05		0.003
CCI-all vs. usual care[b]		-0.18 (0.04)		$5.1x10^{-5}$	-0.18 ± 0.05	0.0009	-0.16 ± 0.04		$4.6x10^{-5}$
**AST (µkat·L^{-1})**									
CCI-all education[a]	201	-0.09 (0.27)	0.32 (0.11)	$2.8x10^{-6}$	-0.09 ± 0.02	$1.3x10^{-5}$	-0.08 ± 0.02	0.32 ± 0.01	$5.1x10^{-7}$
CCI-web[a]	97	-0.12 (0.29)	0.32 (0.1)	$5.1x10^{-5}$	-0.12 ± 0.03	$2.4x10^{-5}$	-0.11 ± 0.03	0.32 ± 0.01	$2.0x10^{-5}$
CCI-onsite[a]	104	-0.06 (0.24)	0.31 (0.12)	0.01	-0.06 ± 0.03	0.05	-0.06 ± 0.02	0.31 ± 0.01	0.005
Usual care[a]	71	0.01 (0.32)	0.4 (0.27)	0.79	0.01 ± 0.04	0.69	0.01 ± 0.03	0.41 ± 0.03	0.72
CCI-web vs. CCI-onsite[b]		-0.06 ± 0.04		0.12	-0.07 ± 0.04	0.11	-0.05 ± 0.03		0.12
CCI-web vs. usual care[b]		-0.13 ± 0.05		0.007	-0.13 ± 0.05	0.004	-0.12 ± 0.04		0.004
CCI-onsite vs. usual care[b]		-0.07 ± 0.04		0.12	-0.07 ± 0.05	0.14	-0.07 ± 0.04		0.08
CCI-all vs. usual care[b]		-0.1 (0.04)		0.02	-0.1 ± 0.04	0.02	-0.09 ± 0.04		0.01
**Alkaline phosphatase (µkat·L^{-1})**									
CCI-all education[a]	200	-0.16 (0.24)	1.07 (0.35)	$<10^{-8}$	-0.17 ± 0.02	$<10^{-16}$	-0.16 ± 0.02	1.08 ± 0.02	$<10^{-16}$
CCI-web[a]	97	-0.14 (0.21)	1.09 (0.37)	$4.8x10^{-11}$	-0.15 ± 0.02	$9.3 x10^{-10}$	-0.14 ± 0.02	1.08 ± 0.03	$3.3x10^{-11}$
CCI-onsite[a]	103	-0.19 (0.26)	1.06 (0.34)	$4.2x10^{-13}$	-0.19 ± 0.02	$1.8x10^{-15}$	-0.18 ± 0.02	1.08 ± 0.03	$2.5x10^{-13}$
Usual care[a]	71	0.0 (0.22)	1.31 (0.45)	0.94	0.02 ± 0.03	0.61	0.01 ± 0.03	1.3 ± 0.05	0.67
CCI-web vs. CCI-onsite[b]		0.05 ± 0.03		0.17	0.04 ± 0.03	0.22	0.03 ± 0.03		0.32

CCI-web vs. usual care[b]		-0.14 ± 0.03		2.0x10^{-5}	-0.16 ± 0.04	2.1x10^{-5}	-0.15 ± 0.03		5.4x10^{-6}
CCI-onsite vs. usual care[b]		-0.19 ± 0.04		2.2x10^{-7}	-0.2 ± 0.04	1.5x10^{-7}	-0.19 ± 0.04		1.6x10^{-7}
CCI-all vs. usual care[b]		-0.17 (0.03)		6.3x10^{-8}	-0.18 ± 0.03	1.4x10^{-7}	-0.17 ± 0.03		3.1x10^{-8}
**Serum creatinine (µmol·L^{-1})**									
CCI-all education[a]	202	-3.54 (14.14)	73.37 (18.56)	0.0001	-3.54 ± 0.88	0.001	-3.54 ± 0.88	74.26 ± 0.88	0.0001
CCI-web[a]	97	-4.42 (10.61)	73.37 (17.68)	0.0003	-3.54 ± 1.77	0.01	-3.54 ± 0.88	73.37 ± 1.77	0.001
CCI-onsite[a]	105	-3.54 (15.91)	74.26 (20.33)	0.03	-3.54 ± 1.77	0.04	-3.54 ± 1.77	76.02 ± 1.77	0.01
Usual care[a]	71	-0.88 (18.56)	77.79 (18.56)	0.56	-2.65 ± 1.77	0.15	-1.77 ± 1.77	78.68 ± 1.77	0.29
CCI-web vs. CCI-onsite[b]		-0.88 ± 1.77		0.76	-0.88 ± 2.65	0.76	0.0 ± 1.77		0.96
CCI-web vs. usual care[b]		-2.65 ± 2.65		0.27	-0.88 ± 2.65	0.66	-1.77 ± 2.65		0.49
CCI-onsite vs. usual care[b]		-1.77 ± 2.65		0.43	0.0 ± 2.65	0.86	-1.77 ± 2.65		0.55
CCI-all vs. usual care[b]		-2.65 ± 2.65		0.32	-0.88 ± 2.65	0.73	-1.77 ± 2.65		0.49
**BUN (mmol·L^{-1})**									
CCI-all education[a]	202	0.76 (2.49)	6.82 (2.78)	1.5x10^{-5}	0.75 ± 0.17	1.6x10^{-5}	0.79 ± 0.17	6.81 ± 0.19	5.5x10^{-6}
CCI-web[a]	97	0.45 (1.73)	6.62 (2.18)	0.01	0.46 ± 0.25	0.07	0.5 ± 0.2	6.51 ± 0.23	0.01
CCI-onsite[a]	105	1.04 (3.01)	7.0 (3.25)	0.0004	1.04 ± 0.24	2.0x10^{-5}	1.06 ± 0.28	7.08 ± 0.3	0.0001
Usual care[a]	71	0.07 (2.15)	5.66 (2.11)	0.78	0.06 ± 0.3	0.85	-0.01 ± 0.27	5.72 ± 0.26	0.97
CCI-web vs. CCI-onsite[b]		-0.6 ± 0.34		0.08	-0.58 ± 0.35	0.1	-0.56 ± 0.34		0.1
CCI-web vs. usual care[b]		0.38 ± 0.31		0.22	0.41 ± 0.39	0.29	0.51 ± 0.34		0.13
CCI-onsite vs. usual care[b]		0.97 ± 0.39		0.01	0.99 ± 0.4	0.01	1.07 ± 0.39		0.005
CCI-all vs. usual care[b]		0.69 (0.29)		0.03	0.69 (0.32)	0.05	0.8 ± 0.32		0.01
**eGFR (mL·s^{-1}·m^{-2})**									
CCI-all education[a]	202	0.03 (0.15)	1.38 (0.2)	0.003	0.03 ± 0.01	0.009	0.03 ± 0.01	1.38 ± 0.01	0.005
CCI-web[a]	97	0.03 (0.14)	1.4 (0.17)	0.01	0.03 ± 0.02	0.04	0.03 ± 0.01	1.4 ± 0.02	0.03
CCI-onsite[a]	105	0.03 (0.17)	1.36 (0.23)	0.08	0.03 ± 0.02	0.1	0.03 ± 0.02	1.35 ± 0.02	0.07
Usual care[a]	71	0.01 (0.19)	1.34 (0.22)	0.51	0.02 ± 0.02	0.36	0.01 ± 0.02	1.34 ± 0.02	0.48
CCI-web vs. CCI-onsite[b]		0.01 ± 0.02		0.79	0.01 ± 0.02	0.77	0.0 ± 0.02		0.89
CCI-web vs. usual care[b]		0.02 ± 0.03		0.42	0.02 ± 0.03	0.56	0.02 ± 0.03		0.51
CCI-onsite vs. usual care[b]		0.01 ± 0.03		0.58	0.01 ± 0.03	0.75	0.01 ± 0.03		0.6
CCI-all vs. usual care[b]		0.03 ± 0.02		0.28	0.01 ± 0.02	0.61	0.02 ± 0.02		0.51
**Anion gap (mmol·L^{-1})**									
CCI-all education[a]	201	0.29 (2.02)	7.08 (1.75)	0.04	0.28 ± 0.15	0.06	0.28 ± 0.14	7.13 ± 0.12	0.04
CCI-web[a]	97	0.07 (1.97)	7.04 (1.77)	0.72	0.11 ± 0.22	0.61	0.12 ± 0.2	7.05 ± 0.18	0.55
CCI-onsite[a]	104	0.49 (2.06)	7.12 (1.74)	0.02	0.45 ± 0.21	0.1	0.43 ± 0.19	7.19 ± 0.17	0.02
Usual care[a]	71	0.83 (2.44)	7.75 (1.97)	0.004	0.84 ± 0.26	0.001	0.81 ± 0.26	7.75 ± 0.22	0.002

CCI-web vs. CCI-onsite[b]		-0.42 ± 0.28		0.14	-0.35 ± 0.3	0.26	-0.31 ± 0.28		0.26
CCI-web vs. usual care[b]		-0.76 ± 0.35		0.03	-0.72 ± 0.34	0.04	-0.69 ± 0.33		0.04
CCI-onsite vs. usual care[b]		-0.34 ± 0.35		0.33	-0.38 ± 0.35	0.28	-0.38 ± 0.33		0.25
CCI-all vs. usual care[b]		-0.54 (0.3)		0.09	-0.56 ± 0.31	0.07	-0.53 ± 0.3		0.08
**CO_2 (mmol·L^{-1})**									
CCI-all education[a]	202	0.12 (2.26)	28.06 (2.29)	0.45	0.16 ± 0.18	0.38	0.18 ± 0.16	27.94 ± 0.16	0.27
CCI-web[a]	97	0.44 (2.02)	28.41 (2.12)	0.03	0.41 ± 0.25	0.11	0.45 ± 0.22	28.34 ± 0.22	0.04
CCI-onsite[a]	105	-0.18 (2.43)	27.74 (2.41)	0.45	-0.08 ± 0.25	0.73	-0.07 ± 0.24	27.57 ± 0.23	0.77
Usual care[a]	71	0.17 (2.97)	28.23 (2.58)	0.63	-0.2 ± 0.3	0.51	0.04 ± 0.33	28.12 ± 0.29	0.9
CCI-web vs. CCI-onsite[b]		0.62 ± 0.31		0.05	0.49 ± 0.35	0.16	0.52 ± 0.32		0.11
CCI-web vs. usual care[b]		0.27 ± 0.41		0.5	0.6 ± 0.4	0.13	0.41 ± 0.4		0.3
CCI-onsite vs. usual care[b]		-0.35 ± 0.42		0.41	0.11 ± 0.4	0.79	-0.11 ± 0.41		0.78
CCI-all vs. usual care[b]		-0.05 (0.3)		0.9	0.36 ± 0.36	0.32	0.14 ± 0.37		0.71
**Uric acid (µmol·L^{-1})**									
CCI-all education[a]	202	0.59 (70.79)	349.18 (91.01)	0.91	1.78 ± 4.76	0.72	1.78 ± 4.76	349.77 ± 5.95	0.67
CCI-web[a]	98	-0.59 (77.93)	360.48 (83.87)	0.93	0.59 ± 7.14	0.95	1.78 ± 7.14	356.91 ± 8.33	0.8
CCI-onsite[a]	104	1.78 (63.65)	338.47 (95.77)	0.77	2.97 ± 7.14	0.65	2.38 ± 6.54	343.23 ± 8.92	0.72
Usual care[a]	71	-10.71 (64.24)	320.03 (85.06)	0.15	-16.66 ± 8.92	0.06	-11.9 ± 7.73	322.41 ± 10.11	0.13
CCI-web vs. CCI-onsite[b]		-2.38 ± 10.11		0.8	-2.97 ± 10.11	0.79	-0.59 ± 9.52		0.96
CCI-web vs. usual care[b]		10.11 ± 10.71		0.35	17.25 ± 11.3	0.13	13.68 ± 10.71		0.21
CCI-onsite vs. usual care[b]		12.49 ± 10.11		0.2	20.22 ± 11.3	0.08	14.28 ± 10.11		0.16
CCI-all vs. usual care[b]		11.3 ± 8.92		0.21	18.44 ± 10.11	0.07	13.68 ± 8.92		0.13
**TSH (mIU·L^{-1})**									
CCI-all education[a]	200	-0.41 (1.62)	1.9 (1.1)	0.0004	-0.4 ± 0.11	0.0002	-0.42 ± 0.1	1.91 ± 0.08	$5.3x10^{-5}$
CCI-web[a]	98	-0.44 (1.83)	2.02 (1.19)	0.02	-0.45 ± 0.15	0.003	-0.39 ± 0.16	1.99 ± 0.11	0.01
CCI-onsite[a]	102	-0.38 (1.4)	1.78 (0.99)	0.006	-0.35 ± 0.15	0.02	-0.45 ± 0.14	1.83 ± 0.1	0.001
Usual care[a]	70	-0.09 (0.99)	2.01 (0.99)	0.47	-0.09 ± 0.19	0.64	0.0 ± 0.12	1.96 ± 0.12	0.98
CCI-web vs. CCI-onsite[b]		-0.06 ± 0.23		0.8	-0.1 ± 0.21	0.64	0.06 ± 0.21		0.77
CCI-web vs. usual care[b]		-0.35 ± 0.22		0.11	-0.36 ± 0.24	0.14	-0.38 ± 0.2		0.05
CCI-onsite vs. usual care[b]		-0.3 ± 0.18		0.1	-0.26 ± 0.25	0.29	-0.45 ± 0.18		0.02
CCI-all vs. usual care[b]		-0.33 ± 0.16		0.05	-0.31 ± 0.22	0.15	-0.42 ± 0.16		0.01
**Free T4 (pmol·L^{-1})**									
CCI-all education[a]	202	0.13 (2.32)	11.97 (2.45)	0.7	0.0 ± 0.26	0.83	0.13 ± 0.13	11.97 ± 0.13	0.58
CCI-web[a]	98	0.26 (2.32)	11.97 (2.06)	0.33	0.26 ± 0.26	0.44	0.13 ± 0.26	11.97 ± 0.26	0.51
CCI-onsite[a]	104	-0.13 (2.45)	11.84 (2.7)	0.72	-0.13 ± 0.26	0.64	0.0 ± 0.26	11.84 ± 0.26	0.88

Usual care[a]	71	0.26 (4.25)	11.58 (2.83)	0.7	0.26 ± 0.39	0.48	0.26 ± 0.39	11.46 ± 0.26	0.61
CCI-web vs. CCI-onsite[b]		0.26 ± 0.39		0.35	0.39 ± 0.39	0.38	0.13 ± 0.39		0.72
CCI-web vs. usual care[b]		0.0 ± 0.51		0.97	-0.13 ± 0.51	0.89	-0.13 ± 0.51		0.89
CCI-onsite vs. usual care[b]		-0.26 ± 0.51		0.61	-0.39 ± 0.51	0.37	-0.13 ± 0.51		0.71
CCI-all vs. usual care[b]		-0.13 (0.0)		0.8	-0.26 ± 0.39	0.43	-0.13 ± 0.51		0.78
**Any diabetes medication, excluding metformin (%)**									
CCI-all education[a]	218	-27.52 (49.65)	27.98 ± 3.05	2.2×10^{-16}	-27.66 ± 3.21	$<10^{-16}$	-27.19 ± 3.14	29.68 ± 2.94	$<10^{-16}$
CCI-web[a]	104	-31.73 (50.75)	25.0 ± 4.27	1.8×10^{-10}	-31.64 ± 4.62	7.5×10^{-12}	-30.56 ± 4.6	26.58 ± 4.13	4.1×10^{-11}
CCI-onsite[a]	114	-23.68 (48.52)	30.7 ± 4.34	1.9×10^{-7}	-23.95 ± 4.47	8.4×10^{-8}	-24.08 ± 4.31	32.54 ± 4.19	2.4×10^{-8}
Usual care[a]	78	6.85 (34.68)	75.34 ± 5.08	0.09	7.54 ± 5.87	0.2	5.99 ± 4.31	72.66 ± 5.0	0.09
CCI-web vs. CCI-onsite[b]		-8.05 ± 6.74		0.23	-7.69 ± 6.42	0.23	-6.48 ± 6.3		0.3
CCI-web vs. usual care[b]		-38.58 ± 6.42		1.9×10^{-9}	-39.18 ± 7.53	2.0×10^{-7}	-36.55 ± 6.31		1.9×10^{-9}
CCI-onsite vs. usual care[b]		-30.53 ± 6.09		5.4×10^{-7}	-31.49 ± 7.55	3.0×10^{-5}	-30.07 ± 6.1		2.4×10^{-7}
CCI-all vs. usual care[b]		-34.37 ± 5.27		7.0×10^{-11}	-35.36 ± 6.83	2.3×10^{-7}	-33.19 ± 5.34		9.0×10^{-9}
**Sulfonylurea (%)**									
CCI-all education[a]	218	-24.31 (43.0)	0.0 ± 0.0	$<10^{-16}$	-24.23 ± 2.86	$<10^{-16}$	-23.67 ± 2.7	0.0 ± 0.0	$<10^{-16}$
CCI-web[a]	104	-25.96 (44.05)	0.0 ± 0.0	1.9×10^{-9}	-26.04 ± 4.12	2.6×10^{-10}	-24.09 ± 3.92	0.0 ± 0.0	8.3×10^{-10}
CCI-onsite[a]	114	-22.81 (42.14)	0.0 ± 0.0	7.6×10^{-9}	-22.53 ± 3.98	1.5×10^{-8}	-23.29 ± 3.72	0.0 ± 0.0	3.9×10^{-10}
Usual care[a]	78	2.74 (37.17)	26.02 ± 5.17	0.53	2.56 ± 5.24	0.63	1.91 ± 4.23	26.02 ± 5.17	0.65
CCI-web vs. CCI-onsite[b]		-3.15 ± 5.85		0.59	-3.52 ± 5.72	0.54	-0.8 ± 5.41		0.88
CCI-web vs. usual care[b]		-28.7 ± 6.13		2.9×10^{-6}	-28.6 ± 6.71	2.0×10^{-5}	-26.0 ± 5.77		6.7×10^{-5}
CCI-onsite vs. usual care[b]		-25.55 ± 5.87		1.4×10^{-5}	-25.08 ± 6.73	0.0002	-25.2 ± 5.64		7.8×10^{-6}
CCI-all vs. usual care[b]		-27.05 ± 5.23		2.4×10^{-7}	-26.85 ± 6.07	9.7×10^{-6}	-25.58 ± 5.02		3.3×10^{-7}
**Insulin (%)**									
CCI-all education[a]	218	-13.3 (35.37)	15.14 ± 2.43	2.8×10^{-8}	-15.5 ± 2.0	9.3×10^{-15}	-13.03 ± 2.22	16.74 ± 2.4	4.3×10^{-9}
CCI-web[a]	104	-11.54 (35.0)	16.35 ± 3.64	0.0008	-13.71 ± 2.87	1.8×10^{-6}	-11.47 ± 3.19	17.01 ± 3.52	0.0003
CCI-onsite[a]	114	-14.91 (35.78)	14.04 ± 3.27	8.6×10^{-6}	-17.18 ± 2.78	6.4×10^{-10}	-14.47 ± 3.11	16.5 ± 3.31	3.3×10^{-6}
Usual care[a]	78	1.37 (31.15)	52.05 ± 5.89	0.71	8.46 ± 3.65	0.02	3.17 ± 3.68	49.18 ± 5.45	0.39
CCI-web vs. CCI-onsite[b]		3.37 ± 4.8		0.48	3.47 ± 3.99	0.38	3.01 ± 4.46		0.5
CCI-web vs. usual care[b]		-12.91 ± 5.01		0.01	-22.17 ± 4.69	2.3×10^{-6}	-14.63 ± 4.87		0.003
CCI-onsite vs. usual care[b]		-16.28 ± 4.95		0.001	-25.64 ± 4.7	4.9×10^{-8}	-17.64 ± 4.82		0.0003
CCI-all vs. usual care[b]		-14.67 ± 4.36		0.0008	-23.89 ± 4.24	1.8×10^{-8}	-16.19 ± 4.3		0.0002
**Thiazolidinedione (%)**									
CCI-all education[a]	218	-1.38 (15.12)	0.46 ± 0.46	0.18	-1.47 ± 0.9	0.1	-1.1 ± 0.91	0.42 ± 0.49	0.23
CCI-web[a]	104	-2.88 (16.82)	0.0 ± 0.0	0.08	-2.6 ± 1.29	0.04	-2.39 ± 1.43	-0.07 ± 0.45	0.1

CCI-onsite[a]	114	0.0 (13.3)	0.88 ± 0.88	1	-0.42 ± 1.25	0.74	0.1 ± 1.13	0.87 ± 0.84	0.93
Usual care[a]	78	0.0 (0.0)	1.37 ± 1.37		0.26 ± 1.64	0.87	0.22 ± 0.51	1.27 ± 1.26	0.67
CCI-web vs. CCI-onsite[b]		-2.88 ± 2.07		0.16	-2.18 ± 1.79	0.22	-2.49 ± 1.82		0.17
CCI-web vs. usual care[b]		-2.88 ± 1.65		0.08	-2.87 ± 2.11	0.17	-2.6 ± 1.52		0.09
CCI-onsite vs. usual care[b]		0.0 ± 1.25		1	-0.68 ± 2.11	0.75	-0.12 ± 1.24		0.93
CCI-all vs. usual care[b]		-1.38 ± 1.02		0.18	-1.78 ± 1.91	0.35	-1.31 ± 1.04		0.21
**SGLT-2 (%)**									
CCI-all education[a]	218	-9.63 (29.57)	0.92 ± 0.65	$1.5x10^{-6}$	-9.96 ± 2.04	$1.1x10^{-6}$	-9.26 ± 1.88	0.92 ± 1.88	$9.0x10^{-7}$
CCI-web[a]	104	-7.69 (26.78)	0.0 ± 0.0	0.003	-7.41 ± 2.92	0.01	-7.0 ± 2.45	1.75 ± 1.23	0.004
CCI-onsite[a]	114	-11.4 (31.93)	1.75 ± 1.24	0.0001	-12.34 ± 2.83	$1.2x10^{-5}$	-11.35 ± 2.82	0.0 ± 0.0	$5.6x10^{-5}$
Usual care[a]	78	0.0 (28.87)	15.07 ± 4.22	1	1.13 ± 3.72	0.76	0.87 ± 3.17	15.07 ± 4.21	0.78
CCI-web vs. CCI-onsite[b]		3.71 ± 3.98		0.35	4.93 ± 4.06	0.22	4.34 ± 3.73		0.24
CCI-web vs. usual care[b]		-7.69 ± 4.28		0.07	-8.55 ± 4.77	0.07	-7.87 ± 4.01		0.05
CCI-onsite vs. usual care[b]		-11.4 ± 4.51		0.01	-13.48 ± 4.78	0.005	-12.22 ± 4.24		0.004
CCI-all vs. usual care[b]		-9.63 ± 3.93		0.01	-11.0 ± 4.32	0.01	-10.13 ± 3.69		0.006
**DPP-4 (%)**									
CCI-all education[a]	218	-3.67 (34.42)	6.42 ± 1.66	0.12	-3.69 ± 2.21	0.09	-3.52 ± 2.21	6.29 ± 1.66	0.11
CCI-web[a]	104	-2.88 (35.41)	6.73 ± 2.47	0.41	-3.1 ± 3.18	0.33	-3.42 ± 3.29	6.65 ± 2.44	0.3
CCI-onsite[a]	114	-4.39 (33.63)	6.14 ± 2.26	0.16	-4.24 ± 3.07	0.17	-3.61 ± 2.99	5.96 ± 2.27	0.23
Usual care[a]	78	2.74 (23.41)	10.96 ± 3.68	0.32	2.97 ± 4.05	0.46	2.64 ± 2.92	10.74 ± 3.51	0.37
CCI-web vs. CCI-onsite[b]		1.5 ± 4.69		0.75	1.14 ± 4.42	0.8	0.19 ± 4.45		0.97
CCI-web vs. usual care[b]		-5.62 ± 4.42		0.2	-6.07 ± 5.19	0.24	-6.06 ± 4.4		0.17
CCI-onsite vs. usual care[b]		-7.13 ± 4.17		0.09	-7.21 ± 5.2	0.17	-6.25 ± 4.18		0.13
CCI-all vs. usual care[b]		-6.41 ± 3.6		0.07	-6.64 ± 4.7	0.16	-6.16 ± 3.66		0.09
**GLP-1 (%)**									
CCI-all education[a]	218	0.92 (34.6)	13.76 ± 2.34	0.7	1.15 ± 2.31	0.62	0.98 ± 2.3	14.4 ± 2.29	0.67
CCI-web[a]	104	-0.96 (32.67)	10.58 ± 3.03	0.76	-0.26 ± 3.33	0.94	-1.14 ± 3.25	11.21 ± 3.07	0.73
CCI-onsite[a]	114	2.63 (36.34)	16.67 ± 3.51	0.44	2.47 ± 3.22	0.44	2.95 ± 3.26	17.35 ± 3.37	0.37
Usual care[a]	78	2.74 (33.22)	19.18 ± 4.64	0.48	2.09 ± 4.23	0.62	2.94 ± 3.84	17.02 ± 4.39	0.44
CCI-web vs. CCI-onsite[b]		-3.59 ± 4.67		0.44	-2.73 ± 4.62	0.55	-4.09 ± 4.6		0.37
CCI-web vs. usual care[b]		-3.7 ± 5.04		0.46	-2.35 ± 5.43	0.67	-4.08 ± 5.03		0.42
CCI-onsite vs. usual care[b]		-0.11 ± 5.17		0.98	0.38 ± 5.44	0.94	0.01 ± 5.04		0.99
CCI-all vs. usual care[b]		-1.82 ± 4.54		0.69	-0.99 ± 4.91	0.84	-1.96 ± 4.48		0.66
**Metformin (%)**									
CCI-all education[a]	218	-7.34 (46.45)	64.22 ± 3.25	0.02	-7.14 ± 3.0	0.02	-6.34 ± 3.06	65.18 ± 3.14	0.04

CCI-web[a]	104	-7.69 (47.65)	63.46 ± 4.74	0.1	-6.7 ± 4.33	0.12	-5.96 ± 4.51	63.74 ± 4.59	0.19
CCI-onsite[a]	114	-7.02 (45.54)	64.91 ± 4.49	0.1	-7.55 ± 4.18	0.07	-6.69 ± 4.13	66.52 ± 4.33	0.11
Usual care[a]	78	0.0 (37.27)	61.64 ± 5.73	1	0.83 ± 5.5	0.88	-0.08 ± 4.55	60.67 ± 5.61	0.99
CCI-web vs. CCI-onsite[b]		-0.67 ± 6.33		0.92	0.85 ± 6.01	0.89	0.73 ± 6.12		0.91
CCI-web vs. usual care[b]		-7.69 ± 6.39		0.23	-7.52 ± 7.06	0.29	-5.88 ± 6.41		0.36
CCI-onsite vs. usual care[b]		-7.02 ± 6.1		0.25	-8.37 ± 7.07	0.24	-6.61 ± 6.15		0.28
CCI-all vs. usual care[b]		-7.34 ± 5.38		0.17	-7.95 ± 6.38	0.21	-6.26 ± 5.48		0.25
**Statin (%)**									
CCI-all education[a]	218	-3.67 (34.42)	48.17 ± 3.39	0.12	-3.64 ± 2.42	0.13	-3.28 ± 2.35	46.66 ± 3.23	0.16
CCI-web[a]	104	-3.85 (36.67)	47.12 ± 4.92	0.28	-3.65 ± 3.5	0.3	-2.76 ± 3.56	44.81 ± 4.67	0.44
CCI-onsite[a]	114	-3.51 (32.4)	49.12 ± 4.7	0.25	-3.64 ± 3.37	0.28	-3.75 ± 3.12	48.37 ± 4.5	0.23
Usual care[a]	73	9.59 (37.88)	64.38 ± 5.64	0.03	9.51 ± 4.3	0.03	8.77 ± 4.3	67.38 ± 5.42	0.04
CCI-web vs. CCI-onsite[b]		-0.34 ± 4.7		0.94	-0.01 ± 4.85	0.99	0.99 ± 4.73		0.83
CCI-web vs. usual care[b]		-13.44 ± 5.71		0.02	-13.15 ± 5.59	0.02	-11.53 ± 5.58		0.04
CCI-onsite vs. usual care[b]		-13.1 ± 5.37		0.01	-13.15 ± 5.58	0.02	-12.53 ± 5.31		0.02
CCI-all vs. usual care[b]		-13.26 ± 5.01		0.008	-13.15 ± 5.02	0.009	-12.05 ± 4.9		0.01
**Blood pressure medication (%)**									
CCI-all education[a]	218	-11.93 (42.34)	56.42 ± 3.37	3.2×10^{-5}	-11.94 ± 2.89	3.6×10^{-5}	-11.37 ± 2.84	55.75 ± 3.3	6.3×10^{-5}
CCI-web[a]	104	-9.62 (40.67)	55.77 ± 4.89	0.02	-9.85 ± 4.16	0.02	-9.8 ± 4.01	55.24 ± 4.75	0.01
CCI-onsite[a]	114	-14.04 (43.88)	57.02 ± 4.66	0.0006	-13.87 ± 4.01	0.0005	-12.82 ± 4.02	56.22 ± 4.61	0.001
Usual care[a]	73	9.59 (41.38)	60.27 ± 5.77	0.05	9.62 ± 5.12	0.06	8.34 ± 4.82	61.18 ± 5.6	0.08
CCI-web vs. CCI-onsite[b]		4.42 ± 5.73		0.44	4.02 ± 5.78	0.49	3.01 ± 5.68		0.6
CCI-web vs. usual care[b]		-19.2 ± 6.27		0.002	-19.53 ± 6.66	0.004	-18.14 ± 6.27		0.004
CCI-onsite vs. usual care[b]		-23.62 ± 6.35		0.0002	-23.55 ± 6.64	0.0005	-21.15 ± 6.27		0.0007
CCI-all vs. usual care[b]		-21.52 ± 5.63		0.0001	-21.55 ± 5.98	0.0004	-19.7 ± 5.59		0.0004
**ACE or ARB (%)**									
CCI-all education[a]	218	0.92 (27.14)	28.9 ± 3.08	0.62	1.53 ± 1.88	0.42	0.68 ± 1.86	29.95 ± 2.94	0.71
CCI-web[a]	104	-0.96 (26.05)	29.81 ± 4.51	0.71	-0.49 ± 2.71	0.86	-1.18 ± 2.62	31.26 ± 4.3	0.65
CCI-onsite[a]	114	2.63 (28.1)	28.07 ± 4.23	0.32	3.4 ± 2.62	0.19	2.41 ± 2.63	28.73 ± 4.04	0.36
Usual care[a]	73	5.48 (28.34)	21.92 ± 4.88	0.1	3.66 ± 3.34	0.27	4.94 ± 3.29	23.37 ± 4.73	0.13
CCI-web vs. CCI-onsite[b]		-3.59 ± 3.67		0.33	-3.89 ± 3.76	0.3	-3.59 ± 3.71		0.33
CCI-web vs. usual care[b]		-6.44 ± 4.19		0.12	-4.09 ± 4.34	0.35	-6.13 ± 4.21		0.15
CCI-onsite vs. usual care[b]		-2.85 ± 4.23		0.5	-0.19 ± 4.33	0.96	-2.54 ± 4.21		0.55
CCI-all vs. usual care[b]		-4.56 ± 3.79		0.23	-2.13 ± 3.9	0.59	-4.26 ± 3.78		0.26

**Diuretics (%)**									
CCI-all education[a]	218	-9.63 (41.28)	31.65 ± 3.16	0.0006	-9.52 ± 2.69	0.0004	-9.55 ± 2.73	31.17 ± 3.07	0.0005
CCI-web[a]	104	-10.58 (39.21)	30.77 ± 4.55	0.006	-10.84 ± 3.88	0.005	-10.53 ± 3.81	29.86 ± 4.42	0.006
CCI-onsite[a]	114	-8.77 (43.24)	32.46 ± 4.4	0.04	-8.3 ± 3.74	0.03	-8.65 ± 3.9	32.38 ± 4.29	0.03
Usual care[a]	73	5.48 (32.87)	30.14 ± 5.41	0.16	5.15 ± 4.77	0.28	3.16 ± 4.07	33.04 ± 5.3	0.44
CCI-web vs. CCI-onsite[b]		-1.81 ± 5.58		0.75	-2.53 ± 5.39	0.64	-1.88 ± 5.45		0.73
CCI-web vs. usual care[b]		-16.06 ± 5.44		0.003	-15.95 ± 6.21	0.01	-13.69 ± 5.57		0.01
CCI-onsite vs. usual care[b]		-14.25 ± 5.59		0.01	-13.42 ± 6.2	0.03	-11.81 ± 5.64		0.04
CCI-all vs. usual care[b]		-15.11 ± 4.76		0.001	-14.68 ± 5.58	0.009	-12.71 ± 4.9		0.009

[a] Means (standard deviations) are presented. Sample sizes, means, and significance levels refer to subjects with baseline and one-year measurements for *completers*, and to 349 subjects (262 intervention and 87 usual care) for *all starters*. Significance levels for *completers* refer to one-sample t-test with or without adjustment. Untransformed triglyceride and hsC-reactive protein values are presented, however, their statistical significances were based on their log-transformed values. All CC refers to the CCI-web and CCI-onsite combined.

[b] Mean differences ± one standard error. Significance levels refer to two-sample t-test or analysis of covariance for the differences.

[c] Adjusted for sex, age, baseline BMI, baseline insulin use (user vs. non-user), and African-American race.

[d] Imputed values based on 700 iterations from multivariate normal regression.

[e] A significance level of $P<0.0017$ ensures overall simultaneous significance of $P \leq 0.05$ over the 30 variables using Bonferroni correction.

Table S3. Effect of the intervention during the first 70 and next 295 days.

	N	Mean change ± SE 1 year	First 70 days	Next 295 days	Significance of change[d] 1 year	First 70 days	Next 295 days	% Effect of first 70 days
**ΔBeta-hydroxybutyrate (mmol·L^{-1})**								
All starters (Dropouts imputed)[a]	262	0.12 ± 0.02	0.37 ± 0.04	-0.24 ± 0.04	$5.8x10^{-7}$	$<10^{-16}$	$1.3x10^{-10}$	296%
Completers								
CCI-all education[b]	172	0.13 ± 0.03	0.37 ± 0.04	-0.24 ± 0.04	$7.3x10^{-7}$	$<10^{-16}$	$4.9x10^{-9}$	278%
CCI-web[b]	79	0.14 ± 0.04	0.34 ± 0.06	-0.2 ± 0.06	0.0003	$2.0x10^{-8}$	0.0005	244%
CCI-onsite[b]	93	0.13 ± 0.04	0.4 ± 0.06	-0.27 ± 0.06	0.0006	$4.6x10^{-12}$	$2.1x10^{-6}$	309%
Difference: web-onsite[c]		0.01 ± 0.05	-0.06 ± 0.08	0.07 ± 0.08				
**ΔHemoglobin A_{1c} (mmol·mol^{-1})**								
All starters (Dropouts imputed)[a]	262	-14.21 ± 0.98	-12.02 ± 0.77	-2.3 ± 0.55	$<10^{-16}$	$<10^{-16}$	$7.7x10^{-5}$	84%
Completers								
CCI-all education[b]	199	-14.1 ± 0.98	-11.91 ± 0.87	-2.3 ± 0.55	$<10^{-16}$	$<10^{-16}$	$6.2x10^{-5}$	84%
CCI-web[b]	96	-13.12 ± 1.2	-12.24 ± 1.09	-0.77 ± 0.77	$<10^{-16}$	$<10^{-16}$	0.3	93%
CCI-onsite[b]	103	-15.19 ± 1.64	-11.59 ± 1.31	-3.61 ± 0.77	$<10^{-16}$	$<10^{-16}$	$5.4x10^{-6}$	76%
Difference: web-onsite[c]		2.08 ± 2.08	-0.77 ± 1.64	2.84 ± 1.09†				
**ΔHemoglobin A_{1c} (%)**								
All starters (Dropouts imputed)[a]	262	-1.30 ± 0.09	-1.1 ± 0.07	-0.21 ± 0.05	$<10^{-16}$	$<10^{-16}$	$7.7x10^{-5}$	84%
Completers								
CCI-all education[b]	199	-1.29 ± 0.09	-1.09 ± 0.08	-0.21 ± 0.05	$<10^{-16}$	$<10^{-16}$	$6.2x10^{-5}$	84%
CCI-web[b]	96	-1.20 ± 0.11	-1.12 ± 0.10	-0.07 ± 0.07	$<10^{-16}$	$<10^{-16}$	0.3	93%
CCI-onsite[b]	103	-1.39 ± 0.15	-1.06 ± 0.12	-0.33 ± 0.07	$<10^{-16}$	$<10^{-16}$	$5.4x10^{-6}$	76%
Difference: web-onsite[c]		0.19 ± 0.19	-0.07 ± 0.15	0.26 ± 0.10†				
**ΔFasting glucose (mmol·L^{-1})**								
All starters (Dropouts imputed)[a]	262	-1.95 ± 0.23	-1.76 ± 0.21	-0.19 ± 0.17	$<10^{-16}$	$<10^{-16}$	0.25	90%
Completers								
CCI-all education[b]	196	-1.97 ± 0.23	-1.76 ± 0.22	-0.2 ± 0.15	$<10^{-16}$	$8.9x10^{-16}$	0.17	89%
CCI-web[b]	95	-1.89 ± 0.31	-1.51 ± 0.27	-0.38 ± 0.17	$8.6x10^{-10}$	$2.6x10^{-8}$	0.03	79%
CCI-onsite[b]	101	-2.04 ± 0.34	-2.01 ± 0.34	-0.03 ± 0.24	$1.6x10^{-9}$	$3.5x10^{-9}$	0.89	98%
Difference: web-onsite[c]		0.16 ± 0.46	0.5 ± 0.43	-0.35 ± 0.29				
**ΔInsulin, all (pmol·L^{-1})**								
All starters (Dropouts imputed)[a]	262	-73.62 ± 12.5	-59.24 ± 7.92	-14.38 ± 12.08	$4.3x10^{-9}$	$6.1x10^{-14}$	0.23	81%

Completers								
CCI-all education[b]	172	-74.45 ± 13.68	-63.82 ± 8.89	-10.63 ± 12.78	5.1x10^{-8}	6.5x10^{-13}	0.41	85%
CCI-web[b]	79	-48.96 ± 23.82	-66.05 ± 11.18	17.15 ± 23.89	0.04	3.4x10^{-9}	0.47	135%
CCI-onsite[b]	93	-96.19 ± 14.86	-61.95 ± 13.47	-34.24 ± 11.74	1.0x10^{-10}	4.1x10^{-6}	0.004	64%
Difference: web-onsite[c]		47.23 ± 28.13	-4.1 ± 17.5	51.39 ± 26.6*				
**ΔInsulin, excluding exogenous users (pmol·L^{-1})**								
All starters (Dropouts imputed)[a]	262	-12.73 ± 1.57	-9.31 ± 1.37	-3.42 ± 1.22	6.6x10^{-16}	1.0x10^{-11}	0.005	73%
Completers								
CCI-all education[b]	123	-13.01 ± 1.43	-9.38 ± 1.41	-3.63 ± 1.31	<10^{-16}	2.7x10^{-11}	0.005	72%
CCI-web[b]	53	-10.56 ± 1.50	-10.21 ± 2.16	-0.35 ± 1.32	1.8x10^{-12}	2.4x10^{-6}	0.79	96%
CCI-onsite[b]	70	-14.87 ± 2.23	-8.75 ± 1.86	-6.12 ± 2.02	2.7x10^{-11}	2.7x10^{-11}	0.002	58%
Difference: web-onsite[c]		4.31 ± 2.69	-1.46 ± 2.86	5.77 ± 2.42*				
**ΔC-peptide (nmol·L^{-1})**								
All starters (Dropouts imputed)[a]	262	-0.33 ± 0.04	-0.22 ± 0.04	-0.12 ± 0.04	2.2x10^{-16}	2.6x10^{-9}	0.001	64%
Completers								
CCI-all education[b]	171	-0.34 ± 0.04	-0.22 ± 0.04	-0.12 ± 0.04	2.0x10^{-15}	2.3x10^{-7}	0.001	65%
CCI-web[b]	79	-0.38 ± 0.06	-0.29 ± 0.07	-0.08 ± 0.05	4.9x10^{-11}	5.1x10^{-6}	0.11	78%
CCI-onsite[b]	92	-0.31 ± 0.06	-0.15 ± 0.06	-0.15 ± 0.05	9.0x10^{-7}	0.005	0.004	50%
Difference: web-onsite[c]		-0.07 ± 0.09	-0.14 ± 0.09	0.07 ± 0.08				
**ΔHOMA-IR (insulin derived), all**								
All starters (Dropouts imputed)[a]	262	-5.58 ± 0.86	-4.96 ± 0.65	-0.62 ± 0.62	7.5x10^{-11}	3.2x10^{-14}	0.31	89%
Completers								
CCI-all education[b]	160	-5.5 ± 0.93	-5.16 ± 0.7	-0.34 ± 0.62	3.8x10^{-9}	1.5x10^{-13}	0.59	94%
CCI-web[b]	74	-4.09 ± 1.43	-4.58 ± 0.84	0.5 ± 1.24	0.004	5.0x10^{-8}	0.69	112%
CCI-onsite[b]	86	-6.72 ± 1.21	-5.66 ± 1.08	-1.06 ± 0.44	3.2x10^{-8}	1.7x10^{-7}	0.02	84%
Difference: web-onsite[c]		2.63 ± 1.88	1.07 ± 1.37	1.55 ± 1.31				
**ΔHOMA-IR (insulin derived), excluding exogenous insulin users**								
All starters (Dropouts imputed)[a]	262	-6.82 ± 0.9	-5.93 ± 0.85	-0.88 ± 0.32	3.2x10^{-14}	3.1x10^{-12}	0.006	87%
Completers								
CCI-all education[b]	123	-13.01 ± 1.43	-9.38 ± 1.41	-3.63 ± 1.31	1.8x10^{-12}	2.7x10^{-11}	0.005	72%
CCI-web[b]	53	-10.56 ± 1.50	-10.21 ± 2.16	-0.35 ± 1.32	1.8x10^{-12}	2.0x10^{-6}	0.79	97%
CCI-onsite[b]	70	-14.87 ± 2.23	-8.75 ± 1.86	-6.12 ± 2.02	2.7x10^{-11}	2.7x10^{-6}	0.002	59%
Difference: web-onsite[c]		4.31 ± 2.69	-1.46 ± 2.86	5.77 ± 2.42*				

**ΔHOMA-IR (C-peptide derived)**								
All starters (Dropouts imputed)[a]	262	-3.45 ± 0.46	-2.57 ± 0.37	-0.88 ± 0.32	$1.0x10^{-13}$	$3.1x10^{-12}$	0.006	74%
Completers								
CCI-all education[b]	150	-3.54 ± 0.45	-2.58 ± 0.41	-0.97 ± 0.26	$4.0x10^{-15}$	$2.8x10^{-10}$	0.0002	73%
CCI-web[b]	69	-3.47 ± 0.67	-2.74 ± 0.62	-0.73 ± 0.4	$2.2x10^{-7}$	$8.4x10^{-6}$	0.07	79%
CCI-onsite[b]	81	-3.6 ± 0.61	-2.44 ± 0.55	-1.17 ± 0.35	$4.2x10^{-9}$	$8.9x10^{-6}$	0.0007	68%
Difference: web-onsite[c]		0.13 ± 0.91	-0.31 ± 0.82	0.44 ± 0.53				
**ΔWeight-clinic (kg)**								
All starters (Dropouts imputed)[a]	262	-13.8 ± 0.71	-8.58 ± 0.35	-5.22 ± 0.55	$<10^{-16}$	$<10^{-16}$	$<10^{-16}$	62%
Completers								
CCI-all education[b]	161	-14.08 ± 0.81	-8.92 ± 0.39	-11.38 ± 1.3	$<10^{-16}$	$<10^{-16}$	$<10^{-16}$	63%
CCI-web[b]	61	-13.17 ± 1.2	-9.55 ± 0.57	-7.98 ± 2.01	$<10^{-16}$	$<10^{-16}$	$7.1x10^{-5}$	73%
CCI-onsite[b]	100	-13.17 ± 1.2	-8.54 ± 0.52	-13.45 ± 1.67	$<10^{-16}$	$<10^{-16}$	$8.9 x10^{-16}$	58%
Difference: web-onsite[c]		1.47 ± 1.61	-1.01 ± 0.77	5.48 ± 2.61*	$<10^{-16}$	$<10^{-16}$	$<10^{-16}$	63%
**ΔWeight-home scale (kg)**								
All starters (Dropouts imputed)[a]	262	-14.36 ± 0.71	-8.63 ± 0.33	-5.73 ± 0.54	$<10^{-16}$	$<10^{-16}$	$<10^{-16}$	60%
Completers								
CCI-all education[b]	218	-15.61 ± 0.73	-8.06 ± 0.29	-7.54 ± 0.56	$<10^{-16}$	$<10^{-16}$	$<10^{-16}$	51%
CCI-web[b]	104	-15.43 ± 1.04	-8.24 ± 0.4	-7.19 ± 0.84	$<10^{-16}$	$<10^{-16}$	$<10^{-16}$	53%
CCI-onsite[b]	114	-15.77 ± 1.03	-7.91 ± 0.42	-7.86 ± 0.76	$<10^{-16}$	$<10^{-16}$	$<10^{-16}$	50%
Difference: web-onsite[c]		0.34 ± 1.47	-0.33 ± 0.58	0.66 ± 1.13				
**ΔBMI-clinic (kg/m^2)**								
All starters (Dropouts imputed)[a]	262	-4.74 ± 0.25	-2.92 ± 0.12	-1.83 ± 0.19	$<10^{-16}$	$<10^{-16}$	$<10^{-16}$	62%
Completers								
CCI-all education[b]	161	-4.93 ± 0.29	-3.11 ± 0.14	-1.83 ± 0.21	$<10^{-16}$	$<10^{-16}$	$<10^{-16}$	63%
CCI-web[b]	61	-4.52 ± 0.41	-3.25 ± 0.19	-1.27 ± 0.31	$<10^{-16}$	$<10^{-16}$	$3.9x10^{-5}$	71%
CCI-onsite[b]	100	-5.18 ± 0.39	-3.02 ± 0.19	-2.16 ± 0.27	$<10^{-16}$	$<10^{-16}$	$2.2x10^{-5}$	58%
Difference: web-onsite[c]		0.66 ± 0.57	-0.23 ± 0.27	0.89 ± 0.41				
**ΔSystolic blood pressure (mmHg)**								
All starters (Dropouts imputed)[a]	262	-6.36 ± 1.12	-5.35 ± 1.08	-1.01 ± 1.08	$1.3x10^{-8}$	$6.7x10^{-7}$	0.35	84%
Completers								
CCI-all education[b]	164	-6.28 ± 1.2	-5.52 ± 1.24	-0.76 ± 1.09	$1.5x10^{-7}$	$7.7x10^{-6}$	0.49	88%
CCI-web[b]	64	-6.34 ± 1.72	-9.66 ± 1.92	3.31 ± 1.77	0.0002	$5.1x10^{-7}$	0.06	152%
CCI-onsite[b]	100	-6.24 ± 1.63	-2.88 ± 1.56	-3.36 ± 1.33	0.0001	0.06	0.01	46%
Difference: web-onsite[c]		-0.1 ± 2.37	-6.78 ± 2.48†	6.67 ± 2.22†				

**ΔDiastolic blood pressure (mmHg)**								
All starters (Dropouts imputed)[a]	262	-3.51 ± 0.65	-4.03 ± 0.62	0.51 ± 0.6	$7.2x10^{-8}$	$7.0x10^{-8}$	0.4	115%
Completers								
CCI-all education[b]	164	-3.27 ± 0.72	-3.77 ± 0.71	0.5 ± 0.62	$4.9x10^{-6}$	$1.1x10^{-7}$	0.42	115%
CCI-web[b]	64	-2.44 ± 1.21	-5.5 ± 1.1	3.06 ± 0.87	0.04	$5.2x10^{-7}$	0.0004	226%
CCI-onsite[b]	100	-3.8 ± 0.88	-2.66 ± 0.92	-1.14 ± 0.8	$1.6x10^{-5}$	0.004	0.16	70%
Difference: web-onsite[c]		1.36 ± 1.5	-2.84 ± 1.43*	4.2 ± 1.19‡				
**ΔTotal cholesterol (mmol·L^{-1})**								
All starters (Dropouts imputed)[a]	262	0.21 ± 0.07	-0.15 ± 0.07	0.36 ± 0.08	0.006	0.02	$1.8x10^{-5}$	-74%
Completers								
CCI-all education[b]	172	0.23 ± 0.07	-0.12± 0.07	0.36 ± 0.08	0.001	0.08	$2.2x10^{-5}$	-52%
CCI-web[b]	79	0.25 ± 0.11	-0.06 ± 0.10	0.31 ± 0.13	0.02	0.55	0.02	-25%
CCI-onsite[b]	85	0.22 ± 0.10	-0.17 ± 0.09	0.40 ± 0.11	0.02	0.07	0.0002	-79%
Difference: web-onsite[c]		0.03 ± 0.13	0.11 ± 0.14	-0.08 ± 0.17				
**ΔLDL-C (mmol·L^{-1})**								
All starters (Dropouts imputed)[a]	262	0.26 ± 0.06	0.06 ± 0.06	0.2 ± 0.07	$5.1x10^{-5}$	0.32	0.005	22%
Completers								
CCI-all education[b]	156	0.28 ± 0.07	0.08 ± 0.06	0.20 ± 0.08	$2.6x10^{-5}$	0.20	0.01	29%
CCI-web[b]	71	0.29 ± 0.10	0.14 ± 0.10	0.15 ± 0.13	0.004	0.13	0.23	49%
CCI-onsite[b]	85	0.27 ± 0.09	0.03 ± 0.08	0.24 ± 0.10	0.003	0.75	0.01	10%
Difference: web-onsite[c]		0.03 ± 0.13	0.12 ± 0.13	-0.09 ± 0.16				
**ΔApo B (g·L^{-1})**								
All starters (Dropouts imputed)[a]	262	-0.02 ± 0.02	-0.04 ± 0.01	0.02 ± 0.02	0.37	0.01	0.3	215%
Completers								
CCI-all education[b]	172	-0.0 ± 0.02	-0.03 ± 0.02	0.03 ± 0.02	0.86	0.07	0.15	931%
CCI-web[b]	79	0.0 ± 0.02	-0.01 ± 0.03	0.01 ± 0.03	0.92	0.67	0.66	-484%
CCI-onsite[b]	93	-0.01 ± 0.03	-0.05 ± 0.02	0.04 ± 0.02	0.78	0.02	0.09	568%
Difference: web-onsite[c]		0.01 ± 0.04	0.03 ± 0.03	-0.02 ± 0.04				
**ΔHDL-C (mmol·L^{-1})**								
All starters (Dropouts imputed)[a]	262	0.2 ± 0.02	0.0 ± 0.02	0.2 ± 0.02	$<10^{-16}$	0.88	$<10^{-16}$	1%
Completers								
CCI-all education[b]	172	0.18 ± 0.02	0.00 ± 0.02	0.18 ± 0.02	$4.4x10^{-16}$	0.89	$<10^{-16}$	-1%
CCI-web[b]	79	0.15 ± 0.04	-0.05 ± 0.03	0.20 ± 0.03	$3.0x10^{-5}$	0.12	$7.7x10^{-10}$	-31%
CCI-onsite[b]	93	0.21 ± 0.03	0.03 ± 0.02	0.18 ± 0.03	$6.9x10^{-14}$	0.16	$1.2x10^{-8}$	16%

Difference: web-onsite[c]		-0.06 ± 0.05	-0.08 ± 0.04*	0.02 ± 0.04				
**ΔTriglycerides (mmol·L^{-1})**								
All starters (Dropouts imputed)[a]	262	-0.54 ± 0.14	-0.47 ± 0.1	-0.07 ± 0.11	<10^{-16}	9.0×10^{-11}	0.0003	87%
Completers								
CCI-all education[b]	172	-0.54 ± 0.15	-0.51 ± 0.12	-0.03 ± 0.10	1.1×10^{-15}	3.5×10^{-9}	0.003	69%
CCI-web[b]	79	-0.33 ± 0.21	-0.27 ± 0.14	-0.06 ± 0.18	2.0×10^{-5}	0.02	0.03	53%
CCI-onsite[b]	93	-0.71 ± 0.21	-0.71 ± 0.19	-0.01± 0.10	1.2×10^{-12}	1.5×10^{-9}	0.04	79%
Difference: web-onsite[c]		0.38 ± 0.30	0.43 ± 0.24	0.05 ± 0.21				
**ΔTotal/HDL-cholesterol**								
All starters (Dropouts imputed)[a]	262	-0.53 ± 0.12	-0.2 ± 0.1	-0.33 ± 0.12	1.7×10^{-5}	0.04	0.004	38%
Completers								
CCI-all education[b]	172	-0.44 ± 0.11	-0.16 ± 0.11	-0.28 ± 0.11	5.3×10^{-5}	0.15	0.008	36%
CCI-web[b]	79	-0.33 ± 0.16	0.05 ± 0.17	-0.38 ± 0.17	0.04	0.75	0.02	-17%
CCI-onsite[b]	93	-0.53 ± 0.15	-0.33 ± 0.13	-0.19 ± 0.13	0.0003	0.01	0.15	63%
Difference: web-onsite[c]		-0.20 ± 0.22	0.39 ± 0.22	-0.19 ± 0.22				
**ΔhsC-reactive protein (nmol·L^{-1})**								
All starters (Dropouts imputed)[a]	262	-34.29 ± 10.0	8.1 ± 6.1	-42.38 ± 10.76	0.0006	0.19	7.8×10^{-5}	-24%
Completers								
CCI-all education[b]	187	-34.48 ± 9.14	3.33 ± 6.19	-37.81 ± 6.67	0.0002	0.59	1.1×10^{-8}	-10%
CCI-web[b]	96	-43.14 ± 17.14	-2.19 ± 10.29	-41.05 ± 10.29	0.01	0.83	6.6×10^{-5}	5%
CCI-onsite[b]	91	-25.33 ± 5.14	9.14 ± 6.67	-34.57 ± 8.29	9.4×10^{-7}	0.17	3.1×10^{-5}	-36%
Difference: web-onsite[c]		-17.81 ± 17.91	-11.33 ± 12.29	-6.48 ± 13.24				
**ΔALT (µkat·L^{-1})**								
All starters (Dropouts imputed)[a]	262	-0.15 ± 0.02	-0.08 ± 0.02	-0.07 ± 0.01	2.4×10^{-10}	0.0001	3.8×10^{-7}	53%
Completers								
CCI-all education[b]	195	-0.16 ± 0.03	-0.09 ± 0.03	-0.08 ± 0.01	1.4×10^{-8}	0.0004	2.7×10^{-7}	54%
CCI-web[b]	95	-0.18 ± 0.03	-0.11 ± 0.03	-0.07 ± 0.02	9.1×10^{-9}	0.0002	0.0006	61%
CCI-onsite[b]	100	-0.15 ± 0.05	-0.07 ± 0.04	-0.08 ± 0.02	0.002	0.1	0.0001	45%
Difference: web-onsite[c]		-0.04 ± 0.06	-0.05 ± 0.05	0.01 ± 0.03				
**ΔAST (µkat·L^{-1})**								
All starters (Dropouts imputed)[a]	262	-0.08 ± 0.02	-0.05 ± 0.02	-0.03 ± 0.01	5.1×10^{-7}	0.001	0.0005	61%
Completers								
CCI-all education[b]	195	-0.09 ± 0.02	-0.06 ± 0.02	-0.03 ± 0.01	3.8×10^{-6}	0.002	0.0006	63%
CCI-web[b]	95	-0.12 ± 0.03	-0.08 ± 0.03	-0.04 ± 0.02	6.0×10^{-5}	0.006	0.02	68%

CCI-onsite[b]	100	-0.06 ± 0.02	-0.03 ± 0.02	-0.03 ± 0.01	0.01	0.12	0.007	53%
Difference: web-onsite[c]		-0.06 ± 0.04	-0.05 ± 0.04	-0.01 ± 0.02				
**ΔAlkaline phosphatase (µkat·L^{-1})**								
All starters (Dropouts imputed)[a]	262	-0.16 ± 0.02	-0.12 ± 0.01	-0.04 ± 0.01	$<10^{-16}$	$<10^{-16}$	0.02	78%
Completers								
CCI-all education[b]	194	-0.17 ± 0.02	-0.13 ± 0.01	-0.04 ± 0.01	$<10^{-16}$	$<10^{-16}$	0.01	78%
CCI-web[b]	95	-0.14 ± 0.02	-0.13 ± 0.02	-0.02 ± 0.02	5.4×10^{-5}	9.2×10^{-10}	0.45	88%
CCI-onsite[b]	99	-0.19 ± 0.03	-0.13 ± 0.02	-0.06 ± 0.02	1.4×10^{-12}	2.9×10^{-12}	0.005	70%
Difference: web-onsite[c]		0.05 ± 0.03	0.01 ± 0.03	0.04 ± 0.03				
**ΔSerum creatinine (µmol·L^{-1})**		-0.04 ± 0.0	-0.04 ± 0.0	0.0 ± 0.0	0.0001	2.1×10^{-6}	0.65	91%
All starters (Dropouts imputed)[a]	262							
Completers								
CCI-all education[b]	196	-0.04 ± 0.01	-0.04 ± 0.01	0.00 ± 0.01	7.6×10^{-5}	3.8×10^{-6}	0.68	93%
CCI-web[b]	95	-0.05 ± 0.01	-0.04 ± 0.01	-0.01 ± 0.01	0.0003	0.0001	0.36	78%
CCI-onsite[b]	101	-0.04 ± 0.02	-0.05 ± 0.01	0.00 ± 0.01	0.02	0.002	0.78	107%
Difference: web-onsite[c]		0.00 ± 0.02	0.01 ± 0.02	-0.01 ± 0.02				
**ΔBUN (mmol·L^{-1})**								
All starters (Dropouts imputed)[a]	262	0.79 ± 0.17	0.17 ± 0.12	0.62 ± 0.16	5.5×10^{-6}	0.15	7.2×10^{-5}	22.30%
Completers								
CCI-all education[b]	196	0.77 ± 0.18	0.11 ± 0.12	0.66 ± 0.16	1.9×10^{-5}	0.37	3.7×10^{-5}	15%
CCI-web[b]	95	0.45 ± 0.18	0.16 ± 0.17	0.29 ± 0.15	0.01	0.35	0.06	35%
CCI-onsite[b]	101	1.07 ± 0.3	0.07 ± 0.19	1.0 ± 0.27	0.0004	0.7	0.0002	7%
Difference: web-onsite[c]		-0.63 ± 0.35	0.09 ± 0.25	-0.71 ± 0.31				
**ΔGFR (mL·s^{-1}·m^{-2})**								
All starters (Dropouts imputed)[a]	262	0.03 ± 0.01	0.03 ± 0.01	0.0 ± 0.01	0.005	0.002	0.55	84%
Completers								
CCI-all education[b]	196	0.04 ± 0.01	0.03 ± 0.01	0.01 ± 0.01	0.0004	0.0004	0.36	80%
CCI-web[b]	95	0.04 ± 0.01	0.04 ± 0.01	0.01 ± 0.01	0.003	0.001	0.52	80%
CCI-onsite[b]	101	0.04 ± 0.02	0.03 ± 0.01	0.01 ± 0.01	0.04	0.04	0.5	80%
Difference: web-onsite[c]		0.01 ± 0.02	0.01 ± 0.02	0.0 ± 0.02				
**ΔAnion gap (mmol·L^{-1})**								
All starters (Dropouts imputed)[a]	262	0.28 ± 0.14	0.22 ± 0.13	0.06 ± 0.15	0.04	0.09	0.68	77%
Completers								
CCI-all education[b]	195	0.29 ± 0.14	0.31 ± 0.14	-0.02 ± 0.16	0.04	0.03	0.92	105%

CCI-web[b]	95	0.07 ± 0.2	0.01 ± 0.2	0.06 ± 0.22	0.72	0.96	0.78	14%
CCI-onsite[b]	100	0.5 ± 0.2	0.59 ± 0.19	-0.09 ± 0.22	0.01	0.002	0.69	118%
Difference: web-onsite[c]		-0.43 ± 0.29	-0.58 ± 0.27*	0.15 ± 0.31				
**ΔCO_2 (mmol·L^{-1})**								
All starters (Dropouts imputed)[a]	262	0.18 ± 0.16	-0.2 ± 0.16	0.38 ± 0.16	0.27	0.2	0.02	-113%
Completers								
CCI-all education[b]	196	0.13 ± 0.16	-0.27 ± 0.16	0.4 ± 0.16	0.41	0.09	0.01	-204%
CCI-web[b]	95	0.49 ± 0.2	-0.24 ± 0.2	0.74 ± 0.21	0.02	0.24	0.0004	-49%
CCI-onsite[b]	101	-0.21 ± 0.25	-0.3 ± 0.24	0.09 ± 0.25	0.4	0.22	0.72	143%
Difference: web-onsite[c]		0.7 ± 0.32*	0.05 ± 0.32	0.65 ± 0.32				
**ΔUric acid (µmol·L^{-1})**								
All starters (Dropouts imputed)[a]	262	1.78 ± 4.76	30.93 ± 4.16	-28.55 ± 4.76	0.67	6.6x10^{-13}	4.0x10^{-10}	1487%
Completers								
CCI-all education[b]	197	0.0 ± 5.35	28.55 ± 4.76	-28.55 ± 4.76	0.96	1.1x10^{-9}	1.2x10^{-9}	11800%
CCI-web[b]	96	-1.19 ± 7.73	32.72 ± 6.54	-33.31 ± 7.14	0.91	3.1x10^{-7}	3.3x10^{-6}	-3507%
CCI-onsite[b]	101	1.19 ± 6.54	24.39 ± 6.54	-23.2 ± 5.95	0.83	0.0003	8.6x10^{-5}	1817%
Difference: web-onsite[c]		-2.38 ± 10.11	7.73 ± 9.52	-10.11 ± 9.52				
**ΔTSH (mIU·L^{-1})**								
All starters (Dropouts imputed)[a]		-0.42 ± 0.1	-0.27 ± 0.1	-0.15 ± 0.08	6.0x10^{-5}	0.008	0.06	64%
Completers								
CCI-all education[b]	195	-0.41 ± 0.12	-0.29 ± 0.11	-0.12 ± 0.08	0.0005	0.008	0.13	72%
CCI-web[b]	96	-0.44 ± 0.19	-0.41 ± 0.17	-0.03 ± 0.12	0.02	0.02	0.78	92%
CCI-onsite[b]	99	-0.38 ± 0.14	-0.18 ± 0.14	-0.2 ± 0.09	0.009	0.19	0.04	48%
Difference: web-onsite[c]		-0.07 ± 0.24	-0.23 ± 0.22	0.16 ± 0.16				
**ΔFree T4 (pmol·L^{-1})**								
All starters (Dropouts imputed)[a]	262	0.13 ± 0.13	0.77 ± 0.13	-0.64 ± 0.13	0.59	2.1x10^{-5}	0.0006	798.90%
Completers								
CCI-all education[b]	197	0.13 ± 0.13	0.77 ± 0.13	-0.64 ± 0.13	0.67	6.4x10^{-5}	0.0004	1030%
CCI-web[b]	96	0.26 ± 0.26	0.9 ± 0.26	-0.77 ± 0.26	0.33	1.1x10^{-5}	0.003	412%
CCI-onsite[b]	101	-0.13 ± 0.26	0.51 ± 0.26	-0.64 ± 0.26	0.76	0.06	0.03	-751%
Difference: web-onsite[c]		0.26 ± 0.39	0.39 ± 0.39	-0.13 ± 0.39				
**ΔAny diabetes medication, excluding metformin (%)**								
All starters (Dropouts imputed)[a]		-27.19 ± 3.14	-22.07 ± 2.67	-5.12 ± 2.34	<10^{-16}	<10^{-16}	0.03	81%

Completers								
CCI-all education[b]	218	-27.52 ± 3.36	-23.39 ± 2.95	-4.13 ± 2.37	2.210^{-16}	$2.0x10^{-15}$	0.08	85%
CCI-web[b]	104	-31.73 ± 4.98	-22.12 ± 4.09	-9.62 ± 3.99	$1.8x10^{-10}$	$6.4x10^{-8}$	0.02	70%
CCI-onsite[b]	114	-23.68 ± 4.54	-24.56 ± 4.24	0.88 ± 2.64	$1.9x10^{-7}$	$6.7x10^{-9}$	0.74	104%
Difference: web-onsite[c]		-8.05 ± 6.74	2.45 ± 5.89	-10.49 ± 4.78*				
**ΔSulfonylurea (%)**								
All starters (Dropouts imputed)[a]		-23.67 ± 2.7	-20.52 ± 2.52	3.15±2.60	$<10^{-16}$	$4.4x10^{-16}$	0.23	86.70%
Completers								
CCI-all education[b]	218	-9.63 ± 2.0	-9.63 ± 2.0	0.0 ± 0.0	$1.5x10^{-6}$	$1.5x10^{-6}$		100%
CCI-web[b]	104	-7.69 ± 2.63	-7.69 ± 2.63	0.0 ± 0.0	0.003	0.003		100%
CCI-onsite[b]	114	-11.4 ± 2.99	-11.4 ± 2.99	0.0 ± 0.0	0.0001	0.0001		100%
Difference: web-onsite[c]		3.71 ± 3.98	3.71 ± 3.98	0.0 ± 0.0				
**ΔInsulin (%)**								
All starters (Dropouts imputed)[a]		-13.03 ± 2.23	-10.31 ± 1.92	-2.72 ± 1.38	$5.0x10^{-9}$	$7.9x10^{-8}$	0.05	79%
Completers								
CCI-all education[b]	218	-13.3 ± 2.4	-11.01 ± 2.12	-2.29 ± 1.37	$2.8x10^{-8}$	$2.2x10^{-7}$	0.09	82.80%
CCI-web[b]	104	-11.54 ± 3.43	-7.69 ± 2.63	-3.85 ± 2.34	0.0008	0.003	0.1	66.70%
CCI-onsite[b]	114	-14.91 ± 3.35	-14.04 ± 3.27	-0.88 ± 1.52	$8.6x10^{-8}$	$1.7x10^{-5}$	0.56	94.10%
Difference: web-onsite[c]		3.37 ± 4.8	6.34 ± 4.19	-2.97 ± 2.79				
**ΔThiazolidinedione (%)**								
All starters (Dropouts imputed)[a]		-1.11 ± 0.9	-0.38 ± 0.4	-0.73 ± 0.83	0.22	0.34	0.38	34.20%
Completers								
CCI-all education[b]	218	-1.38 ± 1.02	-0.46 ± 0.46	-0.92 ± 0.92	0.18	0.32	0.32	33.30%
CCI-web[b]	104	-2.88 ± 1.65	-0.96 ± 0.96	-1.92 ± 1.35	0.08	0.32	0.16	33.30%
CCI-onsite[b]	114	0.00 ± 1.25	0.00 ± 0.00	0.00 ± 1.25				
Difference: web-onsite[c]		-2.88 ± 2.07	-0.96 ± 0.96	-1.92 ± 1.84				
**ΔSGLT-2 (%)**								
All starters (Dropouts imputed)[a]		-9.26 ± 1.88	-8.77 ± 1.85	-0.49±1.87	$9.0x10^{-7}$	$2.0x10^{-6}$	0.79	94.70%
Completers								
CCI-all education[b]	218	-9.63 ± 2.0	-9.63 ± 2.0	0.0 ± 0.0				
CCI-web[b]	104	-7.69 ± 2.63	-7.69 ± 2.63	0.0 ± 0.0	$1.5 x10^{-6}$	$1.5 x10^{-6}$		100%
CCI-onsite[b]	114	-11.4 ± 2.99	-11.4 ± 2.99	0.0 ± 0.0	0.003	0.003		100%
Difference: web-onsite[c]		3.71 ± 3.98	3.71 ± 3.98	0.0 ± 0.0	0.0001	0.0001		100%

**ΔDPP-4 (%)**								
All starters (Dropouts imputed)[a]		-3.63 ± 2.22	-4.26 ± 1.77	0.63 ± 1.66	0.1	0.02	0.7	118%
Completers								
CCI-all education[b]	218	-3.67 ± 2.33	-4.13 ± 1.87	0.46 ± 1.66	0.12	0.03	0.78	113%
CCI-web[b]	104	-2.88 ± 3.47	-4.13 ± 1.87	2.88 ± 2.54	0.41	0.01	0.26	200%
CCI-onsite[b]	114	-4.39 ± 3.15	-2.63 ± 2.91	-1.75 ± 2.15	0.16	0.37	0.41	60%
Difference: web-onsite[c]		1.5 ± 4.69	-3.14 ± 3.71	4.64 ± 3.33				
**ΔGLP-1 (%)**								
All starters (Dropouts imputed)[a]		1.04 ± 2.29	-1.53 ± 1.69	2.57 ± 2.11	0.65	0.36	0.22	-147%
Completers								
CCI-all education[b]	218	0.92 ± 2.34	-1.38 ± 1.78	2.29 ± 2.1	0.7	0.44	0.28	-150%
CCI-web[b]	104	-0.96 ± 3.2	-1.92 ± 1.92	0.96 ± 3.2	0.76	0.32	0.76	200%
CCI-onsite[b]	114	2.63 ± 3.4	-0.88 ± 2.92	3.51 ± 2.77	0.44	0.76	0.2	-33.30%
Difference: web-onsite[c]		-3.59 ± 4.67	-1.05 ± 3.5	-2.55 ± 4.23				
**ΔMetformin (%)**								
All starters (Dropouts imputed)[a]		-6.19 ± 3.07	-0.79 ± 1.7	-5.4 ± 2.79	0.04	0.64	0.05	13%
Completers								
CCI-all education[b]	218	-7.34 ± 3.15	-1.83 ± 1.83	-5.5 ± 2.81	0.02	0.32	0.05	25%
CCI-web[b]	104	-7.69 ± 4.67	-2.88 ± 2.88	-4.81 ± 3.96	0.10	0.32	0.22	38%
CCI-onsite[b]	114	-7.02 ± 4.27	-0.88 ± 2.33	-6.14 ± 4.0	0.10	0.71	0.12	13%
Difference: web-onsite[c]		-0.67 ± 6.33	-2.01 ± 3.71	1.33 ± 5.62				

[a] Imputed values based on 700 iterations from multivariate normal regression.

[b] Mean differences ± one standard error. Sample sizes, means, and significance levels refer to intervention subjects with baseline, 70-day, and one-year measurements for *completers*. Significance levels for *completers* refer to one-sample t-test. Untransformed triglyceride and hsC-reactive protein values are presented, however, their statistical significances were based on their log-transformed values.

[c] Significance levels refer to two-sample t-test of the differences are designated for 0.05>P≥0.01 (*); 0.01>P≥0.001 (†); 0.001>P≥0.0001 (‡); and P<0.0001 (§).

[d] A significance level of P<0.0017 ensures overall simultaneous significance of P≤0.05 over the 30 variables using Bonferroni correction.
