# Supplementary material for: Author Correction: Effectiveness and Safety of a Novel Care Model for the Management of Type 2 Diabetes at 1 Year: An Open-Label, Non-Randomized, Controlled Study
Source: Diabetes Ther. 2018 Mar 5;9(2):613–21. doi: 10.1007/s13300-018-0386-4 (PMC6104276; doi:10.1007/s13300-018-0386-4)
Supplement: Supplementary file 1 — Supplementary material 1 (PDF 206 kb) [file 13300_2018_386_MOESM1_ESM.pdf]

Table S1. Baseline characteristics of the recruited sample, completers, and participants with missing data by treatment arm.

	All		Completers with data		Dropout or missing data		Completers-Dropouts
	N	Mean (SD) or ±SE	N	Mean (SD) or ±SE	N	Mean (SD) or ±SE	Mean ± SE
**Age (years)**							
All	349	53.4 (8.67)	296	53.46 (8.75)	53	53.06 (8.25)	0.4 ± 1.24
CCI-all education[a]	262	53.75 (8.35)	218	54.09 (8.35)	44	52.09 (8.25)	2.0 ± 1.37
CCI-web[a]	126	53.39 (8.19)	104	53.49 (8.23)	22	51.27 (8.44)	0.58 ± 1.92
CCI-onsite[a]	136	54.09 (8.52)	114	54.63 (8.47)	9	57.78 (6.85)	3.36 ± 1.97
Usual care[a]	87	52.33 (9.52)	78	51.71 (9.62)	9	57.78 (6.85)	-6.07 ± 2.53*
CCI-web vs. CCI-onsite[b]		-0.7 ± 1.03		-1.14 ± 1.13		1.64 ± 2.5	
CCI-web vs. usual care[b]		1.06 ± 1.25		1.79 ± 1.36		-4.87 ± 2.87	
CCI-onsite vs. usual care[b]		1.75 ± 1.26		2.93 ± 1.35		-6.51 ± 2.91	
CCI-all vs. usual care[b]		1.42 ± 1.14		2.38 ± 1.23*		-5.69 ± 2.6*	
**Female (%)**							
All	349	64.76 ± 2.55	296	63.85 ± 2.79	53	69.81 ± 6.31	-5.96 ± 6.9
CCI-all education[a]	262	66.79 ± 2.91	218	65.14 ± 3.23	44	75.0 ± 6.53	-9.86 ± 7.28
CCI-web[a]	126	61.9 ± 4.33	104	57.69 ± 4.84	22	81.82 ± 8.22	-24.13 ± 9.54*
CCI-onsite[a]	136	71.32 ± 3.88	114	71.93 ± 4.21	22	68.18 ± 9.93	3.75 ± 10.79
Usual care[a]	87	58.62 ± 5.28	78	60.26 ± 5.54	9	44.44 ± 16.56	15.81 ± 17.47
CCI-web vs. CCI-onsite[b]		-9.42 ± 5.81		-14.24 ± 6.42*		13.64 ± 12.89	
CCI-web vs. usual care[b]		3.28 ± 6.83		-2.56 ± 7.36		37.37 ± 18.49*	
CCI-onsite vs. usual care[b]		12.7 ± 6.55*		11.67 ± 6.96		23.74 ± 19.31	
CCI-all vs. usual care[b]		8.17 ± 6.03		4.88 ± 6.41		30.56 ± 17.8	
**African American (%)**							
All	349	5.16 ± 1.18	296	4.39 ± 1.19	53	9.43 ± 4.02	-5.04 ± 4.19
CCI-all education[a]	262	6.87 ± 1.56	218	5.96 ± 1.6	44	11.36 ± 4.78	-5.4 ± 5.05
CCI-web[a]	126	5.56 ± 2.04	104	5.77 ± 2.29	22	4.55 ± 4.44	1.22 ± 4.99
CCI-onsite[a]	136	8.09 ± 2.34	114	6.14 ± 2.25	22	18.18 ± 8.22	-12.04 ± 8.52
Usual care[a]	87	0.0 ± 0.0	78	0.0 ± 0.0	9	0.0 ± 0.0	0.0 ± 0.0
CCI-web vs. CCI-onsite[b]		-2.53 ± 3.1		-0.37 ± 3.21		-13.64 ± 9.35	
CCI-web vs. usual care[b]		5.56 ± 2.04†		5.77 ± 2.29*		4.55 ± 4.44	
CCI-onsite vs. usual care[b]		8.09 ± 2.34‡		6.14 ± 2.25†		18.18 ± 8.22*	
CCI-all vs. usual care[b]		6.87 ± 1.56§		5.96 ± 1.6‡		11.36 ± 4.78*	

**Years with type 2 diabetes**							
All	332	8.31 (7.23)	288	8.26 (7.28)	44	8.61 (6.97)	-0.35 ± 1.13
CCI-all education[a]	261	8.44 (7.22)	217	8.4 (7.28)	44	8.61 (6.97)	-0.21 ± 1.16
CCI-web[a]	126	7.88 (6.79)	104	7.95 (7.03)	22	7.55 (5.65)	0.41 ± 1.39
CCI-onsite[a]	135	8.96 (7.58)	113	8.81 (7.51)	22	9.68 (8.07)	-0.87 ± 1.86
Usual care[a]	71	7.85 (7.32)	71	7.85 (7.32)		Not collected	
CCI-web vs. CCI-onsite[b]		-1.07 ± 0.89		-0.86 ± 0.99			-2.14 ± 2.1
CCI-web vs. usual care[b]		0.04 ± 1.06		0.11 ± 1.11			
CCI-onsite vs. usual care[b]		1.11 ± 1.09		0.97 ± 1.12			
CCI-all vs. usual care[b]		0.59 (0.9)		0.56 ± 1.0			
**Beta-hydroxybutyrate ($mmol \cdot L^{-1}$)**							
All	327	0.17 (0.15)	245	0.16 (0.14)	82	0.19 (0.16)	-0.02 ± 0.02
CCI-all education[a]	248	0.17 (0.15)	186	0.17 (0.15)	62	0.19 (0.16)	-0.02 ± 0.02
CCI-web[a]	120	0.18 (0.15)	88	0.18 (0.15)	32	0.21 (0.16)	-0.03 ± 0.03
CCI-onsite[a]	128	0.16 (0.16)	98	0.16 (0.15)	30	0.17 (0.16)	-0.01 ± 0.04
Usual care[a]	79	0.15 (0.13)	59	0.14 (0.12)	20	0.17 (0.15)	-0.03 ± 0.03
CCI-web vs. CCI-onsite[b]		0.02 ± 0.02		0.01 ± 0.03		0.03 ± 0.04	
CCI-web vs. usual care[b]		0.03 ± 0.02		0.03 ± 0.03		0.03 ± 0.04	
CCI-onsite vs. usual care[b]		0.01 ± 0.02		0.02 ± 0.03		0.0 ± 0.04	
CCI-all vs. usual care[b]		0.02 ± 0.02		0.02 ± 0.02		0.02 ± 0.04	
**Hemoglobin A_{1c} ($mmol \cdot mol^{-1}$)**							
All	349	59.66 (17.16)	276	59.11 (16.62)	73	61.63 (18.91)	-26.04 ± 2.4
CCI-all education[a]	262	59.55 (16.4)	204	58.35 (15.3)	58	63.49 (19.57)	-28.66 ± 2.73
CCI-web[a]	126	58.68 (15.41)	98	57.69 (13.99)	28	61.85 (19.46)	-27.57 ± 3.94
CCI-onsite[a]	136	60.32 (17.38)	106	59.01 (16.51)	30	65.02 (19.89)	-29.65 ± 3.94
Usual care[a]	87	59.99 (19.24)	72	61.08 (19.89)	15	54.52 (14.87)	-16.97 ± 4.48
CCI-web vs. CCI-onsite[b]		-1.64 ± 2.08		-1.2 ± 2.19		-3.28 ± 5.14	
CCI-web vs. usual care[b]		-1.31 ± 2.51		-3.39 ± 2.73		7.32 ± 5.25	
CCI-onsite vs. usual care[b]		0.33 ± 2.51		-2.19 ± 2.84		10.6 ± 5.25*	
CCI-all vs. usual care[b]		-0.44 ± 2.3		-2.73 ± 2.62		8.96 ± 4.59*	
**Hemoglobin A_{1c} (%)**							
All	349	7.61 (1.57)	276	7.56 (1.52)	73	7.79 (1.73)	-0.23 ± 0.22
CCI-all education[a]	262	7.60 (1.50)	204	7.49 (1.4)	58	7.96 (1.79)	-0.47 ± 0.25
CCI-web[a]	126	7.52 (1.41)	98	7.43 (1.28)	28	7.81 (1.78)	-0.37 ± 0.36
CCI-onsite[a]	136	7.67 (1.59)	106	7.55 (1.51)	30	8.10 (1.82)	-0.56 ± 0.36
Usual care[a]	87	7.64 (1.76)	72	7.74 (1.82)	15	7.14 (1.36)	0.60 ± 0.41
CCI-web vs. CCI-onsite[b]		-0.15 ± 0.19		-0.11 ± 0.2		-0.3 ± 0.47	

CCI-web vs. usual care[b]		-0.12 ± 0.23		-0.31 ± 0.25		0.67 ± 0.48	
CCI-onsite vs. usual care[b]		0.03 ± 0.23		-0.2 ± 0.26		0.97 ± 0.48*	
CCI-all vs. usual care[b]		-0.04 ± 0.21		-0.25 ± 0.24		0.82 ± 0.42*	
**Fasting glucose (mmol·L^{-1})**							
All	344	8.86 (3.57)	273	8.78 (3.46)	71	9.18 (3.96)	-0.4 ± 0.52
CCI-all education[a]	258	8.92 (3.41)	202	8.8 (3.28)	56	9.36 (3.83)	-0.55 ± 0.56
CCI-web[a]	123	8.61 (3.12)	97	8.5 (3.05)	26	9.04 (3.41)	-0.54 ± 0.74
CCI-onsite[a]	135	9.2 (3.64)	105	9.08 (3.47)	30	9.63 (4.21)	-0.55 ± 0.84
Usual care[a]	86	8.67 (4.03)	71	8.71 (3.96)	15	8.5 (4.5)	0.21 ± 1.25
CCI-web vs. CCI-onsite[b]		-0.59 ± 0.42		-0.58 ± 0.46		-0.59 ± 1.02	
CCI-web vs. usual care[b]		-0.05 ± 0.52		-0.21 ± 0.56		0.54 ± 1.34	
CCI-onsite vs. usual care[b]		0.54 ± 0.54		0.38 ± 0.58		1.13 ± 1.39	
CCI-all vs. usual care[b]		0.25 ± 0.48		0.1 ± 0.52		0.86 ± 1.27	
**Insulin all (pmol·L^{-1})**							
All	327	199.25 (167.24)	245	199.81 (172.03)	82	197.59 (153.0)	2.22 ± 20.14
CCI-all education[a]	248	198.35 (165.85)	186	197.65 (167.17)	62	200.5 (163.21)	-2.85 ± 24.1
CCI-web[a]	120	168.07 (110.49)	88	175.99 (121.05)	32	146.26 (71.53)	29.72 ± 18.06
CCI-onsite[a]	128	226.68 (200.92)	98	217.03 (198.35)	30	258.28 (209.46)	-41.25 ± 43.2
Usual care[a]	79	202.17 (172.58)	59	206.68 (187.93)	20	188.77 (119.18)	17.99 ± 36.18
CCI-web vs. CCI-onsite[b]		-58.62 ± 20.42†		-41.04 ± 23.82		-112.02 ± 40.28†	
CCI-web vs. usual care[b]		-34.1 ± 21.88		-30.7 ± 27.64		-42.5 ± 29.52	
CCI-onsite vs. usual care[b]		24.59 ± 26.32		10.35 ± 31.6		69.59 ± 46.6	
CCI-all vs. usual care[b]		-3.82 ± 22.09		-9.1 ± 27.36		11.74 ± 33.75	
**Insulin, excluding exogenous users (pmol·L^{1})**							
All	219	202.86 (167.51)	160	202.79 (174.46)	59	203.0 (148.21)	-0.14 ± 23.75
CCI-all education[a]	176	204.04 (165.01)	135	203.42 (170.22)	41	205.99 (148.55)	-2.57 ± 27.43
CCI-web[a]	84	171.68 (100.98)	60	178.63 (110.36)	24	154.46 (71.53)	24.17 ± 20.42
CCI-onsite[a]	92	233.56 (203.0)	75	223.28 (204.6)	17	278.77 (195.43)	-55.49 ± 52.99
Usual care[a]	43	198.14 (179.18)	25	199.6 (199.81)	18	196.13 (151.61)	3.47 ± 53.62
CCI-web vs. CCI-onsite[b]		-61.81 ± 23.89†		-44.66 ± 27.57		-124.25 ± 49.59†	
CCI-web vs. usual care[b]		-26.39 ± 29.45		-20.97 ± 42.43		-41.67 ± 38.61	
CCI-onsite vs. usual care[b]		35.42 ± 34.59		23.68 ± 46.39		82.65 ± 59.45	
CCI-all vs. usual care[b]		5.9 ± 30.0		3.89 ± 42.57		9.86 ± 42.57	
**C-peptide (nmol·L^{-1})**							
All	326	1.43 (0.74)	244	1.44 (0.74)	82	1.42 (0.72)	0.02 ± 0.09
CCI-all education[a]	247	1.45 (0.71)	185	1.47 (0.72)	62	1.39 (0.69)	0.07 ± 0.1

CCI-web[a]	120	1.38 (0.68)	88	1.4 (0.74)	32	1.32 (0.46)	0.07 ± 0.11
CCI-onsite[a]	127	1.52 (0.73)	97	1.53 (0.69)	30	1.47 (0.87)	0.06 ± 0.17
Usual care[a]	79	1.38 (0.82)	59	1.35 (0.82)	20	1.49 (0.84)	-0.14 ± 0.22
CCI-web vs. CCI-onsite[b]		-0.14 ± 0.09		-0.13 ± 0.11		-0.15 ± 0.18	
CCI-web vs. usual care[b]		-0.01 ± 0.11		0.05 ± 0.13		-0.16 ± 0.21	
CCI-onsite vs. usual care[b]		0.13 ± 0.11		0.18 ± 0.13		-0.02 ± 0.24	
CCI-all vs. usual care[b]		0.07 ± 0.1		0.12 ± 0.12		-0.09 ± 0.21	
**HOMA-IR (insulin derived), all**							
All	322	11.52 (12.28)	235	11.22 (12.14)	87	12.34 (12.7)	-1.12 ± 1.57
CCI-all education[a]	244	11.8 (13.14)	179	11.19 (12.75)	65	13.48 (14.12)	-2.3 ± 1.99
CCI-web[a]	117	9.59 (8.37)	84	9.9 (9.26)	33	8.8 (5.54)	1.1 ± 1.4
CCI-onsite[a]	127	13.84 (16.11)	95	12.33 (15.14)	32	18.32 (18.23)	-5.99 ± 3.58
Usual care[a]	78	10.64 (9.12)	56	11.31 (10.05)	22	8.94 (6.03)	2.36 ± 1.86
CCI-web vs. CCI-onsite[b]		-4.25 ± 1.63†		-2.43 ± 1.85		-9.52 ± 3.36†	
CCI-web vs. usual care[b]		-1.05 ± 1.29		-1.4 ± 1.68		-0.15 ± 1.61	
CCI-onsite vs. usual care[b]		3.2 ± 1.76		1.02 ± 2.05		9.37 ± 3.47†	
CCI-all vs. usual care[b]		1.16 ± 1.33		-0.12 ± 1.65		4.54 ± 2.17	
**HOMA-IR (insulin derived), excluding exogenous users**							
All	215	11.30 (12.96)	154	10.74 (12.94)	61	12.72 (13.01)	-1.98 ± 1.97
CCI-all education[a]	172	11.77 (13.87)	129	11.00 (13.53)	43	14.09 (14.76)	-3.08 ± 2.55
CCI-web[a]	81	9.08 (7.16)	57	9.21 (7.82)	24	8.79 (5.39)	0.41 ± 1.51
CCI-onsite[a]	91	14.17 (17.54)	72	12.42 (16.65)	19	20.77 (19.66)	-8.35 ± 4.92
Usual care[a]	43	9.40 (8.25)	25	9.36 (9.39)	18	9.45 (6.61)	-0.09 ± 2.44
CCI-web vs. CCI-onsite[b]		-5.08 ± 2.00*		-3.22 ± 2.22		-11.98 ± 4.64†	
CCI-web vs. usual care[b]		-0.32 ± 1.49		-0.16 ± 2.14		-0.66 ± 1.91	
CCI-onsite vs. usual care[b]		4.76 ± 2.23*		3.06 ± 2.72		11.32 ± 4.77*	
CCI-all vs. usual care[b]		2.37 ± 1.64		1.64 ± 2.22		4.63 ± 2.74	
**HOMA-IR (C-peptide derived)**							
All	311	11.44 (7.17)	217	11.25 (6.59)	94	11.87 (8.38)	-0.63 ± 0.97
CCI-all education[a]	244	11.52 (7.15)	170	11.44 (6.26)	69	11.72 (9.04)	-0.28 ± 1.19
CCI-web[a]	115	10.52 (5.72)	79	10.75 (6.29)	36	10.01 (4.24)	0.74 ± 1.00
CCI-onsite[a]	124	12.45 (8.18)	91	12.04 (6.20)	33	13.59 (12.13)	-1.55 ± 2.21
Usual care[a]	72	11.16 (7.26)	47	10.56 (7.70)	25	12.29 (6.33)	-1.73 ± 1.69
CCI-web vs. CCI-onsite[b]		-1.93 ± 0.91*		-1.29 ± 0.96		-3.58 ± 2.23	
CCI-web vs. usual care[b]		-0.64 ± 1.01		0.19 ± 1.33		-2.28 ± 1.45	
CCI-onsite vs. usual care[b]		1.29 ± 1.13		1.48 ± 1.30		1.3 ± 2.46	
CCI-all vs. usual care[b]		0.36 ± 0.97		0.88 ± 1.22		-0.56 ± 1.67	

**Weight-clinic (kg)**							
All	340	113.85 (25.47)	253	113.06 (24.24)	87	116.14 (28.77)	-3.08 ± 3.44
CCI-all education[a]	257	116.51 (25.94)	184	115.42 (24.62)	73	119.25 (29.01)	-3.83 ± 3.85
CCI-web[a]	121	114.58 (27.08)	84	114.78 (25.87)	37	114.14 (30.01)	0.64 ± 5.68
CCI-onsite[a]	136	118.22 (24.86)	100	115.96 (23.63)	36	124.5 (27.36)	-8.54 ± 5.14
Usual care[a]	83	105.63 (22.15)	69	106.79 (22.18)	14	99.94 (21.86)	6.84 ± 6.42
CCI-web vs. CCI-onsite[b]		-3.64 ± 3.26		-1.18 ± 3.68		-10.36 ± 6.72	
CCI-web vs. usual care[b]		8.95 ± 3.46†		7.99 ± 3.89*		14.2 ± 7.65	
CCI-onsite vs. usual care[b]		12.58 ± 3.23§		9.17 ± 3.57†		24.55 ± 7.41‡	
CCI-all vs. usual care[b]		10.87 ± 2.92§		8.63 ± 3.23†		19.3 ± 6.76†	
**Weight-home scale (kg)**							
CCI-all education[a]	262	116.4 (26.31)	218	116.29 (25.25)	44	116.94 (31.37)	-0.64 ± 5.03
CCI-web[a]	126	114.52 (27.52)	104	116.05 (27.42)	22	107.26 (27.42)	8.8 ± 6.44
CCI-onsite[a]	136	118.14 (25.12)	114	116.51 (23.21)	22	126.61 (32.65)	-10.11 ± 7.29
CCI-web vs. CCI-onsite[b]		-3.62 ± 3.26		-0.45 ± 3.46		-19.36 ± 9.09*	
**BMI (kg·m^{-2})**							
All	340	39.52 (8.6)	253	39.13 (7.89)	87	40.66 (10.36)	-1.54 ± 1.22
CCI-all education[a]	257	40.43 (8.81)	184	39.87 (7.88)	73	41.82 (10.75)	-1.94 ± 1.39
CCI-web[a]	121	39.36 (9.12)	84	38.82 (7.81)	37	40.6 (11.6)	-1.79 ± 2.09
CCI-onsite[a]	136	41.37 (8.44)	100	40.76 (7.86)	36	43.06 (9.82)	-2.3 ± 1.82
Usual care[a]	83	36.72 (7.26)	69	37.14 (7.62)	14	34.66 (4.8)	2.48 ± 1.58
CCI-web vs. CCI-onsite[b]		-2.01 ± 1.1		-1.95 ± 1.16		-2.46 ± 2.51	
CCI-web vs. usual care[b]		2.64 ± 1.15*		1.68 ± 1.25		5.94 ± 2.3†	
CCI-onsite vs. usual care[b]		4.65 ± 1.08§		3.62 ± 1.21†		8.4 ± 2.08§	
CCI-all vs. usual care[b]		3.7 ± 0.97‡		2.73 ± 1.09†		7.15 ± 1.8§	
**Systolic blood pressure (mmHg)**							
All	339	131.44 (13.99)	254	131.51 (14.14)	85	131.22 (13.6)	0.29 ± 1.72
CCI-all education[a]	260	131.94 (14.09)	187	132.51 (14.54)	73	130.47 (12.84)	2.05 ± 1.84
CCI-web[a]	124	132.66 (14.15)	87	133.22 (14.78)	37	131.35 (12.63)	1.87 ± 2.61
CCI-onsite[a]	136	131.28 (14.06)	100	131.9 (14.38)	36	129.56 (13.16)	2.34 ± 2.62
Usual care[a]	79	129.8 (13.61)	67	128.72 (12.65)	12	135.83 (17.49)	-7.12 ± 5.28
CCI-web vs. CCI-onsite[b]		1.38 ± 1.75		1.32 ± 2.14		1.8 ± 3.02	
CCI-web vs. usual care[b]		2.86 ± 1.99		4.5 ± 2.21*		-4.48 ± 5.46	
CCI-onsite vs. usual care[b]		1.48 ± 1.95		3.18 ± 2.11		-6.28 ± 5.5	
CCI-all vs. usual care[b]		2.14 ± 1.76		3.8 ± 1.88*		-5.37 ± 5.27	

**Diastolic blood pressure (mmHg)**							
All	339	82.07 (8.4)	254	81.46 (8.04)	85	83.88 (9.2)	-2.42 ± 1.12*
CCI-all education[a]	260	82.09 (8.25)	187	81.59 (8.05)	73	83.37 (8.67)	-1.78 ± 1.17
CCI-web[a]	124	82.6 (8.85)	87	81.52 (8.27)	37	85.14 (9.74)	-3.62 ± 1.83*
CCI-onsite[a]	136	81.63 (7.67)	100	81.66 (7.9)	36	81.56 (7.09)	0.1 ± 1.42
Usual care[a]	79	82.0 (8.93)	67	81.1 (8.07)	12	87.0 (11.95)	-5.9 ± 3.59
CCI-web vs. CCI-onsite[b]		0.96 ± 1.03		-0.14 ± 1.19		3.58 ± 1.99	
CCI-web vs. usual care[b]		0.6 ± 1.28		0.41 ± 1.33		-1.86 ± 3.8	
CCI-onsite vs. usual care[b]		-0.37 ± 1.2		0.56 ± 1.26		-5.44 ± 3.65	
CCI-all vs. usual care[b]		0.09 ± 1.13		0.49 ± 1.15		-3.63 ± 3.6	
**Total cholesterol (mmol·L^{-1})**							
All	326	4.76 (1.09)	245	4.69 (1.09)	81	4.96 (1.1)	-0.28 ± 0.14*
CCI-all education[a]	247	4.76 (1.07)	186	4.68 (1.03)	61	4.99 (1.15)	-0.31 ± 0.17
CCI-web[a]	120	4.78 (1.1)	88	4.59 (1.06)	32	5.3 (1.07)	-0.72 ± 0.22‡
CCI-onsite[a]	127	4.73 (1.03)	98	4.76 (1.0)	29	4.65 (1.16)	0.11 ± 0.24
Usual care[a]	79	4.76 (1.19)	59	4.72 (1.26)	20	4.88 (0.93)	-0.16 ± 0.27
CCI-web vs. CCI-onsite[b]		0.04 ± 0.14		-0.17 ± 0.15		0.66 ± 0.29*	
CCI-web vs. usual care[b]		0.02 ± 0.17		-0.13 ± 0.2		0.42 ± 0.28	
CCI-onsite vs. usual care[b]		-0.03 ± 0.16		0.04 ± 0.19		-0.24 ± 0.3	
CCI-all vs. usual care[b]		-0.0 ± 0.15		-0.04 ± 0.18		0.11 ± 0.26	
**LDL-cholesterol (mmol·L^{-1})**							
All	302	2.65 (0.87)	220	2.59 (0.87)	82	2.80 (0.85)	-0.20 ± 0.11
CCI-all education[a]	232	2.66 (0.85)	172	2.59 (0.84)	60	2.84 (0.86)	-0.24 ± 0.13
CCI-web[a]	115	2.67 (0.92)	83	2.52 (0.92)	32	3.07 (0.81)	-0.55 ± 0.18†
CCI-onsite[a]	117	2.64 (0.78)	89	2.66 (0.76)	28	2.57 (0.85)	0.09 ± 0.18
Usual care[a]	70	2.63 (0.94)	48	2.60 (0.98)	22	2.69 (0.85)	-0.09 ± 0.23
CCI-web vs. CCI-onsite[b]		0.03 ± 0.11		-0.15 ± 0.13		0.50 ± 0.21*	
CCI-web vs. usual care[b]		0.04 ± 0.14		-0.08 ± 0.17		0.38 ± 0.23	
CCI-onsite vs. usual care[b]		0.01 ± 0.13		0.06 ± 0.16		-0.12 ± 0.24	
CCI-all vs. usual care[b]		0.03 ± 0.13		-0.01 ± 0.16		0.14 ± 0.21	
**Apo B (g·L^{-1})**							
All	327	1.06 (0.29)	245	1.04 (0.28)	82	1.1 (0.29)	-0.06 ± 0.04
CCI-all education[a]	248	1.05 (0.29)	186	1.03 (0.28)	62	1.1 (0.31)	-0.06 ± 0.04
CCI-web[a]	120	1.08 (0.3)	88	1.03 (0.29)	32	1.21 (0.3)	-0.18 ± 0.06†
CCI-onsite[a]	128	1.02 (0.27)	98	1.04 (0.27)	30	0.97 (0.26)	0.06 ± 0.06
Usual care[a]	79	1.07 (0.28)	59	1.06 (0.3)	20	1.11 (0.24)	-0.05 ± 0.07
CCI-web vs. CCI-onsite[b]		0.06 ± 0.04		-0.01 ± 0.04		0.24 ± 0.07‡	

CCI-web vs. usual care[b]		0.01 ± 0.04		-0.03 ± 0.05		0.11 ± 0.08	
CCI-onsite vs. usual care[b]		-0.05 ± 0.04		-0.02 ± 0.05		-0.13 ± 0.07	
CCI-all vs. usual care[b]		-0.02 ± 0.04		-0.02 ± 0.04		-0.01 ± 0.07	
**HDL-C (mmol·L^{-1})**							
All	326	1.06 (0.34)	245	1.06 (0.35)	81	1.06 (0.31)	0.0 ± 0.04
CCI-all education[a]	247	1.09 (0.35)	186	1.1 (0.36)	61	1.08 (0.32)	0.02 ± 0.05
CCI-web[a]	120	1.1 (0.33)	88	1.1 (0.36)	32	1.1 (0.24)	-0.0 ± 0.06
CCI-onsite[a]	127	1.09 (0.36)	98	1.1 (0.36)	29	1.05 (0.39)	0.04 ± 0.08
Usual care[a]	79	0.97 (0.29)	59	0.96 (0.29)	20	1.02 (0.29)	-0.06 ± 0.08
CCI-web vs. CCI-onsite[b]		0.01 ± 0.04		0.0 ± 0.05		0.05 ± 0.08	
CCI-web vs. usual care[b]		0.12 ± 0.04†		0.14 ± 0.05		0.08 ± 0.08	
CCI-onsite vs. usual care[b]		0.11 ± 0.05*		0.14 ± 0.05†		0.04 ± 0.1	
CCI-all vs. usual care[b]		0.12 ± 0.04†		0.14 ± 0.05†		0.06 ± 0.08	
**Triglycerides (mmol·L^{-1})**							
All	326	2.46 (2.66)	245	2.53 (2.97)	81	2.26 (1.35)	0.27 ± 0.24
CCI-all education[a]	247	2.23 (1.62)	186	2.27 (1.73)	61	2.11 (1.25)	0.15 ± 0.2
CCI-web[a]	120	2.14 (1.17)	88	2.16 (1.2)	32	2.1 (1.1)	0.06 ± 0.23
CCI-onsite[a]	127	2.31 (1.95)	98	2.36 (2.09)	29	2.13 (1.41)	0.23 ± 0.34
Usual care[a]	79	3.2 (4.53)	59	3.36 (5.17)	20	2.72 (1.56)	0.64 ± 0.76
CCI-web vs. CCI-onsite[b]		-0.16 ± 0.2		-0.2 ± 0.25		-0.03 ± 0.33	
CCI-web vs. usual care[b]		-1.05 ± 0.52*		-1.2 ± 0.69		-0.62 ± 0.4	
CCI-onsite vs. usual care[b]		-0.89 ± 0.54*		-1.0 ± 0.71		-0.59 ± 0.44	
CCI-all vs. usual care[b]		-0.97 ± 0.52*		-1.09 ± 0.68		-0.61 ± 0.38*	
**Total/HDL-cholesterol**							
All	326	4.88 (1.92)	245	4.84 (2.0)	81	4.99 (1.66)	-0.15 ± 0.22
CCI-all education[a]	247	4.72 (1.7)	186	4.65 (1.72)	61	4.93 (1.65)	-0.28 ± 0.25
CCI-web[a]	120	4.68 (1.54)	88	4.55 (1.58)	32	5.02 (1.36)	-0.47 ± 0.29
CCI-onsite[a]	127	4.76 (1.85)	98	4.74 (1.83)	29	4.84 (1.94)	-0.1 ± 0.41
Usual care[a]	79	5.37 (2.42)	59	5.44 (2.63)	20	5.17 (1.72)	0.27 ± 0.52
CCI-web vs. CCI-onsite[b]		-0.08 ± 0.22		-0.19 ± 0.25		0.19 ± 0.43	
CCI-web vs. usual care[b]		-0.69 ± 0.31*		-0.88 ± 0.38*		-0.15 ± 0.45	
CCI-onsite vs. usual care[b]		-0.61 ± 0.32		-0.7 ± 0.39		-0.33 ± 0.53	
CCI-all vs. usual care[b]		-0.65 ± 0.29*		-0.79 ± 0.36*		-0.24 ± 0.44	
**hsC-reactive protein (nmol·L^{-1})**							
All	334	82.19 (126.1)	263	86.0 (138.29)	71	68.29 (61.81)	17.71 ± 11.24
CCI-all education[a]	249	81.33 (138.0)	193	85.62 (153.05)	56	66.76 (62.1)	18.86 ± 13.81

CCI-web[a]	126	89.43 (183.72)	98	98.38 (98.38)	28	58.1 (58.1)	40.29 ± 22.1
CCI-onsite[a]	123	73.14 (63.53)	95	72.38 (72.38)	28	75.43 (75.43)	-3.05 ± 16.1
Usual care[a]	85	84.67 (82.1)	70	86.95 (86.95)	15	73.81 (73.81)	13.14 ± 19.14
CCI-web vs. CCI-onsite[b]		16.29 ± 17.33		25.91 ± 21.71		-17.33 ± 16.57	
CCI-web vs. usual care[b]		4.76 ± 18.67		11.43 ± 23.24		-15.71 ± 17.71	
CCI-onsite vs. usual care[b]		-11.52 ± 10.57		-14.57 ± 11.9		1.62 ± 22.0	
CCI-all vs. usual care[b]		-3.24 ± 12.48		-1.33 ± 15.05		-7.05 ± 18.19	
**ALT (µkat·L^{-1})**							
All	343	0.5 (0.37)	272	0.51 (0.39)	71	0.48 (0.27)	0.03 ± 0.04
CCI-all education[a]	257	0.51 (0.38)	201	0.52 (0.41)	56	0.47 (0.27)	0.05 ± 0.05
CCI-web[a]	123	0.53 (0.3)	97	0.56 (0.31)	26	0.45 (0.27)	0.11 ± 0.06
CCI-onsite[a]	134	0.49 (0.44)	104	0.49 (0.48)	30	0.49 (0.27)	0.0 ± 0.07
Usual care[a]	86	0.46 (0.33)	71	0.45 (0.34)	15	0.51 (0.29)	-0.05 ± 0.09
CCI-web vs. CCI-onsite[b]		0.04 ± 0.05		0.06 ± 0.06		-0.04 ± 0.07	
CCI-web vs. usual care[b]		0.07 ± 0.04		0.1 ± 0.05*		-0.06 ± 0.09	
CCI-onsite vs. usual care[b]		0.03 ± 0.05		0.04 ± 0.06		-0.02 ± 0.09	
CCI-all vs. usual care[b]		0.05 ± 0.04		0.07 ± 0.05		-0.04 ± 0.08	
**AST (µkat·L^{-1})**							
All	343	0.4 (0.27)	272	0.4 (0.3)	71	0.37 (0.15)	0.03 ± 0.03
CCI-all education[a]	257	0.4 (0.25)	201	0.41 (0.28)	56	0.36 (0.15)	0.04 ± 0.03
CCI-web[a]	123	0.42 (0.27)	97	0.44 (0.29)	26	0.35 (0.17)	0.09 ± 0.04
CCI-onsite[a]	134	0.37 (0.24)	104	0.37 (0.26)	30	0.37 (0.14)	0.0 ± 0.04
Usual care[a]	86	0.4 (0.32)	71	0.39 (0.35)	15	0.42 (0.16)	-0.03 ± 0.06
CCI-web vs. CCI-onsite[b]		0.05 ± 0.03		0.07 ± 0.04		-0.02 ± 0.04	
CCI-web vs. usual care[b]		0.02 ± 0.04		0.05 ± 0.05		-0.07 ± 0.05	
CCI-onsite vs. usual care[b]		-0.03 ± 0.04		-0.02 ± 0.05		-0.05 ± 0.05	
CCI-all vs. usual care[b]		-0.0 ± 0.04		0.01 ± 0.05		-0.06 ± 0.05	
**Alkaline phosphatase (µkat·L^{-1})**							
All	342	1.25 (0.39)	271	1.26 (0.4)	71	1.23 (0.36)	0.03 ± 0.05
CCI-all education[a]	256	1.24 (0.37)	200	1.24 (0.37)	56	1.23 (0.36)	0.01 ± 0.05
CCI-web[a]	123	1.22 (0.39)	97	1.23 (0.4)	26	1.19 (0.33)	0.04 ± 0.08
CCI-onsite[a]	133	1.25 (0.36)	103	1.25 (0.35)	30	1.27 (0.39)	-0.02 ± 0.08
Usual care[a]	86	1.29 (0.44)	71	1.31 (0.45)	15	1.22 (0.38)	0.09 ± 0.11
CCI-web vs. CCI-onsite[b]		-0.03 ± 0.05		-0.02 ± 0.05		-0.08 ± 0.1	
CCI-web vs. usual care[b]		-0.07 ± 0.06		-0.08 ± 0.07		-0.03 ± 0.12	
CCI-onsite vs. usual care[b]		-0.04 ± 0.06		-0.06 ± 0.06		0.05 ± 0.12	
CCI-all vs. usual care[b]		-0.05 ± 0.05		-0.07 ± 0.06		0.01 ± 0.11	

**Serum creatinine (µmol·L^{-1})**							
All	344	78.68 (21.22)	273	77.79 (20.33)	71	82.21 (24.75)	-4.42 ± 3.54
CCI-all education[a]	258	77.79 (21.22)	202	77.79 (20.33)	56	81.33 (24.75)	-3.54 ± 3.54
CCI-web[a]	123	76.91 (17.68)	97	76.91 (18.56)	26	76.02 (15.91)	0.88 ± 3.54
CCI-onsite[a]	135	79.56 (23.87)	105	77.79 (21.22)	30	85.75 (29.17)	-7.96 ± 6.19
Usual care[a]	86	80.44 (22.1)	71	78.68 (20.33)	15	86.63 (25.64)	-7.07 ± 7.07
CCI-web vs. CCI-onsite[b]		-2.65 ± 2.65		-0.88 ± 2.65		-10.61 ± 6.19	
CCI-web vs. usual care[b]		-3.54 ± 2.65		-1.77 ± 2.65		-10.61 ± 7.07	
CCI-onsite vs. usual care[b]		-0.88 ± 3.54		-0.88 ± 3.54		0.0 ± 8.84	
CCI-all vs. usual care[b]		-1.77 ± 2.65		-1.77 ± 2.65		-5.3 ± 7.07	
**BUN (mmol·L^{-1})**							
All	344	5.95 (2.31)	273	5.94 (2.08)	71	6.0 (3.06)	-0.06 ± 0.39
CCI-all education[a]	258	6.03 (2.34)	202	6.06 (2.15)	56	5.9 (2.96)	0.16 ± 0.42
CCI-web[a]	123	6.02 (1.71)	97	6.17 (1.81)	26	5.42 (1.14)	0.75 ± 0.29†
CCI-onsite[a]	135	6.04 (2.8)	105	5.96 (2.42)	30	6.32 (3.88)	-0.36 ± 0.75
Usual care[a]	86	5.73 (2.23)	71	5.59 (1.86)	15	6.38 (3.52)	-0.79 ± 0.94
CCI-web vs. CCI-onsite[b]		-0.02 ± 0.29		0.21 ± 0.3		-0.9 ± 0.74	
CCI-web vs. usual care[b]		0.29 ± 0.29		0.58 ± 0.29*		-0.95 ± 0.94	
CCI-onsite vs. usual care[b]		0.31 ± 0.34		0.37 ± 0.32		-0.06 ± 1.15	
CCI-all vs. usual care[b]		0.3 ± 0.28		0.47 ± 0.27		-0.47 ± 0.99	
**eGFR (mL·s^{-1}·m^{-2})**							
All	344	1.34 (0.23)	273	1.34 (0.22)	71	1.31 (0.26)	0.03 ± 0.03
CCI-all education[a]	258	1.34 (0.23)	202	1.35 (0.22)	56	1.33 (0.25)	0.02 ± 0.04
CCI-web[a]	123	1.36 (0.21)	97	1.37 (0.2)	26	1.35 (0.23)	0.02 ± 0.05
CCI-onsite[a]	135	1.33 (0.24)	105	1.33 (0.24)	30	1.32 (0.26)	0.01 ± 0.05
Usual care[a]	86	1.32 (0.23)	71	1.34 (0.22)	15	1.26 (0.28)	0.08 ± 0.08
CCI-web vs. CCI-onsite[b]		0.04 ± 0.05		0.04 ± 0.03		0.03 ± 0.07	
CCI-web vs. usual care[b]		0.04 ± 0.03		0.03 ± 0.03		0.09 ± 0.09	
CCI-onsite vs. usual care[b]		0.0 ± 0.03		-0.01 (0.01)		0.06 ± 0.09	
CCI-all vs. usual care[b]		0.02 ± 0.03		0.02 ± 0.03		0.03 ± 0.08	
**Anion gap (mmol·L^{-1})**							
All	343	6.86 (1.7)	272	6.82 (1.73)	71	6.99 (1.6)	-0.16 ± 0.22
CCI-all education[a]	257	6.83 (1.67)	201	6.79 (1.7)	56	6.98 (1.53)	-0.19 ± 0.24
CCI-web[a]	123	6.93 (1.71)	97	6.97 (1.66)	26	6.77 (1.92)	0.2 ± 0.41
CCI-onsite[a]	134	6.75 (1.63)	104	6.63 (1.74)	30	7.17 (1.09)	-0.54 ± 0.26*
Usual care[a]	86	6.93 (1.82)	71	6.92 (1.82)	15	7.0 (1.89)	-0.08 ± 0.53

CCI-web vs. CCI-onsite[b]		0.18 ± 0.21		0.34 ± 0.24		-0.4 ± 0.43	
CCI-web vs. usual care[b]		0.0 ± 0.25		0.05 ± 0.27		-0.23 ± 0.62	
CCI-onsite vs. usual care[b]		-0.18 ± 0.24		-0.29 ± 0.27		0.17 ± 0.53	
CCI-all vs. usual care[b]		-0.1 ± 0.22		-0.12 ± 0.25		-0.02 ± 0.53	
**CO_2 (mmol·L^{-1})**							
All	344	27.86 (2.65)	273	27.97 (2.58)	71	27.42 (2.91)	0.55 ± 0.38
CCI-all education[a]	258	27.78 (2.55)	202	27.95 (2.37)	56	27.18 (3.06)	0.77 ± 0.44
CCI-web[a]	123	27.9 (2.37)	97	27.97 (2.15)	26	27.65 (3.1)	0.32 ± 0.65
CCI-onsite[a]	135	27.67 (2.7)	105	27.92 (2.56)	30	26.77 (3.01)	1.16 ± 0.6
Usual care[a]	86	28.1 (2.95)	71	28.06 (3.11)	15	28.33 (2.09)	-0.28 ± 0.65
CCI-web vs. CCI-onsite[b]		0.24 ± 0.32		0.05 ± 0.33		0.89 ± 0.82	
CCI-web vs. usual care[b]		-0.2 ± 0.38		-0.09 ± 0.43		-0.68 ± 0.81	
CCI-onsite vs. usual care[b]		-0.44 ± 0.39		-0.13 ± 0.45		-1.57 ± 0.77*	
CCI-all vs. usual care[b]		-0.33 ± 0.36		-0.11 ± 0.4		-1.15 ± 0.68	
**Uric acid (µmol·L^{-1})**							
All	346	344.42 (86.85)	273	343.82 (86.25)	73	346.2 (91.01)	-2.38 ± 11.9
CCI-all education[a]	261	347.99 (86.85)	202	348.58 (86.25)	59	346.2 (89.82)	2.38 ± 13.09
CCI-web[a]	126	355.13 (87.44)	98	361.07 (89.82)	28	333.71 (77.93)	27.36 ± 17.25
CCI-onsite[a]	135	341.44 (85.66)	104	336.69 (80.9)	31	357.5 (99.34)	-20.82 ± 19.63
Usual care[a]	85	333.12 (87.44)	71	330.74 (85.66)	14	345.01 (98.75)	-14.28 ± 28.55
CCI-web vs. CCI-onsite[b]		13.68 ± 10.71		24.98 ± 11.9		-23.79 ± 23.2	
CCI-web vs. usual care[b]		22.01 ± 12.49		30.34 ± 13.68*		-11.3 ± 30.34	
CCI-onsite vs. usual care[b]		7.73 ± 11.9		5.35 ± 13.09*		12.49 ± 32.12	
CCI-all vs. usual care[b]		14.87 ± 10.71		17.25 ± 11.9		1.19 ± 29.15	
**TSH (mIU·L^{-1})**							
All	344	2.23 (1.62)	270	2.25 (1.65)	74	2.17 (1.51)	0.08 ± 0.2
CCI-all education[a]	259	2.32 (1.74)	200	2.31 (1.79)	59	2.38 (1.55)	-0.07 ± 0.24
CCI-web[a]	126	2.37 (1.91)	98	2.46 (2.11)	28	2.07 (0.91)	0.38 ± 0.27
CCI-onsite[a]	133	2.28 (1.56)	102	2.16 (1.42)	31	2.65 (1.93)	-0.49 ± 0.37
Usual care[a]	85	1.97 (1.16)	70	2.09 (1.16)	15	1.38 (1.03)	0.71 ± 0.3*
CCI-web vs. CCI-onsite[b]		0.09 ± 0.22		0.29 ± 0.26		-0.58 ± 0.39	
CCI-web vs. usual care[b]		0.4 ± 0.21		0.36 ± 0.25		0.69 ± 0.32	
CCI-onsite vs. usual care[b]		0.31 ± 0.19		0.07 ± 0.2		1.27 ± 0.44†	
CCI-all vs. usual care[b]		0.36 ± 0.17*		0.21 ± 0.19		1.0 ± 0.33†	

**Free T4 (pmol·L^{-1})**							
All	346	11.71 (2.7)	273	11.71 (2.83)	73	11.46 (2.19)	0.26 ± 0.26
CCI-all education[a]	260	11.84 (2.19)	202	11.84 (2.32)	58	11.58 (2.19)	0.26 ± 0.39
CCI-web[a]	125	11.84 (2.06)	98	11.84 (1.93)	27	12.1 (2.06)	-0.26 ± 0.51
CCI-onsite[a]	135	11.84 (2.45)	104	11.97 (2.57)	31	11.2 (2.19)	0.77 ± 0.51
Usual care[a]	86	11.33 (3.73)	71	11.33 (3.86)	15	10.94 (2.32)	0.39 ± 0.77
CCI-web vs. CCI-onsite[b]		0.0 ± 0.26		-0.26 ± 0.26		0.77 ± 0.51	
CCI-web vs. usual care[b]		0.51 ± 0.39		0.51 ± 0.51		1.03 ± 0.77	
CCI-onsite vs. usual care[b]		0.51 ± 0.39		0.64 ± 0.51		0.26 ± 0.77	
CCI-all vs. usual care[b]		0.51 ± 0.39		0.51 ± 0.51		0.64 ± 0.64	
**Any diabetes medication, excluding metformin (%)**							
All	349	59.31 ± 2.63	291	58.76 ± 2.89	58	60.07 ± 6.37	-3.31 ± 6.99
CCI-all education[a]	262	56.87 ± 3.06	218	55.50 ± 3.37	44	63.64 ± 7.25	-8.13 ± 8.00
CCI-web[a]	126	57.14 ± 4.41	104	56.73 ± 4.86	22	59.09 ± 10.48	-2.36 ± 11.55
CCI-onsite[a]	136	56.62 ± 4.25	114	54.39 ± 4.66	22	68.18 ± 9.93	-13.80± 10.97†
Usual care[a]	87	66.67 ± 5.05	73	68.49 ± 5.44	14	57.14 ± 13.23	11.35 ± 14.32
CCI-web vs. CCI-onsite[b]		0.53 ± 6.12		2.34 ± 6.74		-9.09 ± 14.44	
CCI-web vs. usual care[b]		-9.52 ± 6.71		-11.76 ± 7.29		1.95 ± 16.88	
CCI-onsite vs. usual care[b]		-10.05 ± 6.60		-14.11 ± 7.16*		11.04 ± 16.54	
CCI-all vs. usual care[b]		-9.80 ± 5.91		-12.99± 6.39*		6.49 ± 15.08	
**Sulfonylurea (%)**							
All	349	23.78 ± 2.28	291	24.05 ± 2.51	58	22.41 ± 5.48	1.64 ± 6.02
CCI-all education[a]	262	23.66 ± 2.63	218	24.31 ± 2.91	44	20.45 ± 6.08	3.86 ± 6.74
CCI-web[a]	126	23.81 ± 3.79	104	25.96 ± 4.3	22	13.64 ± 7.32	12.33 ± 8.49
CCI-onsite[a]	136	23.53 ± 3.64	114	22.81 ± 3.93	22	27.27 ± 9.5	-4.47 ± 10.28
Usual care[a]	87	24.14 ± 4.59	73	23.29 ± 4.95	14	28.57 ± 12.07	-5.28 ± 13.05
CCI-web vs. CCI-onsite[b]		0.28 ± 5.26		3.15 ± 5.82		-13.64 ± 11.99	
CCI-web vs. usual care[b]		-0.33 ± 5.95		2.67 ± 6.55		-14.94 ± 14.12	
CCI-onsite vs. usual care[b]		-0.61 ± 5.85		-0.48 ± 6.32		-1.30 ± 15.36	
CCI-all vs. usual care[b]		-0.48 ± 5.29		1.02 ± 5.74		-8.12± 13.52	
**Insulin (%)**							
All	349	33.81 ± 2.53	296	34.12 ± 2.76	53	32.08 ± 6.41	2.04 ± 6.98
CCI-all education[a]	262	29.77 ± 2.82	218	28.44 ± 3.06	44	36.36 ± 7.25	-7.92 ± 7.87
CCI-web[a]	126	28.57 ± 4.02	104	27.88 ± 4.4	22	31.82 ± 9.93	-3.93 ± 10.86
CCI-onsite[a]	136	30.88 ± 3.96	114	28.95 ± 4.25	22	40.91 ± 10.48	-11.96 ± 11.31
Usual care[a]	87	45.98 ± 5.34	78	50.0 ± 5.66	9	11.11 ± 10.48	38.89 (1.91)‡
CCI-web vs. CCI-onsite[b]		-2.31 ± 5.65		-1.06 ± 6.11		-9.09 ± 14.44	

CCI-web vs. usual care[b]		-17.41 ± 6.69†		-22.12 ± 7.17†		20.71 ± 14.43	
CCI-onsite vs. usual care[b]		-15.09 ± 6.65*		-21.05 ± 7.08†		29.8 ± 14.82*	
CCI-all vs. usual care[b]		-16.21 ± 6.04†		-21.56 ± 6.43‡		25.25 ± 12.74*	
**Thiazolidinedione (%)**							
All	349	1.43 ± 0.64	291	1.72 ± 0.76	58	0.0 ± 0.0	1.72 ± 0.76*
CCI-all education[a]	262	1.53 ± 0.76	218	1.83 ± 0.91	44	0.0 ± 0.0	1.83 ± 0.91*
CCI-web[a]	126	2.38 ± 1.36	104	2.88 ± 1.64	22	0.0 ± 0.0	2.88 ± 1.64
CCI-onsite[a]	136	0.74 ± 0.73	114	0.88 ± 0.87	22	0.0 ± 0.0	0.88 ± 0.87
Usual care[a]	87	1.15 ± 1.14	73	1.37 ± 1.36	14	0.0 ± 0.0	1.37 ± 1.36
CCI-web vs. CCI-onsite[b]		1.65 ± 1.54		2.01 ± 1.86		0.0 ± 0.0	
CCI-web vs. usual care[b]		1.23 ± 1.78		1.51 ± 2.13		0.0 ± 0.0	
CCI-onsite vs. usual care[b]		-0.41 ± 1.36		-0.49 ± 1.62		0.0 ± 0.0	
CCI-all vs. usual care[b]		0.38 ± 1.37		0.46 ± 1.64		0.0 ± 0.0	
**SGLT-2 (%)**							
All	349	11.17 ± 1.69	291	11.68 ± 1.88	58	8.62 ± 3.69	3.06 ± 4.14
CCI-all education[a]	262	10.31 ± 1.88	218	10.55 ± 2.08	44	9.09 ± 4.33	1.46 ± 4.81
CCI-web[a]	126	7.14 ± 2.29	104	7.69 ± 2.61	22	4.55 ± 4.44	3.15 ± 5.15
CCI-onsite[a]	136	13.24 ± 2.91	114	13.16 ± 3.17	22	13.64 ± 7.32	-0.48 ± 7.97
Usual care[a]	87	13.79 ± 3.7	73	15.07 ± 4.19	14	7.14 ± 6.88	7.93 ± 8.06
CCI-web vs. CCI-onsite[b]		-6.09 ± 3.7		-5.47 ± 4.1		-9.09 ± 8.56	
CCI-web vs. usual care[b]		-6.65 ± 4.35		-7.38 ± 4.94		-2.60 ± 8.19	
CCI-onsite vs. usual care[b]		-0.56 ± 4.7		-1.91 ± 5.25		6.49 ± 10.05	
CCI-all vs. usual care[b]		-3.48 ± 4.15		-4.52 ± 4.68		1.95 ± 8.13*	
**DPP-4 (%)**							
All	349	9.46 ± 1.57	291	9.62 ± 1.73	58	8.62 ± 3.69	1.00 ± 4.07
CCI-all education[a]	262	9.92 ± 1.85	218	10.09 ± 2.04	44	9.09 ± 4.33	1.0 ± 4.79
CCI-web[a]	126	10.32 ± 2.71	104	9.62 ± 2.89	22	13.64 ± 7.32	-4.02 ± 7.87
CCI-onsite[a]	136	9.56 ± 2.52	114	10.53 ± 2.87	22	4.55 ± 4.44	5.98 ± 5.29
Usual care[a]	87	8.05 ± 2.92	73	8.22 ± 3.21	14	7.14 ± 6.88	1.08 ± 7.60
CCI-web vs. CCI-onsite[b]		0.76 ± 3.7		-0.91 ± 4.08		9.09 ± 8.56	
CCI-web vs. usual care[b]		2.27 ± 3.98		1.40 ± 4.32		6.49 ± 10.05	
CCI-onsite vs. usual care[b]		1.51 ± 3.85		2.31 ± 4.31		-2.60 ± 8.19	
CCI-all vs. usual care[b]		1.87 ± 3.45		1.87 ± 3.81		1.95 ± 8.13	
**GLP-1 (%)**							
All	349	13.75 ± 1.84	291	13.75 ± 2.02	58	13.79 ± 4.53	-0.05 ± 4.96
CCI-all education[a]	262	13.36 ± 2.1	218	12.84 ± 2.27	44	15.91 ± 5.51	-3.07 ± 5.96

CCI-web[a]	126	12.7 ± 2.97	104	11.54 ± 3.13	22	18.18 ± 8.22	-6.64 ± 8.8
CCI-onsite[a]	136	13.97 ± 2.97	114	14.04 ± 3.25	22	13.64 ± 7.32	0.4 ± 8.01
Usual care[a]	87	14.94 ± 3.82	73	16.44 ± 4.34	14	7.14 ± 6.88	9.30 ± 8.14
CCI-web vs. CCI-onsite[b]		-1.27 ± 4.2		-2.5 ± 4.52		4.55 ± 11.01	
CCI-web vs. usual care[b]		-2.24 ± 4.84		-4.90 ± 5.35		11.04 ± 10.72	
CCI-onsite vs. usual care[b]		-0.97 ± 4.84		-2.40 ± 5.42		6.49 ± 10.05	
CCI-all vs. usual care[b]		-1.58 ± 4.36		-3.59 ± 4.89		8.77 ± 8.82	
**Metformin (%)**							
All	349	68.77 ± 2.48	291	69.07 ± 2.71	58	67.24 ± 6.16	1.83 ± 6.73
CCI-all education[a]	262	71.37 ± 2.79	218	71.56 ± 3.06	44	70.45 ± 6.88	1.11 ± 7.53
CCI-web[a]	126	69.84 ± 4.09	104	71.15 ± 4.44	22	63.64 ± 10.26	7.52 ± 11.18
CCI-onsite[a]	136	72.79 ± 3.82	114	71.93 ± 4.21	22	77.27 ± 8.93	-5.34 ± 9.88
Usual care[a]	87	60.92 ± 5.23	73	61.64 ± 5.69	14	57.14 ± 13.23	4.50 ± 14.40
CCI-web vs. CCI-onsite[b]		-2.95 ± 5.59		-0.78 ± 6.12		-13.64 ± 13.6	
CCI-web vs. usual care[b]		8.92 ± 6.64		9.51 ± 7.22		6.49 ± 16.74	
CCI-onsite vs. usual care[b]		11.87 ± 6.48		10.29 ± 7.08		20.13 ± 15.96	
CCI-all vs. usual care[b]		10.45 ± 5.93		9.92 ± 6.46		13.31 ± 14.91	
**Statin (%)**							
All	349	52.15 ± 2.67	291	52.58 ± 2.93	58	50.0 ± 6.57	2.58 ± 7.19
CCI-all education[a]	262	50.0 ± 3.09	218	51.83 ± 3.38	44	40.91 ± 7.41	10.93 ± 8.15
CCI-web[a]	126	47.62 ± 4.45	104	50.96 ± 4.9	22	31.82 ± 9.93	19.14 ± 11.07
CCI-onsite[a]	136	52.21 ± 4.28	114	52.63 ± 4.68	22	50.0 ± 10.66	2.63 ± 11.64
Usual care[a]	87	58.62 ± 5.28	73	54.79 ± 5.83	14	78.57 ± 10.97	-23.78 ± 12.42
CCI-web vs. CCI-onsite[b]		-4.59 ± 6.18		-1.67 ± 6.77		-18.18 ± 14.57	
CCI-web vs. usual care[b]		-11.0 ± 6.9		-3.83 ± 7.61		-46.75 ± 14.79†	
CCI-onsite vs. usual care[b]		-6.41 ± 6.8		-2.16 ± 7.47		-28.57 ± 15.29	
CCI-all vs. usual care[b]		-8.62 ± 6.12		-2.96 ± 6.74		-37.66 ± 13.24†	
**Blood pressure medication (%)**							
All	349	63.61 ± 2.58	291	63.92 ± 2.82	58	62.07 ± 6.37	1.85 ± 6.97
CCI-all education[a]	262	67.18 ± 2.90	218	68.35 ± 3.15	44	61.36 ± 7.34	6.98 ± 7.99
CCI-web[a]	126	65.08 ± 4.25	104	65.38 ± 4.67	22	63.64 ± 10.26	1.75 ± 11.27
CCI-onsite[a]	136	69.12 ± 3.96	114	71.05 ± 4.25	22	59.09 ± 10.48	11.96 ± 11.31
Usual care[a]	87	52.87 ± 5.35	73	50.68 ± 5.85	14	64.29 ± 12.81	-13.60 ± 14.08
CCI-web vs. CCI-onsite[b]		-4.04 ± 5.81		-5.67 ± 6.31		4.55 ± 14.67	
CCI-web vs. usual care[b]		12.21 ± 6.83		14.70 ± 7.48*		-0.65 ± 16.41	
CCI-onsite vs. usual care[b]		16.24 ± 6.66*		20.37 ± 7.23†		-5.19 ± 16.55	
CCI-all vs. usual care[b]		14.30 ± 6.09*		17.66 ± 6.65†		-2.92 ± 14.76	

**ACE or ARB (%)**							
All	349	26.65 ± 2.37	291	25.09 ± 2.54	58	34.48 ± 6.24	-9.4 ± 6.74
CCI-all education[a]	262	29.39 ± 2.81	218	27.98 ± 3.04	44	36.36 ± 7.25	-8.38 ± 7.86
CCI-web[a]	126	32.54 ± 4.17	104	30.77 ± 4.53	22	40.91 ± 10.48	-10.14 ± 11.42
CCI-onsite[a]	136	26.47 ± 3.78	114	25.44 ± 4.08	22	31.82 ± 9.93	-6.38 ± 10.74
Usual care[a]	87	18.39 ± 4.15	73	16.44 ± 4.34	14	28.57 ± 12.07	-12.13 ± 12.83
CCI-web vs. CCI-onsite[b]		6.07 ± 5.63		5.33 ± 6.09		9.09 ± 14.44	
CCI-web vs. usual care[b]		14.15 ± 5.89*		14.33 ± 6.27*		12.34 ± 15.99	
CCI-onsite vs. usual care[b]		8.08 ± 5.62		9.0 ± 5.95		3.25 ± 15.63	
CCI-all vs. usual care[b]		11.0 ± 5.02*		11.54 ± 5.3*		7.79 ± 14.08	
**Diuretics (%)**							
All	349	38.11 ± 2.6	291	37.11 ± 2.83	58	43.1 ± 6.5	-5.99 ± 7.09
CCI-all education[a]	262	40.84 ± 3.04	218	41.28 ± 3.33	44	38.64 ± 7.34	2.65 ± 8.06
CCI-web[a]	126	40.48 ± 4.37	104	41.35 ± 4.83	22	36.36 ± 10.26	4.98 ± 11.34
CCI-onsite[a]	136	41.18 ± 4.22	114	41.23 ± 4.61	22	40.91 ± 10.48	0.32 ± 11.45
Usual care[a]	87	29.89 ± 4.91	73	24.66 ± 5.04	14	57.14 ± 13.23	-32.49 ± 14.16*
CCI-web vs. CCI-onsite[b]		-0.7 ± 6.08		0.12 ± 6.68		-4.55 ± 14.67	
CCI-web vs. usual care[b]		10.59 ± 6.57		16.69 ± 6.98*		-20.78 ± 16.74	
CCI-onsite vs. usual care[b]		11.29 ± 6.47		16.57 ± 6.83*		-16.23 ± 16.88	
CCI-all vs. usual care[b]		10.95 ± 5.77		16.63 ± 6.05†		-18.51 ± 15.13	

[a]Mean and standard deviations for continuous variables, percents and standard errors for categorical variables
[b]Difference between means or percentages ± 1 standard error of the difference. Significant baseline difference between means or percentages at 0.05>P≥0.01 (*); 0.01>P≥0.001 (†); 0.001>P≥0.0001 (‡); and P<0.0001 (§).
